# Supplementary material for: In Planta, In Vitro and In Silico Studies of Chiral N6-Benzyladenine Derivatives: Discovery of Receptor-Specific S-Enantiomers with Cytokinin or Anticytokinin Activities
Source: Int J Mol Sci. 2022 Sep 26;23(19):11334. doi: 10.3390/ijms231911334 (PMC9569578; doi:10.3390/ijms231911334)
Supplement: Supplementary file 1 [file ijms-23-11334-s001.zip › ijms-1906485-supplementary.pdf]

In Planta, In Vitro and In Silico Studies of Chiral N^6-Benzyladenine Derivatives: Discovery of Receptor-Specific *S*-Enantiomers with Cytokinin or Anticytokinin Activities

Ekaterina M. Savelieva[1a], Anastasia A. Zenchenko[2a], Mikhail S. Drenichev[2], Anna A. Kozlova [2], Nikolay N. Kurochkin [2], Dmitry V. Arkhipov [1], Alexander O. Chizhov [3], Vladimir E. Oslovsky [2] and Georgy A. Romanov[1]*

[1] Timiryazev Institute of Plant Physiology, Russian Academy of Sciences, Botanicheskaya str. 35, 127276 Moscow, Russia;
[2] Engelhardt Institute of Molecular Biology, Russian Academy of Sciences, Vavilov str. 32, 119991 Moscow, Russia;
[3] Zelinsky Institute of Organic Chemistry, Russian Academy of Science, Leninsky pr. 47, 119991 Moscow, Russia;
[a] Authors contributed equally
***** Correspondence: gromanov@yahoo.com; gar@ippras.ru (G.A.R.)

Supplementary Materials

1. Chemical synthesis S2
1.1. General S2
1.2. Synthesis of nucleobases with chiral substitutions S3
1.3. Synthesis of nucleosides with chiral substituents S6
1.4. Synthesis of *O*-isobutyroyl protected nucleoside synthons S13
1.5. Ado^{BOM} and Ade^{BOM} S16
2. CD-spectra of enantiomers S18
3. NMR-spectroscopy S19
4. High resolution mass spectrometry (HRMS) S49
5. Plant-based data S69
6. Molecular modeling and docking S72

1. Chemical synthesis

1.1. General

The solvents and materials were reagent grade and were used without additional purification. Column chromatography was performed on silica gel (Kieselgel 60 Merck, 0.063-0.200 mm). TLC was performed on Alugram SIL G/UV254 (Macherey-Nagel) with UV visualization. ^{1}H and ^{13}C (with complete proton decoupling) NMR spectra were recorded on Bruker AMX 300 NMR instrument and are also given in Supplementary Part. ^{1}H-NMR-spectra were recorded at 300.1 MHz and ^{13}C-NMR-spectra at 75.5 MHz. Chemical shifts in ppm were measured relative to the residual solvent signals as internal standards ($CDCl_3$, ^{1}H: 7.3 ppm, ^{13}C: 77.2 ppm; DMSO-*d*6, ^{1}H: 2.5 ppm, ^{13}C: 39.5 ppm; CD_3OD, ^{1}H: 3.3 ppm, ^{13}C: 49.0 ppm). Spin-spin coupling constants (*J*) are given in hertz (Hz). Double-resonance technique was applied to assign the resonances (see Supplementary Part for more details). The high-resolution mass spectra (HRMS) were registered on a Bruker micrOTOF II instrument using electrospray ionization (ESI) [Belyakov, P.A., Kadentsev, V.I., Chizhov, A.O., Kolotyrkina, N.G., Shashkov, A.S., Ananikov, V.P., 2010. Mechanistic insight into organic and catalytic reactions by joint studies using mass spectrometry and NMR spectroscopy. Mendeleev Commun. 20, 125e131. https://doi.org/10.1016/j.mencom.2010.05.001]. The measurements were done in a positive ion mode (interface capillary voltage – 4500 V); mass range from *m*/*z* 50 to *m*/*z* 3000 Da; internal calibration was done with Electrospray Calibrant Solution (Fluka). A syringe injection was used for solutions in acetonitrile, or acetonitrile : water mixture, 50 : 50 % vol. (flow rate 3 ml/min). Nitrogen was applied as a dry gas; interface temperature was set at 180◦C. 6-Chloropurine (**1**) (CAS 87-42-3) and 2-amino-6-chloropurine (**4**) (CAS 10310-21-1) were purchased from Sigma-Aldrich (sigmaaldrich.com). 2,6-Dichloropurine (**3**) and 2-fluoro-6-chloropurine (**2**) and 1-(β-D-ribofuranosyl)-2-amino-6-chloropurine (**15**) were obtained according to the literature procedures, described in [Steklov, M. Y., Tararov, V. I., Romanov, G. A., Mikhailov, S. N. (2011). Facile synthesis of 8-azido-6-benzylaminopurine. Nucleosides, Nucleotides and Nucleic Acids, 30(7-8), 503-511; Y.L. Hu, X. Liu, M. Lu. Synthesis and biological activity of novel 6-substituted purine derivatives. J. Mex. Chem. Soc. 2010, 54(2), 74-78; Gerster, J. F., Jones, J. W., & Robins, R. K. (1963). Purine Nucleosides. IV. The Synthesis of 6-halogenated 9-β-D-ribofuranosylpurines from inosine and guanosine. J. Org. Chem., 28(4), 945-948].

1.2. Synthesis of nucleobases with chiral substitutions

***N*⁶-((*R*)-α-methylbenzyl)adenine (5a)**

A mixture of 6-chloropurine (**1**) (200 mg, 1.294 mmol), (*R*)-(+)-α-methylbenzylamine (0.41 ml, 3.24 mmol) and DIPEA (0.55 ml, 3.24 mmol) in *n*-BuOH (5 ml) was refluxed at 110°C for 4.5 h under stirring. A dissolution of initial 6-chloropurine was observed during heating of the mixture. The reaction was monitored by TLC on silica-gel in EtOAc/hexane – 80/20 (%). The reaction mixture was then cooled to ambient temperature, evaporated in vacuum and the residue was applied to chromatographic column with silica-gel (30 ml) for purification. The column was washed with CH_2Cl_2 and CH_2Cl_2/EtOH – 98/2 (%), the product was eluted with CH_2Cl_2/EtOH – 97/3 (%). Fractions, containing the product, were collected and evaporated in vacuum. The resulting product was recrystallized from water (5 ml), the obtained white precipitate was filtered, washed with cold ether (2×5 ml), dried on a vacuum pump for 20 min and then in vacuum desiccator over phosphorous pentoxide for 2 days. Yield 114 mg (37%) as a white powder. R_f = 0.081 (EtOAc/hexane – 80/20 (%)). M.p. 153-155°C. ^{1}H-NMR (300.1 MHz, DMSO-d_6): δ = 12.69 (br s, 1H, N9-H), 8.14 (s, 1H, H-8), 8.11 (s, 1H, H-2), 7.97 (br s, 1H, N6-H), 7.46-7.15 (m, 5H, H-Ph), 5.52 (br s, 1H, -CH), 1.53 (d, 3H, $J_{CH\text{-}CH3}$ = 7.0 Hz, CH_3). ^{13}C-NMR (75.5 MHz, CD_3OD): δ = 154.76 (C-6), 153.70 (C-2), 151.26 (C-4), 145.39 (C-1 Ph), 140.63 (C-8), 129.53 (Ph), 128.08 (Ph), 127.12 (Ph), 118.85 (C-5), 51.17 (NCH-), 23.12 (-CH_3). HRMS: *m/z* $[C_{13}H_{13}N_5+H]^+$ calculated 240.1244, found 240.1243, $[C_{13}H_{13}N_5+Na]^+$ calculated 262.1063, found 262.1059, $[C_{13}H_{13}N_5+K]^+$ calculated 278.0803, found 278.0802

***N*⁶-((*S*)-α-methylbenzyl)adenine (5b)**

The procedure was analogous to the preparation of (**5a**) from 6-chloropurine (**1**) (200 mg, 1.294 mmol), (*S*)-(–)-α-methylbenzylamine and DIPEA in *n*-BuOH (5 ml) at 110°C. Yield of **5b** was 173 mg (56%) as a white powder. R_f = 0.077 (EtOAc/hexane – 80/20 (%)). M.p. 152-154°C. ^{1}H-NMR (300.1 MHz, DMSO-d_6): δ = 12.07 (br s, 1H, N9-H), 8.13 (s, 1H, H-8), 8.12 (s, 1H, H-2), 7.97 (d 1H, $J_{NH\text{-}CH}$ = 8.3 Hz, N6-H), 7.46-7.15 (m, 5H, H-Ph), 5.53 (br s, 1H, -CH), 1.53 (d, 3H, $J_{CH\text{-}CH3}$ = 7.0 Hz, CH_3). ^{13}C-NMR (75.5 MHz, CD_3OD): δ = 154.75 (C-6), 153.78 (C-2), 151.31 (C-4), 145.39 (C-1 Ph), 140.67 (C-8), 129.52 (Ph), 128.08 (Ph), 127.12 (Ph), 118.85 (C-5), 51.16 (NCH-), 23.12 (-CH_3). HRMS: *m/z* $[C_{13}H_{13}N_5+H]^+$ calculated 240.1244, found 240.1245, $[C_{13}H_{13}N_5+Na]^+$ calculated 262.1063, found 262.1058, $[C_{13}H_{13}N_5+K]^+$ calculated 278.0803, found 278.0803

2-fluoro,*N*⁶-((*R*)-α-methylbenzyl)adenine (6a)

To 2-fluoro,6-chloropurine (**2**) (75.5 mg, 0.58 mmol) in *n*-BuOH (5 ml) (*R*)-(+)-α-methylbenzylamine (0.152 ml, 1.18 mmol) and DIPEA (0.202 ml, 1.18 mmol) were added in one portion and the solution was stirred at 60°C for 8 h. The reaction was monitored by TLC (silica gel, CH_2Cl_2/EtOH – 97/3 (%)). Then

the reaction mixture was evaporated and the residue was dissolved in EtOAc and washed with brine (3×20 ml). The organic layer was separated, dried over anhydrous sodium sulfate, filtered and evaporated in vacuum. The residue was applied to column chromatography on silica gel. The column was washed with CH_2Cl_2, the product was eluted with CH_2Cl_2/EtOH – 97/3 (%). The resulting product was dried for 24 hours in a vacuum desiccator over phosphorous pentaoxide for 2 days to yield 51 mg (34%) as a white powder. Yield. R_f = 0.41 (CH_2Cl_2/EtOH – 95/5 (%)). M.p. 210-213°C.^{1}H-NMR (300.1 MHz, DMSO-d_6): δ = 13.01 (s, 1H, N9-H), 8.68 (br s, 1H, N6-H), 8.08 (s, 1H, H-8), 7.46-7.17 (m, 5H, H-Ph), 5.36 (br s, 1H,-CH), 1.54 (d, 3H, $J_{CH\text{-}CH3}$ = 7.0 Hz, CH_3). ^{13}C-NMR (75.5 MHz, DMSO-d6): δ = 158.55 (d, $^1J_{C\text{-}F}$= 201.9 Hz, C-2), 155.10 (C-6), 150.73 (C-4) 144.48 (C-1 Ph), 139.11 (C-8), 128.24 (Ph), 126.69 (Ph), 126.14 (Ph), 117.09 (C-5), 49.18 (NCH-), 22.21 (-CH_3). HRMS: *m/z* $[C_{13}H_{12}FN_5+H]^+$ calculated 258.1150, found 258.1151, $[C_{13}H_{12}FN_5+Na]^+$ calculated 280.0969, found 280.0970, $[C_{13}H_{12}FN_5+K]^+$ calculated 296.0708, found 296.0688.

2-fluoro,*N*^{6}-((*S*)-α-methylbenzyl)adenine (6b)

The procedure was analogous to preparation of 2-fluoro,*N*^{6}-((*R*)-α-methylbenzyl)adenine (**6a**)using 2-fluoro,6-chloropurine (**2**) (75.5 mg, 0.58 mmol) and (*S*)-(–)-α-methylbenzylamine (0.152 ml, 1.18 mmol) in the presence of DIPEA (0.202 ml, 1.18 mmol) in *n*-BuOH (5 ml) at 60°C during 8 h with further chromatographic purification on silica gel. The product was eluted with CH_2Cl_2/EtOH – 97/3 (%) to give **6b** as a white powder. Yield 37 mg (25%). R_f = 0.41 (CH_2Cl_2/EtOH – 95/5 (%)). M.p. 202-204°C. ^{1}H-NMR (300.1 MHz, DMSO-d_6): δ = 12.98 (s, 1H, N9-H), 8.66 (br s, 1H, N6-H), 8.10 (s, 1H, H-8), 7.47-7.17 (m, 5H, H-Ph), 5.43-5.26 (m, 1H,-CH), 1.54 (d, 3H, $J_{CH\text{-}CH3}$ = 7.0 Hz, CH_3).^{13}C-NMR (75.5 MHz, DMSO-d6): δ = 158.55 (d, $^1J_{C\text{-}F}$ = 201.8 Hz, C-2), 154.95 (d, $^3J_{C\text{-}F}$ = 20.6 Hz, C-6), 150.82 (d, $^3J_{C\text{-}F}$ = 20.4 Hz, C-4), 144.61 (C-1 Ph), 139.10 (C-8), 128.22 (Ph), 126.65 (Ph), 126.14 (Ph), 117.09 (C-5), 49.14 (NCH-), 22.20 (-CH3). HRMS: *m/z* $[C_{13}H_{12}FN_5+H]^+$ calculated 258.1150, found 258.1151, $[C_{13}H_{12}FN_5+Na]^+$ calculated 280.0969, found 280.0969, $[C_{13}H_{12}FN_5+K]^+$ calculated 296.0708, found 296.0707.

2-chloro,*N*^{6}-((*R*)-α-methylbenzyl)adenine (7a)

The procedure was analogous to preparation of 2-fluoro,*N*^{6}-((*R*)-α-methylbenzyl)adenine (**6a**) using 2,6-dichloropurine (**3**) (200 mg, 1.06 mmol) and (*R*)-(+)-α-meth(+)ylbenzylamine (0.405 ml, 3.18 mmol) in the presence of DIPEA (0.544 ml, 3.18 mmol) in *n*-BuOH (5 ml) at 60°C during 8 h with further chromatographic purification on silica gel. The product was eluted with CH_2Cl_2/EtOH – 97/3 (%) to give **7a** as a white powder. Yield 144 mg (50%). R_f = 0.40 (CH_2Cl_2/EtOH – 95/5 (%)). M.p. 204-205°C (decomposition with HCl emission). ^{1}H-NMR (300.1 MHz, DMSO-d_6): δ = 12.90 (s, 1H, N9-H), 8.53 (br s, 1H, N6-H), 8.14 (s, 1H, H-8) 7.46-7.16 (m, 5H, H-Ph), 5.39 (br s, 1H,-CH), 1.53 (d, 3H, $J_{CH\text{-}CH3}$ = 7.0 Hz, CH_3). ^{13}C-NMR (75.5 МГц, DMSO-d6): δ = 153.43 (C-6), 152.77 (C-2), 149.72 (C-4), 144.42 (C-1

Ph), 140.01 (C-8), 128.23 (Ph), 126.70 (Ph), 126.18 (Ph), 116.63 (C-5), 49.00 (NCH-), 22.10 (-CH3). HRMS: *m/z* $[C_{13}H_{12}ClN_5+H]^+$ calculated 274.0854, found 274.0851.

2-chloro,*N*^{6}-((S)-α-methylbenzyl)adenine (7b)

The procedure was analogous to preparation of 2-fluoro,*N*^{6}-((*R*)-α-methylbenzyl)adenine (**6a**) using 2,6-dichloropurine (**3**) (200 mg, 1.06 mmol) and (*S*)-(–)-α-methylbenzylamine (0.410 ml, 3.18 mmol) in the presence of DIPEA (0.544 ml, 3.18 mmol) in *n*-BuOH (5 ml) at 60°C during 8 h with further chromatographic purification on silica gel. The product was eluted with CH_2Cl_2/EtOH – 97/3 (%) to give **7b** as a white powder. Yield 179 mg (62%). R_f = 0.40 (CH_2Cl_2/EtOH – 95/5 (%)). M.p. 191-192°C (decomposition with HCl emission). ^{1}H-NMR (300.1 MHz, DMSO-d_6): δ = 13.02 (s, 1H, N9-H), 8.58 (br s, 1H, N6-H), 8.14 (s, 1H, H-8) 7.46-7.18 (m, 5H, H-Ph), 5.39 (br s, 1H,-CH), 1.53 (d, 3H, J_{CH-CH3} = 7.0 Hz, CH3). ^{13}C-NMR (75.5 MHz, DMSO-d6): δ = 154.90 (C-6), 152.76 (C-2), 149.89 (C-4), 144.42 (C-1 Ph), 139.33 (C-8), 128.23 (Ph), 126.70 (Ph), 126.18 (Ph), 116.69 (C-5), 49.00 (NCH-), 22.10 (-CH_3). HRMS: *m/z* $[C_{13}H_{12}ClN_5+H]^+$ calculated 274.0854, found 274.0853, $[C_{13}H_{12}ClN_5+Na]^+$ calculated 296.0663, found 296.0663, $[C_{13}H_{12}ClN_5+K]^+$ calculated 312.0413, found 312.0412.

2-amino, *N*^{6}-((*R*)-α-methylbenzyl)adenine (8a)

A mixture of 2-amino-6-chloropurine (**1**) (200 mg, 1.183 mmol), (*R*)-(+)-α-methylbenzylamine (0.38 ml, 2.96 mmol) and DIPEA (0.5 ml, 2.96 mmol) in *n*-BuOH (5 ml) was refluxed at 120°C for 6.5 h under stirring until the formation of slightly brown solution. The reaction was monitored by TLC on silica-gel in ethyl EtOAc/hexane – 70/30 (%). After full conversion of 6-chloropurine according to TLC the resulting mixture was quenched by Et_2O (5 ml) and left to stay at -25°C overnight. The obtained low-disperse precipitate was filtered through Teflon membrane Phenomenex (diameter 47 mm, pore diameter 0.45 µm) and was then applied to chromatographic column with silica-gel for purification. The column was washed with CH_2Cl_2 with increasing in polarity to CH_2Cl_2/EtOH – 98/2 (%), the product was eluted with CH_2Cl_2/EtOH – 95/5 (%). Fractions, containing the product, were collected and evaporated in vacuum to dryness. The residue was dried in vacuum desiccator over phosphorous pentoxide for 2 days to yield 61 mg (20%) as a slightly yellow powder. R_f = 0.081 (EtOAc/hexane – 80/20 (%)). M.p. 224-226°C. ^{1}H-NMR (300.1 MHz, DMSO-d_6): δ = 12.81 (br s, 1H, N9-H), 8.43 (br s, 1H, N^6-H), 7.89 (s, 1H, H-8), 7.47-7.17 (m, 5H, H-Ph), 6.43 (br s, 2H, NH_2), 5.50 (br s, 1H, -CH), 1.53 (d, 3H, J_{CH-CH3} = 7.0 Hz, CH_3). ^{13}C-NMR (75.5 MHz, $CD_3OD/CDCl_3$ – 5/1 (*v/v*)): δ = 158.53 (C-2), 153.92 (C-6), 150.43 (C-4), 144.25 (C-1 Ph), 138.89 (C-8), 129.25 (Ph), 127.92 (Ph), 126.86 (Ph), 110.41 (C-5), 50.75 (NCH-), 22.66 (-CH_3). HRMS: *m/z* $[C_{13}H_{14}N_6+H]^+$ calculated 255.1353, found 255.1353.

2-amino,*N*^{6}-((*S*)-α-methylbenzyl)adenine (8b)

A mixture of 2-amino-6-chloropurine (**1**) (200 mg, 1.183 mmol), (*R*)-(+)-α-methylbenzylamine (0.38 ml, 2.96 mmol) and DIPEA (0.5 ml, 2.96 mmol) in *n*-BuOH (5 ml) was refluxed at 120°C for 6.5 h under stirring until the formation of slightly brown solution. The reaction was monitored by TLC on silica-gel in EtOAc/hexane – 70/30 (%). After full conversion of 6-chloropurine according to TLC the resulting mixture was cooled to ambient temperature and evaporated to near dryness. The residue was then applied to chromatographic column with silica-gel for purification. The column was washed with CH_2Cl_2 with increasing in polarity to CH_2Cl_2/EtOH – 97/3 (%), the product was eluted with CH_2Cl_2/EtOH in EtOH gradient from 5 to 8%. Fractions, containing the product, were collected and evaporated in vacuum to dryness. The residue was then precipitated from ether at -25°C, filtered through a Schott glass filter, dried on a vacuum pump (bath temperature 50°C) for 3 hrs and then in vacuum desiccator over phosphorous pentoxide for 2 days to yield 114 mg (38%) as a yellow powder. R_f = 0.10 (CH_2Cl_2/EtOH – 95/5 (%)). M.p. 85-87°C (decomposition with NH_3 emission). ^{1}H-NMR (300.1 MHz, DMSO-d_6): δ = 12.78 (br s, 1H, N9-H), 8.45 (br s, 1H, N^6-H), 7.92 (s, 1H, H-8), 7.51-7.20 (m, 5H, H-Ph), 6.54 (br s, 2H, NH_2), 5.50 (br s, 1H, -CH), 1.53 (d, 3H, $J_{CH\text{-}CH3}$ = 7.0 Hz, CH_3).^{13}C-NMR (75.5 MHz, $CD_3OD/CDCl_3$ – 5/1 (*v*/*v*))):): δ = 161.19 (C-2), 154.90 (C-6), 152.01 (C-4), 144.94 (C-1 Ph), 136.82 (C-8), 129.16 (Ph), 127.68 (Ph), 126.75 (Ph), 113.36 (C-5), 50.21 (NCH-), 23.03 (-CH_3). HRMS: *m/z* $[C_{13}H_{14}N_6+H]^+$ calculated 255.1353, found 255.1352.

1.3. Synthesis of nucleosides with chiral substituents

O^6-(benzotriazol-1-yl)-2′,3′,5′-tri-*O*-isobutyroyl inosine (10)

To a solution of 2′,3′,5′-tri-O-isobutyroyl inosine **(9)** (300 mg, 0.63 mmol) and BOP (415 mg, 0.94 mmol) in anhydrous MeCN (5 ml) was added DBU (0.233 ml, 1.58 mmol) and the resulting mixture was kept at ambient temperature for 2 hours. The reaction was monitored by TLC (silica gel, CH_2Cl_2/EtOH − 97/3 (%)). The obtained solution was concentrated in vacuum and the residue was dissolved in EtOAc (30 ml) and washed with brine (3 × 30 ml). The organic layer was separated, dried over anhydrous sodium sulfate, filtered, and evaporated in vacuum to near dryness. The residue was poured into chromatographic column with silica-gel. The column was washed with CH_2Cl_2, the product was eluted with CH_2Cl_2/EtOH – 97/3 (%). The resulting product was dried for 24 hours in a vacuum desiccator over phosphorous pentaoxide (P_2O_5) for 2 days to yield 359 mg (96%) as a white foam. R_f =0.44 (CH_2Cl_2/EtOH, 97/3 (%)). ^{1}H-NMR (300.1*MHz*, $CDCl_3$): δ = 8.43 (s, 1H, H-8), 8.32 (s, 1H, H-2), 8.13 (d, 1H, ^3J = 8.3, Ar-H-benzotriazol), 7.59-7.40 (m, 3H, Ar-H-benzotriazol), 6.27 (d, 1H, $J_{1',2'}$= 5.2, H-1′), 5.91 (dd, 1H, $J_{2',1'}$= 5.2, $J_{2',3'}$= 5.4, H-2′), 5.64 (dd, 1H, $J_{3',2'}$= 5.4, $J_{3',4'}$= 4.8, H-3′), 4.49 (dd, 1H, $J_{4',3'}$= 4.8, $J_{4',5'}$= 3.7, H-4′), 4.42 (d, 2H, $J_{5',4'}$= 3.7, H-5′), 2.70-2.50 (m, 3H, C*H*-*i*Bu), 1.22 (d, 6H, ^3J = 7.0, CH_3-*i*Bu), 1.21-1.16 (m, 9H, CH_3-*i*Bu), 1.12 (d, 3H, ^3J = 7.1, CH_3-*i*Bu). ^{13}C-NMR (75.5 MHz, CDCl3) δ = 176.60 (C=O), 175.73 (C=O), 175.55 (C=O), 159.42 (C-2), 153.82 (C-6), 152.03 (C-4), 143.37 (C-8), 128.97 (C-BOP), 125.04

(C-BOP), 120.71 (C-BOP, C-5), 108.78 (C-BOP), 87.23 (C-1′), 81.10 (C-2′), 73.47 (C-4′), 70.53 (C-3′), 63.06 (C-5′), 34.06 (*C*H-*i*Bu), 33.91 (*C*H-*i*Bu), 33.80 (*C*H-*i*Bu), 19.12 (CH$_3$-*i*Bu), 19.01 (CH$_3$-*i*Bu), 18.99 (CH$_3$-*i*Bu), 18.93 (CH$_3$-*i*Bu), 18.87 (CH$_3$-*i*Bu), 18.78 (CH$_3$-*i*Bu).

N^6-((*R*)-α-methylbenzyl)-2′,3′,5′-tri-*O*-isobutyroyl adenosine (11a)

To O^6-benzotriazolyl-2′,3′,5′-tri-*O*-isobutyroyl inosine **(10)** (300 mg, 0.5 mmol) in anhydrous MeCN (10 ml) (*R*)-(+)-α-methylbenzylamine (0.25 ml, 2 mmol) was added, and the resulting mixture was kept at ambient temperature for 20 hours. The reaction was monitored by TLC (silica gel, EtOAc/hexane – 1/1 (v/v). During the reaction the formation of white precipitate of benzotriazole (BTA) was observed. After 20 hrs the precipitate was filtered through the glass filter, washed with MeCN and the mother solution containing the resulting product was evaporated in vacuum. The residue was dissolved in EtOAc/hexane – 1/1 (v/v) and poured into chromatographic column with silica-gel. The product was eluted with EtOAc/hexane – 1/1 (v/v). The resulting product was dried for 24 hours in a vacuum desiccator over phosphorous pentaoxidefor 2 days to yield 245 mg (84%) as a yellow syrup. R_f = 0.50 (EtOAc/hexane – 1/1 (v/v)). ^{1}H-NMR (300.1 MHz, $CDCl_3$): δ = 8.36 (s, 1H, H-8), 7.91 (s, 1H, H-2), 7.42 (dd, 2H, ^3J = 7.2, ^4J = 1.6, *ortho*-H, Ph), 7.37-7.29 (m, 2H, *meta*-H, Ph), 7.25 (tt, 1H, ^3J = 7.1, ^4J = 1.6, *para*-H, Ph, overlapping with the solvent residual peak), 6.18 (d, 1H, $J_{1′,2′}$= 5.5, H-1′), 6.03 (br d, 1H, $J_{NH\text{-}CH}$ = 8.1, N*H*), 5.87 (dd, 1H, $J_{1′,2′}$=$J_{2′,3′}$ = 5.5, H-2′), 5.65 (dd, 1H, $J_{3′,2′}$ = 5.5, $J_{3′,4′}$= 4.0, H-3′), 5.62 (br s, 1H, C*H*, overlaps with H-3′), 4.46-4.37 (m, 3H, H-4′, H-5′a, H-5′b), 2.62 (sept, 2H, ^3J = 7.0, C*H*-*i*Bu), 2.55 (sept, 1H, ^3J = 7.0, C*H*-*i*Bu), 1.65 (d, 3H, ^3J = 6.9, CH_3-CHNH), 1.24-1.19 (m, 9H, CH_3-*i*Bu). 1.17 (d, 3H, ^3J = 7.0, CH_3-*i*Bu), 1.15 (d, 3H, ^3J = 7.0, CH_3-*i*Bu), 1.12 (d, 3H, ^3J = 7.0, CH_3-*i*Bu). ^{13}C-NMR (75.5 MHz, $CDCl_3$): δ = 176.66 (C=O), 175.78 (C=O), 175.56 (C=O), 154.19 (C-2), 153.71 (C-6), 143.70 (C-8), 137.92 (C-1 Ph), 128.77 (Ph), 127.42 (Ph), 126.28 (Ph), 86.19 (C-1′), 80.79 (C-2′), 73.38 (C-4′), 70.70 (C-3′), 63.30 (C-5′), 50.05 (*C*H-Ph), 34.08 (*C*H-*i*Bu), 33.93 (*C*H-*i*Bu), 33.81 (*C*H-*i*Bu), 22.68 (CH$_3$-CH-Ph), 19.13, 19.03, 18.97, 18.90, 18,79 (CH$_3$-*i*Bu).

N^6-((*R*)-α-methylbenzyl)adenosine (12a)

Tri-*O*-isobutyroyl protected nucleoside **11a** (243 mg, 0.42 mmol) was treated with 4M $MeNH_2$ in EtOH solution (12 ml) and the reaction was kept at ambient temperature for 18 hours. The reaction was monitored by TLC (CH_2Cl_2/EtOH – 95/5 (%)). The resulting mixture was evaporated in vacuum and the residue was applied to column chromatography on silica gel. The column was washed using CH_2Cl_2: EtOH gradient, increasing in polarity from CH_2Cl_2/EtOH – 98/2 (%)) to CH_2Cl_2/EtOH – 95/5 (%)). The product was eluted with CH_2Cl_2/EtOH – 92/8 (%)). The resulting product was dried for 24 hrs in a vacuum desiccator over phosphorous pentaoxide (P_2O_5) to yield 127 mg (82%) as a white powder. R_f =0.15 (CH_2Cl_2/EtOH − 95/5 (%)). M.p. 167-170°C (with decomposition). ^{1}H-NMR (300.1 MHz, DMSO-d_6): δ = 8.38 (s, 1H, H-8), 8.29 (br d, *J* = 7.6, 1H, NH), 8.17 (s, 1H, H-2), 7.45 (br d, 2H, ^3J = 7.2, *ortho*-

H, Ph), 7.30 (dd, 2H, $^3J_1$ = 7.2, $^3J_2$ = 7.7, *meta*-H, Ph), 7.19 (tt, 1H, $^3J_1$ = 7.2, $^4J_1$ = 1.3, *para*-H, Ph), 5.89 (d, 1H, $J_{1',2'}$ = 6.2, H-1′), 5.66 – 5.44 (br s, 1H, NC*H*), 5.40 (d, 1H, $J_{OH,2'}$ = 6.2, 2′-OH), 5.36 (dd, 1H, $J_{OH,5'a}$ = 7.0, $J_{OH,5'b}$ = 4.6, 5'-OH), 5.15 (d, 1H, $J_{OH,3'}$ = 4.6, 3'-OH), 4.61 (td, 1H, $J_{2',1'}$ = 6.2, $J_{3',2'}$ = 5.2, $J_{2',OH}$ = 6.2, H-2′), 4.15 (ddd, 1H, $J_{3',2'}$ = 5.2, $J_{3',OH}$ = 4.6, $J_{3',4'}$ = 3.1, H-3′), 3.96 (ddd, 1H, $J_{4',5'a}$ = 4.2, $J_{4',5'b}$ = 3.6, $J_{4',3'}$ = 3.1, H-4′), 3.68 (ddd, 1H, $J_{5'b,5'a}$ = -12.0, $J_{5'b,OH}$ = 4.6, $J_{5'b,4'}$ = 3.6, H-5′b), 3.55 (ddd, 1H, $J_{5'a,5'b}$ = -12.0, $J_{5'a,OH}$ = 7.2, $J_{5'a,4'}$ = 4.2, H-5′a), 1.55 (d, ^3J = 7.0, 3H, CH_3).^{13}C-NMR (75.5 MHz, DMSO-d_6): δ = 153.94 (C-2), 152.21 (C-6), 148.55 (C-4), 145.13 (C(Ph)-CH), 139.78 (C-8), 128.14 (*C*-1, Ph), 126.48 (Ph), 126.11 (Ph), 119.74 (C-5), 87.98 (C-1′), 85.89 (C-4′), 73.41 (C-2′), 70.64 (C-3′), 61.66 (C-5′), 48.83 (*C*HPh), 22.49 (CH_3). HRMS: *m/z* $[C_{18}H_{21}N_5O_4+H]^+$ calculated 372.1666, found 372.1659, $[C_{18}H_{21}N_5O_4+Na]^+$ calculated 394.1486, found 394.1481, $[C_{18}H_{21}N_5O_4+K]^+$ calculated 410.1225, found 410.1235

N^6-((*S*)-α-methylbenzyl)-2′,3′,5′-tri-*O*-isobutyroyl adenosine (11b)

The procedure was analogous to preparation of N^6-((*R*)-α-methylbenzyl)-2′,3′,5′-tri-*O*-isobutyroyl adenosine (**11a**) using O^6-benzotriazolyl-2′,3′,5′-tri-*O*-isobutyroyl inosine (**10**) (390 mg, 0.66 mmol) and (*S*)-(–)-α-methylbenzylamine (0.34 ml, 1.31 mmol) in anhydrous MeCN (10 ml) at ambient temperature during 20 h with further chromatographic purification on silica gel. The product was eluted with EtOAc/hexane – 1/1 (v/v) to give **11b** as a yellow syrup. Yield 303 mg (79%). R_f = 0.50 (EtOAc/hexane – 1/1 (v/v)). ^{1}H-NMR (300.1 MHz, $CDCl_3$): δ = 8.36 (s, 1H, H-8), 7.91 (s, 1H, H-2), 7.42 (dd, 2H, ^3J = 7.2, ^4J = 1.6, *ortho*-H, Ph), 7.33 (m, 2H, *meta*-H, Ph), 7.25 (tt, 1H, ^3J = 7.1, ^4J = 1.4, *para*-H, Ph, overlapping with the solvent residual peak), 6.18 (d, 1H, $J_{1',2'}$= 5.5, H-1′), 6.08 (br d, 1H, $J_{NH\text{-}CH}$ = 8.1, N*H*), 5.87 (dd, 1H, $J_{1',2'}$=$J_{2',3'}$ = 5.5, H-2′), 5.66 (dd, 1H, $J_{3',2'}$ = 5.5, $J_{3',4'}$= 4.1, H-3′), 5.61 (br s, 1H, C*H*, overlaps with H-3′), 4.46-4.37 (m, 3H, H-4′, H-5′a, H-5′b), 2.69-2.53 (m, 2H, C*H*-*i*Bu), 2.54 (sept, 1H, ^3J = 7.0, C*H*-*i*Bu), 1.65 (d, 3H, ^3J = 6.9, CH_3-CHNH), 1.24-1.19 (m, 9H, CH_3-*i*Bu). 1.17 (d, 3H, ^3J = 7.0, CH_3-*i*Bu), 1.15 (d, 3H, ^3J = 7.0, CH_3-*i*Bu), 1.12 (d, 3H, ^3J = 7.0, CH_3-*i*Bu). ^{13}C-NMR (75.5 MHz, $CDCl_3$): δ = 176.63 (C=O), 175.75 (C=O), 175.54 (C=O), 154.17 (C-2), 153.67 (C-6), 143.73 (C-8), 137.95 (*C-1 Ph*), 128.72 (Ph), 127.36 (Ph), 126.26 (Ph), 120.14 (C-5), 86.23 (C-1′), 80.75 (C-2′), 73.35 (C-4′), 70.67 (C-3′), 63.27 (C-5′), 50.00 (*C*H-Ph), 34.03 (*C*H-*i*Bu), 33.90 (*C*H-*i*Bu), 33.78 (*C*H-*i*Bu), 22.66 (*C*H_3-CH-Ph), 19.09, 18.99, 18.97, 18.94, 18.88, 18,76 (*C*H_3-*i*Bu).

N^6-((*S*)-α-methylbenzyl)adenosine (12b)

The procedure was analogous to preparation of N^6-((*R*)-α-methylbenzyl)adenosine (**12a**) starting from N^6-((*S*)-α-methylbenzyl)-2′,3′,5′-tri-*O*-isobutyroyl adenosine (**11b**)(276 mg, 0.48 mmol) in the presence of 4M $MeNH_2$ in EtOH solution (12 ml) at ambient temperature during 18 hrs with further chromatographic purification on silica gel. The product was eluted with CH_2Cl_2/EtOH – 92/8 (%) to give **12b** as a white powder. Yield 167 mg (94%). R_f =0.15 (CH_2Cl_2/EtOH – 95/5 (%)). M.p. 135-136°C. ^{1}H-NMR (300.1

MHz, DMSO-d_6): δ = 8.38 (s, 1H, H-8), 8.29 (br d, J = 8.1, 1H, NH), 8.17 (s, 1H, H-2), 7.45 (dd, 2H, ^3J = 7.3, ^4J = 1.3, *ortho*-H, Ph), 7.29 (t, 2H, $^3J_1$ = 7.3, $^3J_2$ = 7.3, *meta*-H, Ph), 7.19 (tt, 1H, $^3J_1$ = 7.3, $^4J_1$ = 1.3, *para*-H, Ph), 5.89 (d, 1H, $J_{1',2'}$ = 6.1, H-1′), 5.66 – 5.44 (br s, 1H, NC*H*), 5.41 (d, 1H, $J_{OH,2'}$ = 6.1, 2′-OH), 5.36 (dd, 1H, $J_{OH,5'a}$ = 7.0, $J_{OH,5'b}$ = 4.6, 5'-OH), 5.15 (d, 1H, $J_{OH,3'}$ = 4.7, 3'-OH), 4.60 (td, 1H, $J_{2',1'}$ = 6.1, $J_{2',3'}$ = 4.7, $J_{2',OH}$ = 6.1, H-2′), 4.15 (ddd, 1H, $J_{3',2'}$ = 4.7, $J_{3',OH}$ = 4.7, $J_{3',4'}$ = 3.2, H-3′), 3.97 (ddd, 1H, $J_{4',5'a}$= 4.2, $J_{4',5'b}$ = 3.7, $J_{4',3'}$ = 3.1, H-4′), 3.68 (ddd, 1H, $J_{5'b,5'a}$ = -12.0, $J_{5'b,OH}$ = 4.6, $J_{5'b,4'}$ = 3.7, H-5′b), 3.55 (ddd, 1H, $J_{5'a,5'b}$ = -12.0, $J_{5'a,OH}$ = 7.0, $J_{5'a,4'}$ = 3.8, H-5′a), 1.55 (d, ^3J = 7.0, 3H, CH_3).^{13}C-NMR (75.5 MHz, DMSO-d_6): δ = 153.89 (C-2), 152.22 (C-6), 148.55 (C-4), 145.16 (C(Ph)-CH), 139.68 (C-8), 128.13 (*C*-1, Ph), 126.47 (Ph), 126.10 (Ph), 119.55 (C-5), 87.86 (C-1′), 85.82 (C-4′), 73.53 (C-2′), 70.59 (C-3′), 61.60 (C-5′), 48.79 (*C*HNH), 22.45 (CH_3). HRMS: *m/z* $[C_{18}H_{21}N_5O_4+H]^+$ calculated 372.1666, found 372.1659, $[C_{18}H_{21}N_5O_4+Na]^+$ calculated 394.1486, found 394.1481, $[C_{18}H_{21}N_5O_4+K]^+$ calculated 410.1225, found 410.1235

2-fluoro,N^6-((*R*)-α-methylbenzyl)adenosine (13a)

The mixture of 2-fluoro,N^6-((*R*)-α-methylbenzyl)adenine **6a** (47 mg, 0.183 mmol), 1,2,3,5-tetra-*O*-acetyl-*β*-D-ribofuranose (116 mg, 0.366 mmol) and *N,O*-bis(trimethylsilyl) acetamide (BSA) (0.179 ml, 0.732 mmol) in the anhydrous MeCN (5 ml) was stirred at 60°C in the N_2 atmosphere. After 30 minutes trimethylsilyl trifluoromethanesulfonate (TMSOTf) (0.132 ml, 0.732 mmol) was added to the mixure and the reaction was stirred at 60°C in the N_2 atmosphere for 8 h. The reaction was monitored by TLC (silica gel, CH_2Cl_2/EtOH – 97/3 (%)). Then the reaction mixture was diluted with CH_2Cl_2 (10 ml) and washed with 10% $NaHCO_3$ (20 ml), then with brine (20 ml). The organic layer was separated, dried over anhydrous sodium sulfate, filtered and evaporated in vacuum. The residue was applied to column chromatography on silica gel. The column was washed with CH_2Cl_2, the product was eluted with system CH_2Cl_2/EtOH – 99/1 (%). The resulting 2-fluoro,N^6-((*R*)-α-methylbenzyl)-2′,3′,5′-tri-*O*-isobutyroyl adenosine was dissolved in 2M NH_3 in MeOH solution (1.77 ml, 3.5 mmol) at 0ºC and then left in a freezer at -7ºC for 3 days after which the mixture was evaporated and the residue was applied to column chromatography on silica gel. The column was washed using CH_2Cl_2/EtOH gradient, increasing in polarity from CH_2Cl_2/EtOH − 98/3 (%) to CH_2Cl_2/EtOH – 95/5 (%), the product was eluted with CH_2Cl_2/EtOH – 95/5 (%). The resulting product was dried in a vacuum desiccator over phosphorous pentaoxidefor 2 days to yield 25 mg (35% for two steps) as a white foam. R_f = 0.10 (CH_2Cl_2/EtOH – 95/5 (%)). ^{1}H-NMR (300.1 MHz, DMSO-d_6): δ =8.89 (d, 1H, J_{NH-CH} = 8.4 Hz,*N*6-H), 8.37 (s, 1H, H-8), 7.47-7.16 (m, 5H, H-Ph), 5.78 (d, 1H, $J_{1'2'}$ = 5.9 Hz, H-1′), 5.42 (d, 1H, $J_{2'-OH}$ = 6.1 Hz, 2′-OH), 5.41-5.31 (m, 1H, CH- overlapping with 2′-OH), 5.15 (d, 1H, $J_{3'-OH}$ = 4.9 Hz, 3′-OH), 5.01 (dd, 1H, $J_{5'a-OH}$ = 5.1 $J_{5'b-OH}$ = 6.1 Hz, 5′-OH), 4.51 (dd, 1H, $J_{2'1'}$ = 5.9 Hz, $J_{2'3'}$ = 4.7 Hz, H-2’), 4.12 (dd, 1H, $J_{3'2'}$ = 4.7 Hz, $J_{3'4'}$ = 3.8 Hz, H-3′), 3.93 (q, 1H, $J_{4'3'}$ = $J_{4'5'a}$= $J_{4'5'b}$ = 3.8 Hz, H-4′), 3.68 (ddd, 1H, $J_{5'a,5'b}$ = -12.0 Hz, $J_{5'a,4'}$ = 3.8 Hz,

$J_{5'a,OH}$ = 5.1 Hz, H5′a), 3.52 (ddd, 1H, $J_{5'b5'a}$ = -12.0 Hz, $J_{5'b4'}$ = 3.8 Hz, $J_{5'b\text{-}OH}$ = 6.1 Hz, H-5′b), 1.54 (d, 3H, $J_{CH\text{-}CH3}$ = 7.0 Hz, CH_3). ^{13}C-NMR (75.5 MHz, DMSO-d_6): δ = 158.41 (d, $^1J_{C\text{-}F}$= 204.3 Hz, C-2), 155.15 (d, $^3J_{C\text{-}F}$= 20.4 Hz, C-6), 149.93 (d, $^3J_{C\text{-}F}$= 20.3 Hz, C-4), 144.35 (C-1 Ph), 139.83 (C-8), 128.27 (Ph), 126.73 (Ph), 126.14 (Ph), 117.85 (C-5), 87.53 (C-1′), 85.62 (C-4′), 73.54 (C-2′), 70.33 (C-3′), 61.35 (C-5′), 49.28 (NCH-),22.08 (-CH_3). HRMS: *m/z* $[C_{18}H_{20}FN_5O_4+H]^+$ calculated 390.1572, found 390.1572, $[C_{18}H_{20}FN_5O_4+Na]^+$ calculated 412.1392, found 412.1383, $[C_{18}H_{20}FN_5O_4+K]^+$ calculated 428.1131, found 428.1125.

2-fluoro,*N*^{6}-((*S*)-α-methylbenzyl)adenosine (13b)

The procedure was analogous to preparation of 2-fluoro,*N*^{6}-((*R*)-α-methylbenzyl)adenosine (**13a**) using2-fluoro,*N*^{6}-((*S*)-α-methylbenzyl)adenine (**6b**) (42 mg, 0.163 mmol), 1,2,3,5-tetra-*O*-acetyl-*β*-D-ribofuranose (104 mg, 0.326 mmol), BSA (0.159 ml, 0.652 mmol) and TMSOTf (0.118 ml, 0.652 mmol) in anhydrous MeCN (5 ml) at 60°C in the N_2 atmosphere, followed by the deblocking in the presence of 2M NH_3 in MeOH solution (1.47 ml, 3 mmol) at -7ºC during 3 days. Further chromatographic purification on silica gel in CH_2Cl_2/EtOH – 95/5 (%) system gave **13b** as a white foam. Yield for two steps 25 mg (39%). R_f = 0.09 (CH_2Cl_2/EtOH – 95/5 (v/v)). ^{1}H-NMR (300.1 MHz, DMSO-d_6): δ = 8.91 (d, 1H, $J_{NH\text{-}CH}$ = 8.4 Hz, N6-H), 8.38 (s, 1H, H-8), 7.47-7.16 (m, 5H, H-Ph), 5.78 (d, 1H, $J_{1'2'}$ = 5.8 Hz, H-1′), 5.44 (d, 1H, $J_{2'\text{-}OH}$ = 6.1 Hz, 2′-OH), 5.41-5.31 (m, 1H, CH- overlapping with 2′-OH), 5.16 (d, 1H, $J_{3'\text{-}OH}$ = 5.0 Hz, 3′-OH), 5.02 (dd, 1H, $J_{5'a\text{-}OH}$ = 5.1, $J_{5'b\text{-}OH}$ = 6.2 Hz, 5′-OH), 4.49 (dd, 1H, $J_{2'1'}$ = 5.8 Hz, $J_{2'3'}$ = 4.7 Hz, H-2’), 4.12 (dd, 1H, $J_{3'2'}$ = 4.7 Hz, $J_{3'4'}$ = 3.8 Hz, H-3′), 3.92 (q, 1H, $J_{4'3'}$ = $J_{4'5'a}$= $J_{4'5'b}$ = 3.8 Hz, H-4′), 3.68 (ddd, 1H, $J_{5'a,5'b}$ = -12.0 Hz, $J_{5'a,4'}$ = 3.8 Hz, $J_{5'a,OH}$ = 5.1 Hz, H5′a), 3.54 (ddd, 1H, $J_{5'b5'a}$ = -12.0 Hz, $J_{5'b4'}$ = 3.8 Hz, $J_{5'b\text{-}OH}$ = 6.2 Hz, H-5′b), 1.54 (d, 3H, $J_{CH\text{-}CH3}$ = 7.0 Hz, CH_3). ^{13}C-NMR (75.5 MHz, DMSO-d_6): δ = 158.41 (d, ^1J= 204.5 Hz, C-2), 155.11 (d, ^3J= 20.4 Hz, C-6), 149.94 (d, ^3J= 20.3 Hz, C-4), 144.38 (C-1 Ph), 139.71 (C-8), 128.24 (Ph), 126.69 (Ph), 126.12 (Ph), 117.75 (C-5), 87.42 (C-1′), 85.56 (C-4′), 73.67 (C-2′), 70.27 (C-3′), 61.29 (C-5′), 49.25 (NCH-),22.06 (-CH_3). HRMS: *m/z* $[C_{18}H_{20}FN_5O_4+H]^+$ calculated 390.1572, found 390.1573, $[C_{18}H_{20}FN_5O_4+Na]^+$ calculated 412.1392, found 412.1393, $[C_{18}H_{20}FN_5O_4+K]^+$ calculated 428.1131, found 428.1133.

2-chloro,*N*^{6}-((*R*)-α-methylbenzyl)adenosine (14a)

The procedure was analogous to preparation of 2-fluoro,*N*^{6}-((*R*)-α-methylbenzyl)adenosine (**13a**) using2-chloro,*N*^{6}-((*R*)-α-methylbenzyl)adenine (**7a**)(80 mg, 0.29 mmol), 1,2,3,5-tetra-*O*-acetyl-*β*-D-ribofuranose (185 mg, 0.58 mmol), BSA (0.284 ml, 1.16 mmol) and TMSOTf (0.210 ml, 1.16 mmol) in anhydrous MeCN (5 ml) at 60°C in the N_2 atmosphere, followed by the deblocking in the presence of 2M NH_3 in MeOH solution (1.03 ml, 2.89 mmol) at -7ºC during 3 days. Further chromatographic purification on silica gel in CH_2Cl_2/EtOH – 95/5 (%) system gave **14a** as a white foam. Yield for two steps 38 mg (32 %). R_f = 0.12 (CH_2Cl_2/EtOH – 95/5 (%)). ^{1}H-NMR (300.1 MHz, DMSO-d_6): δ =8.81 (d, 1H, $J_{NH\text{-}CH}$ = 8.4

Hz,N6-H), 8.40 (s, 1H, H-8), 7.46-7.17 (m, 5H, H-Ph), 5.81 (d, 1H, $J_{1'2'}$ = 5.9 Hz, H-1′), 5.44 (d, 1H, $J_{2'-OH}$ = 6.1 Hz, 2′-OH), 5.43-5.35 (m, 1H, CH- overlapping with 2′-OH), 5.17 (d, 1H, $J_{3'-OH}$ = 5.0 Hz, 3′-OH), 5.02 (dd, 1H, $J_{5'a-OH}$ = 5.1, $J_{5'b-OH}$ = 6.2 Hz, 5′-OH), 4.49 (dd, 1H, $J_{2'1'}$ = 5.9 Hz, $J_{2'3'}$ = 4.7 Hz, H-2’), 4.12 (dd, 1H, $J_{3'2'}$ = 4.7 Hz, $J_{3'4'}$ = 3.8 Hz, H-3′), 3.93 (q, 1H, $J_{4'3'}$ = $J_{4'5'a}$= $J_{4'5'b}$ = 3.8 Hz, H-4′), 3.66 (ddd, 1H, $J_{5'a,5'b}$ = -12.0 Hz, $J_{5'a,4'}$ = 3.8 Hz, $J_{5'a,OH}$ = 5.1 Hz, H5′a), 3.52 (ddd, 1H, $J_{5'b5'a}$ = -12.0 Hz, $J_{5'b4'}$ = 3.8 Hz, $J_{5'b-OH}$ = 6.2 Hz, H-5′b), 1.54 (d, 3H, J_{CH-CH3} = 7.0 Hz, CH_3). ^{13}C-NMR (75.5 MHz, DMSO-d_6): δ = 154.18 (C-6), 152.99 (C-2), 149.72 (C-4), 144.30 (C-1 Ph), 139.93 (C-8), 128.24 (Ph), 126.71 (Ph), 126.18 (Ph), 118.50 (C-5), 87.44 (C-1′), 85.70 (C-4′), 73.60 (C-2′), 70.35 (C-3′), 61.34 (C-5′), 49.06 (NCH-),21.95 (-CH_3). HRMS: *m/z* $[C_{18}H_{20}ClN_5O_4+H]^+$ calculated 406.1277, found 406.1274, $[C_{18}H_{20}ClN_5O_4+Na]^+$ calculated 428.1096, found 428.1093, $[C_{18}H_{20}ClN_5O_4+K]^+$ calculated 444.0835, found 444.0832.

2-chloro,*N*^{6}-((*S*)-α-methylbenzyl)adenosine (14b)

The procedure was analogous to preparation of 2-fluoro,*N*^{6}-((*R*)-α-methylbenzyl)adenosine (**13a**) using2-chloro,*N*^{6}-((*S*)-α-methylbenzyl)adenine (**7b**) (90 mg, 0.33 mmol), 1,2,3,5-tetra-*O*-acetyl-*β*-D-ribofuranose (210 mg, 0.66 mmol), BSA (0.323 ml, 1.32 mmol) and TMSOTf (0.239 ml, 1.32 mmol) in anhydrous MeCN (5 ml) at 60°C in the N_2 atmosphere, followed by the deblocking in the presence of 2M NH_3 in MeOH solution (1.21 ml, 3.38 mmol) at -7ºC during 3 days. Further chromatographic purification on silica gel in CH_2Cl_2/EtOH – 95/5 (%) system gave **14b** as a white foam. Yield for two steps 45 mg (34%). R_f = 0.12 (CH_2Cl_2/EtOH – 95/5 (%)). ^{1}H-NMR (300.1 MHz, DMSO-d_6): δ =8.81 (d, 1H, J_{NH-CH} = 8.3 Hz, N^6-H), 8.41 s (1H, H-8), 7.46-7.18 (m, 5H, H-Ph), 5.81 (d, 1H, $J_{1'2'}$ = 5.9 Hz, H-1′), 5.43 (d, 1H, $J_{2'-OH}$ = 6.1 Hz, 2′-OH), 5.43-5.35 (m, 1H, CH- overlapping with 2′-OH), 5.16 (d, 1H, $J_{3'-OH}$ = 5.0 Hz, 3′-OH), 5.02 (dd, 1H, $J_{5'a-OH}$ = 5.1 Hz, $J_{5'b-OH}$ = 6.2 Hz, 5′-OH), 4.49 (dd, 1H, $J_{2'1'}$ = 5.9 Hz, $J_{2'3'}$ = 4.7 Hz, H-2’), 4.12 (dd, 1H, $J_{3'2'}$ = 4.7 Hz, $J_{3'4'}$ = 3.8 Hz, H-3′), 3.93 (q, 1H, $J_{4'3'}$ = $J_{4'5'a}$= $J_{4'5'b}$ = 3.8 Hz, H-4′), 3.65 (ddd, 1H, $J_{5'a,5'b}$ = -12.0 Hz, $J_{5'a,4'}$ = 3.8 Hz, $J_{5'a,OH}$ = 5.1 Hz, H5′a), 3.54 (ddd, 1H, $J_{5'b5'a}$ = -12.0 Hz, $J_{5'b4'}$ = 3.8 Hz, $J_{5'b-OH}$ = 6.2 Hz, H-5′b), 1.54 (d, 3H, J_{CH-CH3} = 7.0 Hz, CH_3). ^{13}C-NMR (75.5 MHz, DMSO-d_6): δ = 154.17 (C-6), 153.01 (C-2), 149.72 (C-4), 144.35 (C-1 Ph), 139.85 (C-8), 128.23 (Ph), 126.70 (Ph), 126.16 (Ph), 118.44 (C-5), 87.36 (C-1′), 85.67 (C-4′), 73.73 (C-2′), 70.32 (C-3′), 61.30 (C-5′), 49.06 (NCH-),21.94 (-CH_3). HRMS: *m/z* $[C_{18}H_{20}ClN_5O_4+H]^+$ calculated 406.1277, found 406.1275, $[C_{18}H_{20}ClN_5O_4+Na]^+$ calculated 428.1096, found 428.1092, $[C_{18}H_{20}ClN_5O_4+K]^+$ calculated 444.0835, found 444.0830.

2-amino,*N*^{6}-((*R*)-α-methylbenzyl)adenosine (16a)

Method A. A mixture of 1-(β-D-ribofuranosyl)-2-amino-6-chloropurine (**15**) (150 mg, 0.5 mmol), (*R*)-(+)-α-methylbenzylamine (0.26 ml, 2.05 mmol) and DIPEA (0.43 ml, 2.5 mmol) in dry DMF (5 ml) was heated at 80°C for 7.5 h. The reaction mixture was then evaporated to a syrup state, which was applied to

applied to chromatographic column with silica-gel (30 ml) for purification. The column was washed using CH_2Cl_2: EtOH gradient, increasing in polarity from CH_2Cl_2/EtOH – 98/2 (%)) to CH_2Cl_2/EtOH – 95/5 (%)). The product was eluted with CH_2Cl_2/EtOH – 90/10 (%). Fractions, containing the product, were collected and evaporated in vacuum to near dryness. The resulting syrup was crystallized in ether (3.5 ml). The crystals were filtered, washed with cold ether (2×1.5 ml) and dried on a vacuum pump for 3 hrs (bath temperature 50°C) and in vacuum desiccator over phosphorous pentoxide for 2 days to yield to yield 88 mg (46%) of pure product.

Method B. O^6-(Benzotriazol-1-yl)-2′,3′,5′-tri-O-isobutyroyl guanosine **(18)** was obtained according to a procedure analogous to the preparation of O^6-benzotriazolyl-2′,3′,5′-tri-*O*-isobutyroyl inosine **(10)** starting from 2′,3′,5′-tri-*O*-isobutyroyl guanosine **(17)** (105 mg, 0.213 mmol) in the presence of BOP (150 mg, 0.319 mmol) and DBU (0.08 ml, 0.532 mmol). Yield of **18** was 106 mg (81%) as a yellow foam. The foam should be kept at -25°C due to its rigid lability. R_f =0.70 (CH_2Cl_2/EtOH, 97.5/2.5 (%)). ^{1}H-NMR (300.1 MHz, $CDCl_3$): δ = 8.10 (d, 1H, ^3J= 8.3, benzotriazol), 7.89 (s, 1H, H-8), 7.57-7.40 (m, 3H, benzotriazol), 6.03 (d, 1H, $J_{1',2'}$= 5.1, H-1'), 5.96 (dd, 1H, $J_{1',2'}$= 5.1, $J_{2',3'}$= 5.2, H-2'), 5.73 (dd, 1H, $J_{3',2'}$= 5.2, $J_{3',4'}$ = 4.6, H-3'), 4.93 (br s, 2H, NH_2), 4.48-4.33 (m, 3H, H-4', H-5'a, H-5'b), 2.63-2.53 (m, 3H, C*H*-*i*Bu), 1.27-1.12 (m, 18H, CH_3-*i*Bu).

Further amination of nucleoside **18** (100 mg, 0.162 mmol) in the presence of (*R*)-(+)-α-methylbenzylamine (0.084 ml, 0.65 mmol) in anhydrous MeCNaccording to a procedure analogous to the preparation of **11a** led to 2-amino,N^6-((*R*)-α-methylbenzyl)-2′,3′,5′-tri-*O*-isobutroyl adenosine **(19)** as a yellow syrup. Yield 65 mg (67%). R_f = 0.45 (EtOAc/hexane – 1/1 (v/v)).^{1}H-NMR (300.1 MHz, $CDCl_3$): δ = 7.59 (s, 1H, H-8), 7.39 (dd, 2H, ^3J = 7.2, ^4J = 1.5, *ortho*-H, Ph), 7.32 (t, 2H, ^3J = 7.2, *meta*-H, Ph), 7.23 (tt, ^3J = 7.2, ^4J = 1.5, 1H, *para*-H, Ph), 5.99 (d, 1H, $J_{1'2'}$ = 5.3, H-1′), 5.92 (t, 1H, $J_{2'1'}$ =$J_{2'3'}$ = 5.3, H-2′), 5.80 – 5.70 (t, 1H, H-3′), 5.51 (br s, 1H, C*H*-CH_3), 4.71 (s, 2H, NH_2), 4.44 – 4.33 (m, 3H, H-4′, H-5′a, H-5′b), 2.68 – 2.47 (m, 3H, CH-*i*-Bu), 1.60 (d, ^3J = 6.9, 3H, CH_3 *i*-Bu), 1.24 – 1.11 (m, 18H, CH_3-*i*-Bu). ^{13}C-NMR (75.5 MHz, $CDCl_3$) δ = 176.79 (C=O), 175.75 (C=O), 175.52 (C=O), 160.16 (C-8), 154.54 (C-4), 144.09 (C-1 Ph), 135.47 (C-2), 128.64 (Ph), 127.21 (Ph), 126.30 (Ph), 86.08 (C-1′), 80.33 (C-4′), 72.91 (C-2′), 70.69 (C-3′), 63.33 (C-5′), 49.67 (*C*H-CH_3), 34.02, 33.94, 33.83 (*C*H-*i*-Bu), 22.64 (CH-*C*H_3), 19.10, 18.96, 18.82 (*C*H_3-*i*-Bu).

Then tri-O-isobutyroyl protected derivative **19** (62 mg, 0.104 mmol) was treated with 4M $MeNH_2$ in EtOH solution (3 ml) according to a procedure analogous to the preparation of N^6-((*R*)-α-methylbenzyl)-adenosine (**12a**). Yield of **(16a)** 31 mg (76%) as white powder. R_f =0.41 (CH_2Cl_2/EtOH − 95/5 (%)). M.p. 95-97°C (110°C decomposition with NH_3 emission). ^{1}H-NMR (300.1 MHz, CD_3OD): δ = 7.80 (s, 1H, H-8), 7.76 (s, 1H, N^6-H), 7.39 (dd, 2H, ^3J = 7.3, ^4J = 1.3 *ortho*-H-Ph), 7.30 (t, 2H, *J* = 7.3, *meta*-H, Ph), 7.20 (tt, 1H, ^3J = 7.3, ^4J = 1.3, *para*-H-Ph), 5.75 (d, 1H, $J_{1'2'}$ = 6.8, H-1′), 5.45 (br s, 1H, C*H*-CH_3), 5.42 (br s, 1H, NH_2), 4.73 (dd, 1H, $J_{2'1'}$ = 6.8, $J_{2'3'}$ = 5.1, H-2′), 4.28 (dd, 1H, $J_{3'2'}$ = 5.1, $J_{3'4'}$ = 1.8, H-3′), 4.16 (ddd,

1H, $J_{4'5'a}$ = 2.2, $J_{4'5'b}$ = 2.1, $J_{4'3'}$ = 1.8, H-4′), 3.89 (dd, 1H, $J_{5'a5'b}$ = - 12.6, $J_{5'a4'}$ = 2.2, H-5′a), 3.72 (dd, $J_{5'b5'a}$ = - 12.6, $J_{5'b4'}$ = 2.1, H-5′b), 1.58 (d, ^3J = 6.9, 3H, CH_3).^{13}C NMR is identical in all respects to **16b**. HRMS: *m/z* $[C_{18}H_{22}N_6O_4+H]^+$ calculated 387.1775, found 387.1768, $[C_{18}H_{22}N_6O_4+Na]^+$ calculated 409.1595, found 409.1600.

2-amino,N^6-((*S*)-α-methylbenzyl)adenosine (16b)

Method A.

A solution of 1-(β-D-ribofuranosyl)-2-amino-6-chloropurine (**15**) (144 mg, 0.48 mmol), (*S*)-(–)-α-methylbenzylamine (0.31 ml, 2.4 mmol) and DIPEA (0.51 ml, 3 mmol) in dry DMF (5 ml) was refluxed at 80°C for 7.5 h. The reaction mixture was then cooled to ambient temperature and evaporated to near dryness. The residue was then applied to chromatographic column with silica-gel for purification. The column was washed using CH_2Cl_2:EtOH gradient, increasing in polarity from CH_2Cl_2/EtOH – 98/2 (%)) to CH_2Cl_2/EtOH – 95/5 (%)). The product was eluted with CH_2Cl_2/EtOH – 90/10 (%). Fractions, containing the product, were collected and evaporated in vacuum to dryness. The residue was then precipitated from ether (3.5 ml) at -25°C, filtered through a Schott glass filter, washed with cold ether (2×1.5 ml), dried in vacuum desiccator over phosphorous pentoxide for 2 days to yield 64 mg (30%) as a yellow powder. R_f = 0.37 (CH_2Cl_2/EtOH – 95/5 (%)). M.p. 110°C (decomposition with NH_3 emission).^{1}H-NMR (300.1 MHz, DMSO-d_6): δ = 7.93 (s, 1H, H-8), 7.65 (br d, 1H, J_{NHCH} = 6.9,N^6-H), 7.43 (dd, 2H, ^3J = 8.5, ^4J = 1.4 *ortho*-H-Ph), 7.28 (dd, 2H, $^3J_1$ = 7.2, $^3J_2$ = 8.5, *meta*-H, Ph), 7.19 (tt, 1H, ^3J = 7.2, ^4J = 1.4, *para*-H-Ph), 5.73 (br s, 1H, NH_2, overlapping with H-1′), 5.72 (d, 1H, $J_{1'2'}$ = 6.2, H-1′), 5.51 (br s, 1H, C*H*-CH_3), 5.42 (br s, 1H, NH_2), 5.37 (dd, 1H, $J_{OH5'a}$ = 4.8, $J_{OH5'b}$ = 6.5, 5′-OH), 5.31 (d, 1H, $J_{2'OH}$ = 5.1, 2′-OH), 5.06 (br d, 1H, $J_{3'OH}$ = 4.4, 3′-OH), 4.48 (ddd, 1H, $J_{2'1'}$ = 6.2, $J_{2'3'}$ = 5.8, $J_{3'OH}$ = 5.1, H-2′), 4.09 (ddd, 1H, $J_{3'2'}$ = 5.8, $J_{3'4'}$ = 2.0, $J_{3'OH}$ = 4.4, H-3′), 3.89 (ddd, 1H, $J_{4'3'}$ = 2.0, $J_{4'5'a}$ = 2.9, $J_{4'5'b}$ = 3.9, H-4′), 3.63 (dd, 1H, $J_{5'a5'b}$ = - 12.1, $J_{5'a4'}$ = 2.9, $J_{5'aOH}$ = 4.8, H-5′a), 3.52 (ddd, $J_{5'b5'a}$ = - 12.1, $J_{5'bOH}$ = 6.5, $J_{5'b4'}$ = 3.9, H-5′b), 1.50 (d, ^3J = 7.0, 3H, CH_3). ^{13}C-NMR (75.5 MHz, DMSO-d_6): δ = 161.50 (C-6), 155.82 (C-2), 145.58 (C-4), 138.60 (*C*-1 Ph), 130.61 (C-8), 129.48 (Ph), 127.97 (Ph), 127.15 (Ph), 115.51 (C-5), 91.24 (C-1′), 88.20 (C-4′), 74.84 (C-2′), 72.96 (C-3′), 63.73 (C-5′), 50.60 (NCH-),22.99 (-CH_3). HRMS: *m/z* $[C_{18}H_{22}N_6O_4+H]^+$ calculated 387.1775, found 387.1771, $[C_{18}H_{22}N_6O_4+Na]^+$ calculated 409.1595, found 409.1589, $[C_{18}H_{22}N_6O_4+K]^+$ calculated 425.1334, found 425.1332.

1.4. Synthesis of *O*-isobutyroyl protected nucleoside synthons

2′,3′,5′-tri-*O*-isobutyroylguanosine (17) and N^2-isobutyroyl-2′,3′,5′-tri-*O*-isobutyroylguanosine (20)

A suspension of guanosine (1 g, 3.53 mmol) in pyridine (10 ml) and isobutyric anhydride (5.2 ml, 32 mmol) was heated at 80°C for 6 hrs until dissolution of guanosine. The reaction mixture was then treated with EtOH (5 ml) at 40°C to neutralize an excess of anhydride and evaporated in vacuum to a volume *ca*. 2.5 ml. The resulting mixture was quenched with CH_2Cl_2 (30 ml), washed successively with 10% aqueous

$NaHCO_3$ (50 ml) and H_2O (2×50 ml), dried over anhydrous Na_2SO_4, filtered and evaporated in vacuum to near dryness. The residue was co-evaporated with toluene (2×5 ml) and applied to chromatographic column with silica-gel for purification. The column was washed with CH_2Cl_2. N^2-isobutyroyl-2′,3′,5′-tri-*O*-isobutyroylguanosine was eluted with CH_2Cl_2/EtOH – 97/3 (%), 2′,3′,5′-tri-*O*-isobutyroylguanosine was eluted with CH_2Cl_2/EtOH in EtOH gradient from 5 to 10%. Fractions, containing the products, were collected, evaporated and dried on a vacuum pump for 1 h to obtain N^2-isobutyroyl-2′,3′,5′-tri-*O*-isobutyroylguanosine as a yellow slowly solidifying syrup (614 mg). R_f =0.2 (CH_2Cl_2/EtOH, 98/2 (%))^{1}H-NMR (300.1 MHz, $CDCl_3$): δ = 12.01 (s, 1H, NH), 8.96 (s, 1H, H-8), 7.69 (s, 2H, NH_2), 5.98 (dd, 1H, $J_{1',2'}$= 5.1, $J_{2',3'}$= 5.0, H-2'), 5.91 (d, 1H, $J_{1',2'}$= 5.1, H-1'), 5.81 (t, 1H, $J_{2',3'}$= 5.0, $J_{3',4'}$ = 3.9, H-3'), 4.63 (dd, 1H, $J_{5'a,5'b}$= -14.1, $J_{5'a,4'}$= 7.2, H-5'a), 4.50 (dd, 1H, $J_{5'a,5'b}$= -14.1, $J_{5'a,4'}$= 6.4, H-5'b), 4.53-4.48 (m, 1H, overlapping with H-5'b), 2.72 (sept, 1H, ^3J= 6.9, C*H*-N^2-*i*Bu), 2.66-2.48 (m, 3H, C*H*-*O*-*i*Bu), 1.32 (d, 3H, ^3J= 6.9, CH_3- N^2-*i*Bu), 1.30 (d, 3H, ^3J= 6.9, CH_3-N^2-*i*Bu), 1.22 (d, 6H, ^3J= 7.0, CH_3-*O*-*i*Bu), 1.16 (d, 3H, ^3J= 6.5, CH_3-*O*-*i*Bu), 1.14 (d, 3H, ^3J= 6.9, CH_3-*O*-*i*Bu), 1.12 (d, 3H, ^3J= 7.0, CH_3-*O*-*i*Bu) and 2′,3′,5′-tri-*O*-isobutyroylguanosine (180 mg) as slightly brown crystals. R_f =0.12 (CH_2Cl_2/EtOH, 98/2 (%)).^{1}H-NMR (300.1 MHz, $CDCl_3$): δ = 7.69 (s, 1H, H-8), 6.16 (s, 2H, NH_2), 5.98 (d, 1H, $J_{1',2'}$= 5.4, H-1'), 5.94 (t, 1H, $J_{2',3'}$= $J_{3',4'}$ = 5.4, H-2'), 5.72-5.66 (m, 1H, H-3'), 4.46-4.34 (m, 3H, H-4', H-5'a, H-5'b), 2.67-2.48 (m, 3H, C*H*-*i*Bu), 1.22-1.10 (m, 18H, CH_3-*i*Bu).

2-Isobutyroylamino,N^6-((*R*)-α-methylbenzyl)-2′,3′,5′-tri-*O*-isobutyroyladenosine (22a)

O^6-(Benzotriazol-1-yl)-N^2-isobutyroyl -2′,3′,5′-tri-*O*-isobutyroyl guanosine **(21)** was obtained according to a procedure analogous to the preparation of O^6-benzotriazolyl-2′,3′,5′-tri-*O*-isobutyroyl inosine **(10)** starting from N^2-isobutyroyl-2′,3′,5′-tri-*O*-isobutyroylguanosine (**20**) (500 mg, 1 mmol), BOP (660 mg, 1.5 mmol) and DBU (0.23 ml, 1.58 mmol). Yield 698 mg (96%) as a yellow foam. The foam should be kept at -25°C due to its rigid lability. R_f =0.44 (CH_2Cl_2/EtOH, 97/3 (%)). ^{1}H-NMR (300.1 MHz, DMSO-d_6): δ = 10.46 (s, 1H, NH), 8.60 (s, 1H, H-8), 8.20 (d, 1H, ^3J= 8.4, benzotriazol), 7.84 (d, 1H, ^3J= 8.4, benzotriazol), 7.66 (t, 1H, ^3J= 8.4, benzotriazol), 7.56 (t, 1H, ^3J= 8.4, benzotriazol), 6.26 (d, 1H, $J_{1',2'}$= 3.5, H-1'), 5.98-5.89 (m, 2H, H-2', H-3'), 4.43 (dd, 1H, $J_{5'a,5'b}$= -14.5, $J_{5'a,5'b}$= 4.3, H-5′a), 4.39-4.28 (m, 2H, H-4', H-5'b), 2.68-2.44 (m, 3H, C*H*-*i*Bu, overlapping with solvent residual peak), 1.14 (d, 9H, ^3J = 6.9, CH_3-*i*Bu), 1.10-1.00 (m, 15H, CH_3-*i*Bu).

A mixture of **21** (250 mg, 0.4 mmol) and (*R*)-(+)-α-methylbenzylamine (0.2 ml, 1.6 mmol) in dry acetonitrile (6 ml) was kept for 20 h at ambient temperature. The resulting mixture was then filtered from benzotriazole, evaporated in vacuum to a syrup state, which was applied to chromatographic column with silica-gel (30 ml) for purification. The product was eluted with EtOAc/hexane – 1/1 *(v/v)* (250 ml). Fractions, containing the product, were collected and evaporated in vacuum to dryness. The residue was dried on a vacuum pump for 1 h (bath temperature 50°C) to yield to yield 131 mg (49%) of pure product

22a as a syrup. R_f = 0.39 (EtOAc/hexane – 1:1 (v/v)).^{1}H-NMR (300.1 MHz, $CDCl_3$): δ = 7.80 (s, 1H, H-8), 7.78 (br s, 1H, N6-H), 7.42 (dd, 2H, ^3J = 8.7, ^4J = 1.5 *ortho*-H-Ph), 7.32 (br t, 2H, $^3J_1$ = 7.4, *meta*-H, Ph), 7.29-7.20 (m, 1H, *para*-H-Ph), 6.32 (br d, 1H, ^3J = 7.2, N*H*-CH), 6.05 (d, 1H, $J_{1'2'}$ = 4.8, H-1′), 5.83 (dd, 1H, $J_{2'3'}$ = 5.4, $J_{2'1'}$ = 4.8, H-2′), 5.78-5.69 (m, 1H, H-3′), 5.44 (br s, 1H, C*H*-CH_3), 4.48-4.42 (m, 2H, H-4′, H-5′a), 4.39 (dd, 1H, $J_{5'b5'a}$ = - 11.7, $J_{5'b4'}$ = 5.5, H-5′b), 2.67-2.50 (m, 4H, C*H*-*i*-Bu), 1.64 (d, ^3J = 6.9, 3H, CH_3), 1.28-1.10 (m, 32H, CH_3-*i*-Bu). ^{13}C-NMR (75.5 MHz, $CDCl_3$): δ = 177.35, 176.73, 175.70, 175.53 (C=O), 154.31 (C-6), 153.21 (C-2), 143.74 (C-4), 137.41 (*C*-1 Ph), 128.72 (Ph), 127.35 (Ph), 126.17 (Ph), 116.84 (C-5), 86.47 (C-1′), 80.35 (C-4′), 73.42 (C-2′), 70.61 (C-3′), 63.24 (C-5′), 50.29 (NCH-), 34.04, 33.92, 33.82 (*C*H-*i*-Bu), 29.80 (*C*H_3-N^2-*i*-Bu),22.80 (-CH_3), 19.45, 19.18, 19.09, 19.01, 18.98, 18.94, 18.78 (*C*H_3-*i*-Bu).

2-Isobutyroylamino,N^6-((*S*)-α-methylbenzyl)-2′,3′,5′-tri-*O*-isobutyroyladenosine (22b)

The procedure was analogous to the preparation of 2-isobutyroylamino,N^6-((*R*)-α-methylbenzyl)-2′,3′,5′-tri-*O*-isobutyroyladenosine (**22a**) starting from O^6-(benzotriazol-1-yl)-N^2-isobutyroyl-2′,3′,5′-tri-*O*-isobutyroyl guanosine (200 mg, 0.32 mmol) (**21**) and (*S*)-(–)-α-methylbenzylamine (0.165 ml, 1.28 mmol). Yield of **22b** was 102 mg (47%) as a syrup. R_f = 0.39 (EtOAc/hexane – 1:1 (v/v)).^{1}H-NMR (300.1 MHz, $CDCl_3$): δ = 7.78 (s, 1H, H-8), 7.75 (br s, 1H, N^6H), 7.40 (dd, 2H, ^3J = 8.7, ^4J = 1.5 *ortho*-H-Ph), 7.33 (br t, 2H, $^3J_1$ = 7.4, *meta*-H, Ph), 7.29-7.20 (m, 1H, *para*-H-Ph), 6.32 (br d, 1H, ^3J = 7.2, N*H*-CH), 6.04 (d, 1H, $J_{1'2'}$ = 4.7, H-1′), 5.83 (dd, 1H, $J_{2'3'}$ = 5.8, $J_{2'1'}$ = 4.7, H-2′), 5.78-5.69 (m, 1H, H-3′), 5.44 (br s, 1H, C*H*-CH_3), 4.48-4.32 (m, 3H, H-4′, H-5′a, H-5′b), 2.67-2.47 (m, 4H, C*H*-*i*-Bu), 1.64 (d, ^3J = 6.9, 3H, CH_3), 1.30-1.10 (m, 32H, CH_3-*i*-Bu). ^{13}C-NMR (75.5 MHz, $CDCl_3$): δ = 177.35, 176.73, 175.70, 175.53 (C=O), 154.31 (C-6), 153.21 (C-2), 143.74 (C-4), 137.41 (*C*-1 Ph), 128.72 (Ph), 127.35 (Ph), 126.17 (Ph), 116.84 (C-5), 86.47 (C-1′), 80.35 (C-4′), 73.42 (C-2′), 70.61 (C-3′), 63.24 (C-5′), 50.29 (NCH-), 34.04, 33.92, 33.82 (*C*H-*i*-Bu), 29.80 (*C*H_3-N^2-*i*-Bu),22.80 (-CH_3), 19.45, 19.18, 19.09, 19.01, 18.98, 18.94, 18.78 (*C*H_3-*i*-Bu).

2-Isobutyroylamino,N^6-((*R*)-α-methylbenzyl)adenosine (23a)

A solution of 2-isobutyroylamino,N^6-((*R*)-α-methylbenzyl)-2′,3′,5′-tri-*O*-isobutyroyladenosine (**22a**) (131 mg, 0.214 mmol) in 5.2 ml of 4M $MeNH_2$/EtOH was kept for 4 days at ambient temperature. The resulting mixture was then evaporated in vacuum. The residue was dissolved in dichloromethane and applied to chromatographic column with silica-gel (7 ml) for purification. The column was washed with CH_2Cl_2, increasing in polarity to CH_2Cl_2/EtOH – 95/5 (%)). The product was eluted with CH_2Cl_2/EtOH – 90/10 (%). Fractions, containing the product, were collected and evaporated in vacuum to dryness. The residue was dried on a vacuum pump for 1 h (bath temperature 50°C) to yield to yield 39 mg (40%) of pure product as a foam. R_f =0.57 (CH_2Cl_2/EtOH – 95/5 (%)) ^{1}H-NMR (300.1 MHz, DMSO-d_6): δ = 9.76 (br s, 1H, N2-H), 8.22 (br s, 2H, N^{6-}H, H-8), 7.47 (dd, 2H, ^3J = 7.2, ^4J = 1.2, *ortho*-H-Ph), 7.29 (t, 2H, ^3J=

7.2, *meta*-H, Ph), 7.19 (tt, 1H, ^3J = 7.2, ^4J = 1.2 *para*-H-Ph), 5.80 (d, 1H, $J_{1'2'}$ = 6.1, H-1′), 5.56 (br s, C*H*-CH_3), 5.47 (d, 1H, $J_{2'OH}$ = 5.9, 2′-OH), 5.10 (d, 1H, $J_{3'OH}$ = 4.6, H-3′), 4.95 (t, 1H, $J_{5'OH}$ = 5.9, 5′-OH), 4.54 (ddd, 1H, $J_{2'1'}$ = 6.1, $J_{2'OH}$ = 5.9, $J_{2'3'}$ = 5.0, H-2′), 4.14 (ddd, 1H, $J_{3'2'}$ = 5.0, $J_{3'OH}$ = 4.6, $J_{3'4'}$ = 2.8, H-3′), 3.90 (ddd, 1H, $J_{4'3'}$ = 2.8, $J_{4'5'a}$ = 3.5, $J_{4'5'b}$ = 4.5, H-4′), 3.61 (ddd, 1H, $J_{5'a5'b}$ = -11.9, $J_{5'OH}$ =5.9, $J_{5'a4'}$ = 3.5, H-5′a), 3.52 (ddd, 1H, $J_{5'b5'a}$ = -11.9, $J_{5'OH}$ = 5.9, $J_{5'b4'}$ = 4.5, H-5′b), 2.94 (m, 1H, C*H*-*i*-Bu), 1.53 (d, 3H, ^3J = 7.0, CH_3), 1.06 (d, 3H, ^3J = 6.8, CH_3-*i*-Bu), 1.01 (d, 3H, ^3J = 6.7, CH_3-*i*-Bu).

1.5. Ado^{BOM} and Ade^{BOM}

N^6-(benzyloxymethyl)adenosine (Ado^{BOM})

To the solution of *N*^{6}-acetyl-2′,3′,5′-tri-*O*-acetyladenosine (**24**) (400 mg, 0.915 mmol) in dry MeCN (5 ml) benzyloxymethyl chloride (0.252 ml, 1.83 mmol) and DBU (0.273 ml, 1.83 mmol) were added. The solution was kept at ambient temperature for 72 h. The reaction was monitored by TLC (silica gel, CH_2Cl_2/EtOH – 97/3 (%)). Then, the reaction mixture was evaporated in vacuum and the residue was diluted with methylene chloride (30 ml) and washed with water (2×15 ml). The organic layer was applied to column chromatography. The column was washed with CH_2Cl_2, product was eluted with (CH_2Cl_2/EtOH − 97/3 (%)). Resulting *N*^{6}-(benzyloxymethyl)-2′,3′,5′-tri-*O*-acetyladenosine was dissolved in 5M $PrNH_2$ in MeOH solution (9.1 ml, 45.75 mmol) and was left for 24 h, after which the mixture was evaporated and the residue was applied to column chromatography. The column was washed with (CH_2Cl_2/EtOH − 97/3 (%)), the product was eluted with (CH_2Cl_2/EtOH − 90/10 (%)) to give **Ado^{BOM}**as a white foam. Yield 193 mg (54%). R_f =0.23 (CH_2Cl_2/EtOH − 95/5 (%)). ^{1}H-NMR (300.1 MHz, DMSO-d_6): δ =8.72 (br s, 1H, N6-H), 8.45 (s, 1H, H-8), 8.31 (s, 1H, H-2), 7.34-7.22 (m, 5H, H-Ph), 5.92 (d, 1H, $J_{1'2'}$ = 6.0 Hz, H-1′), 5.43 (d, 1H, $J_{2'-OH}$ = 6.2 Hz, 2′-OH), 5.28 (dd, 1H, $J_{5'a-OH}$ = 4.5, $J_{5'b-OH}$ = 6.8 Hz, 5′-OH), 5.16 (d, 1H, $J_{3'-OH}$ = 4.8 Hz, 3′-OH), 5.12 (br s, 2H, N^6-CH_2), 4.61 (ddd, 1H, $J_{2'1'}$ = 6.0 Hz, $J_{2'3'}$ = 5.0 Hz, $J_{2'OH}$ = 6.2 Hz, H-2’), 4.58 (s, 2H, CH_2-Ph), 4.17 (ddd, 1H, $J_{3'2'}$ = 5.0 Hz, $J_{3'4'}$ = 3.5 Hz, $J_{3'OH}$ = 4.8 Hz, H-3′), 3.97 (q, 1H, $J_{4'3'}$ = $J_{4'5'a}$= $J_{4'5'b}$ = 3.5 Hz, H-4′), 3.69 (ddd, 1H, $J_{5'a,5'b}$ = -12.0 Hz, $J_{5'a,4'}$ = 3.5 Hz, $J_{5'a,OH}$ = 4.5 Hz, H5′a), 3.56 (ddd, 1H, $J_{5'b5'a}$ = -12.0 Hz, $J_{5'b4'}$ = 3.5 Hz, $J_{5'b-OH}$ = 6.8 Hz, H-5′b).^{13}C-NMR (75.5 MHz, DMSO-d_6): δ = 154.45 (C-6), 152.12 (C-2), 149.33 (C-4), 140.45 (C-8), 138.56 (C-1 Ph), 128.08 (Ph), 127.41 (Ph), 127.19 (Ph), 119.77 (C-5), 87.84 (C-1′), 85.79 (C-4′), 73.54 (C-2′), 70.52 (C-3′), 70.24 (NCH_2-), 68.90 (CH_2-Ph), 61.54 (C-5′). HRMS: *m/z* $[C_{18}H_{21}N_5O_5+H]^+$ calculated 388.1615, found 388.1608, $[C_{18}H_{21}N_5O_5+Na]^+$ calculated 410.1435, found 410.1420, $[C_{18}H_{21}N_5O_5+K]^+$ calculated 426.1174, found 426.1144.

N^6-(benzyloxymethyl)adenine (Ade^{BOM})

To a mixture of *N*^{6}-(benzyloxymethyl)adenosine (**Ado^{BOM}**) (193 mg, 0.498 mmol) and KH_2AsO_4 (90 mg, 0.498 mmol) in 50 mM Tris-HCl buffer pH 7.5 (10 ml), PNP (0.143 ml, 5 activity units) was added and

the mixture was kept at 50°C for 24 h. During the reaction, the initial nucleoside dissolved and the product crystallized. The precipitate was filtered, washed with H_2O (5 × 5 ml) and dried in a vacuum desiccator over P_2O_5 to give N^6-(benzyloxymethyl)adenine (**Ade^{BOM}**) (59 mg, 46%) as white powder. R_f =0.33 (CH_2Cl_2/EtOH − 95/5 (%)). M.p. 315 °C (decomposition). ^{1}H-NMR (300.1 MHz, DMSO-d_6): δ = 13.02 (br s, 1H, *N*9-H), 8.52 (br s 1H, N^6-H), 8.27 (s, 1H, H-8), 8.17 (s, 1H, H-2), 7.45-7.15 (m, 5H, H-Ph), 5.14 (br s, 2H, CH_2-BOM), 4.58 (s, 2H, CH_2-BOM). ^{13}C-NMR (75.5 MHz, DMSO-d_6): δ = 154.13 (C-6), 152.10 (C-2), 150.55 (C-4), 139.58 (C-8), 138.65 (C-1 Ph), 128.09 (Ph), 127.41 (Ph), 127.18 (Ph), 118.85 (C-5), 70.53 (NCH_2-), 68.83 (CH_2-Ph). HRMS: *m/z* $[C_{13}H_{13}N_5O+H]^+$ calculated 256.1191, found 256.1193, $[C_{13}H_{13}N_5O+Na]^+$ calculated 278.1012, found 278.1006, $[C_{13}H_{13}N_5O\ +K]^+$ calculated 294.0752, found 294.0743.

2. CD-spectra of enantiomers

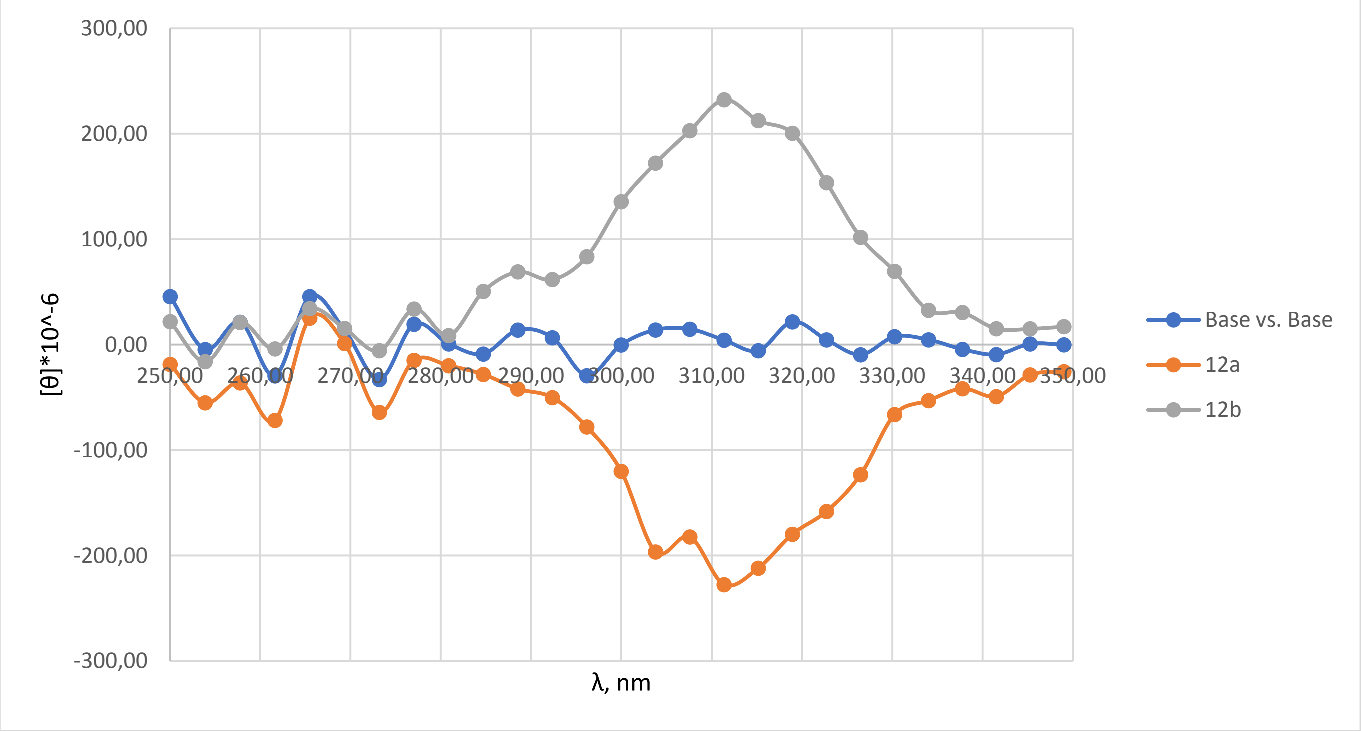

Figure S1. CD-spectra of enantiomers **12a** and **12b**.

In the absorption region of the chromophore group (311 nm), a positive Cotton effect was observed for the *S*-isomer (double refraction of waves with elliptical polarization), and a negative Cotton effect for the *R*-isomer, which indicated the manifestation of different optical properties by compounds **12a** and **12b**.

3. NMR-spectroscopy

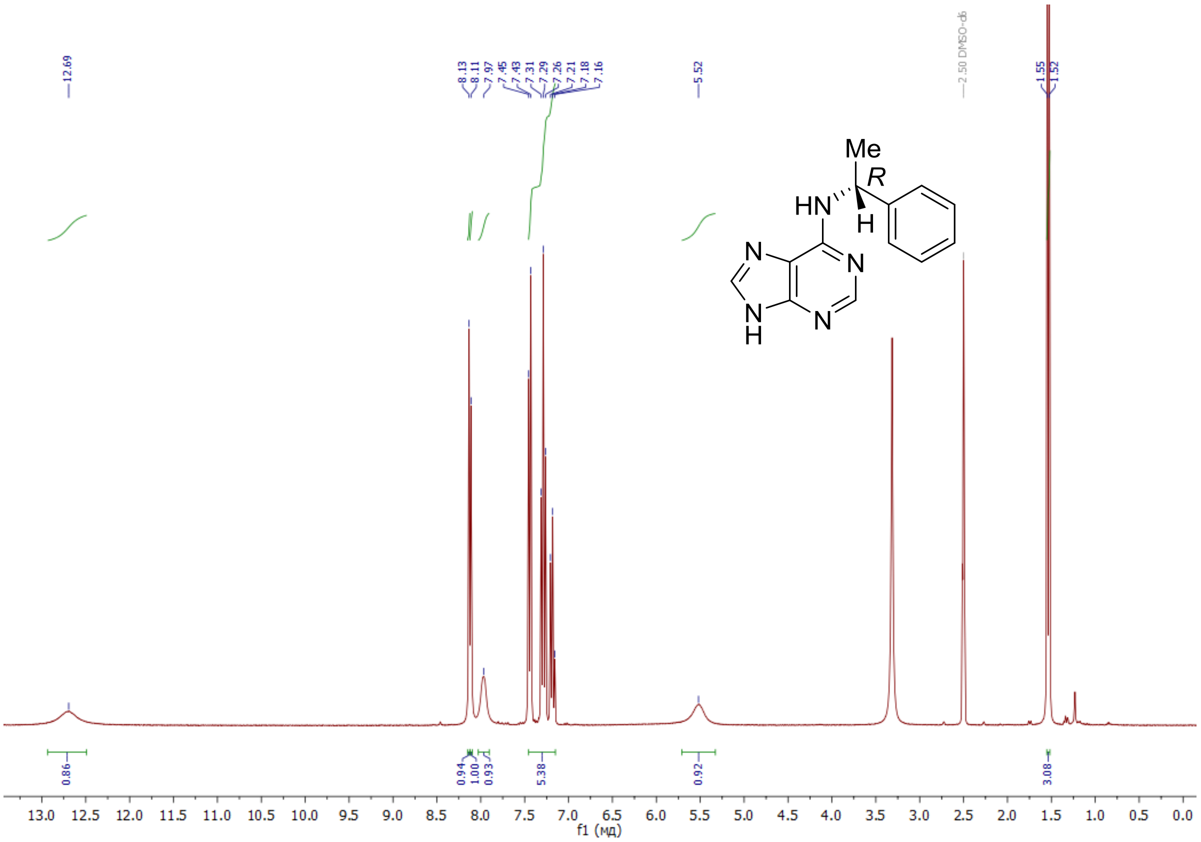

^{1}H-NMR-spectrum (300.1 MHz) of N^{6}-((R)-α-methylbenzyl)adenine (**5a**) in DMSO-d^{6} at 303K

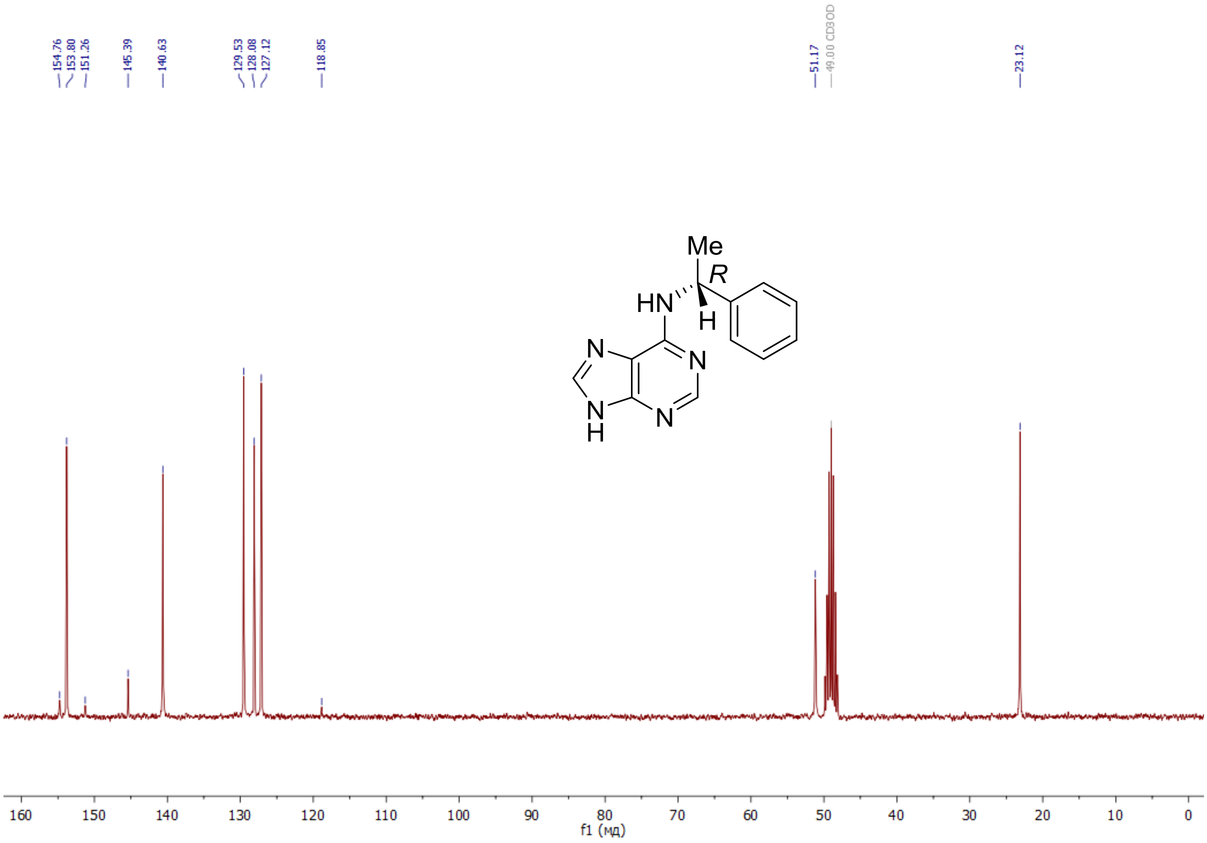

^{13}C-NMR-spectrum (75.5 MHz) of N^{6}-((R)-1-α-methylbenzyl)adenine (**5a**) in CD_3OD at 303K

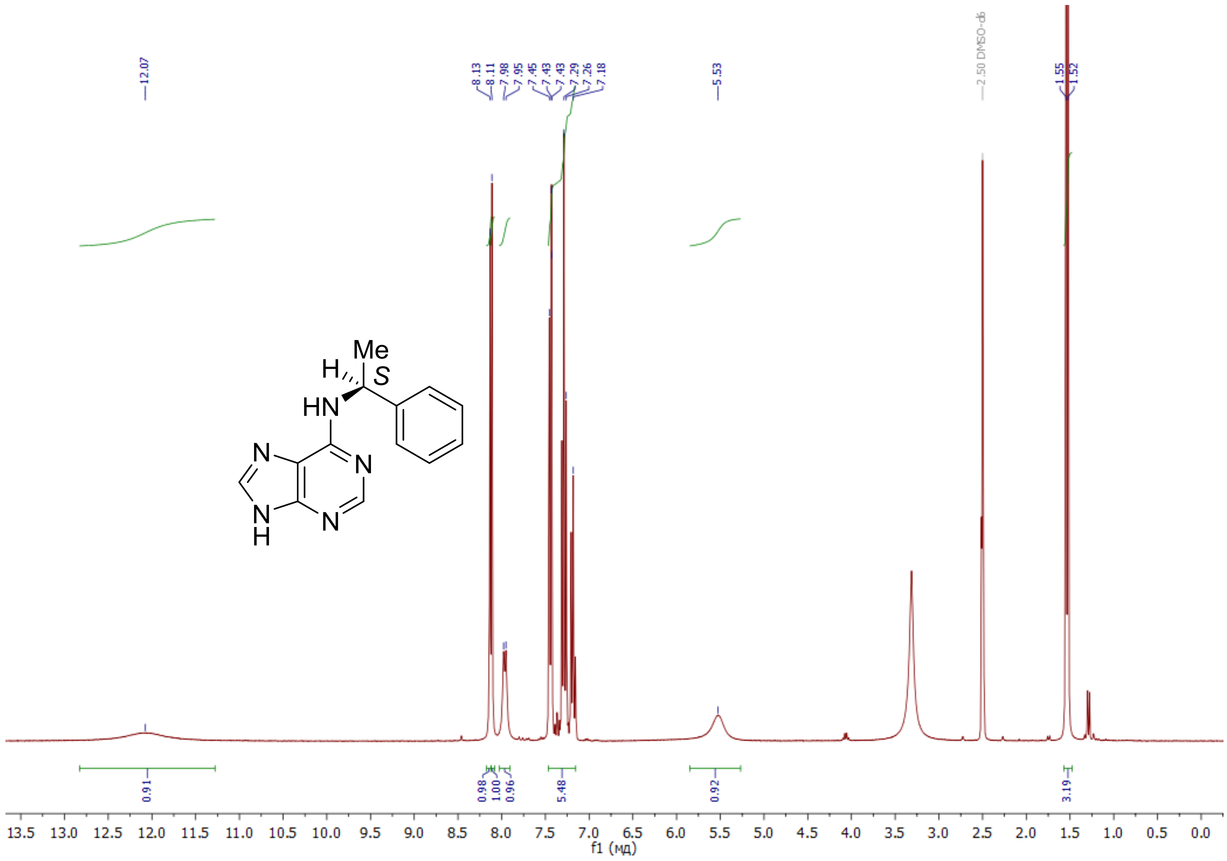

^{1}H-NMR-spectrum (300.1 MHz) of N^6-((S)-α-methylbenzyl)adenine (**5b**) in DMSO-d^6 at 303K

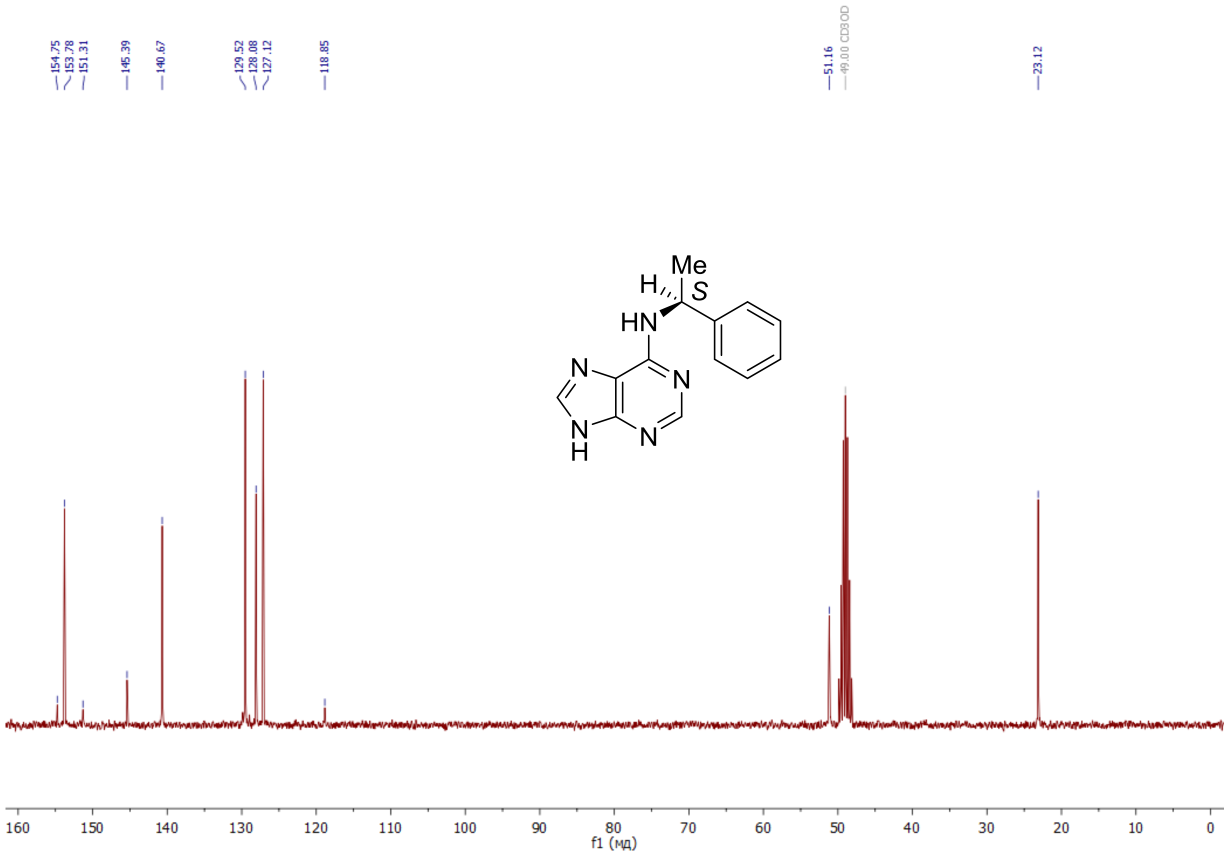

^{13}C-NMR-spectrum (75.5 MHz) of N^6-((S)-α-methylbenzyl)adenine (**5b**) in CD_3OD at 303K

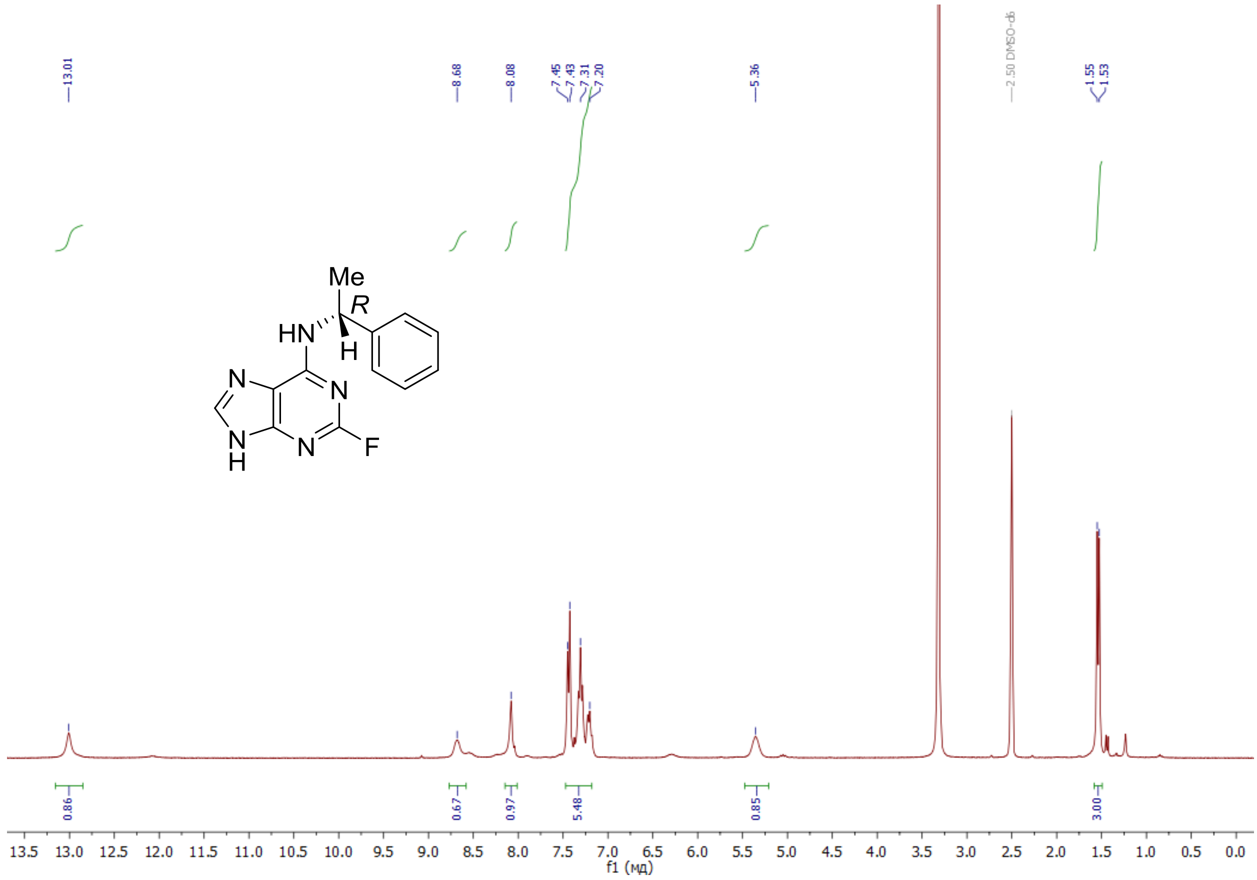

1H-NMR-spectrum (300.1 MHz) of 2-fluoro,N^6-((R)-α-methylbenzyl)adenine (**6a**) in DMSO-d^6 at 303K

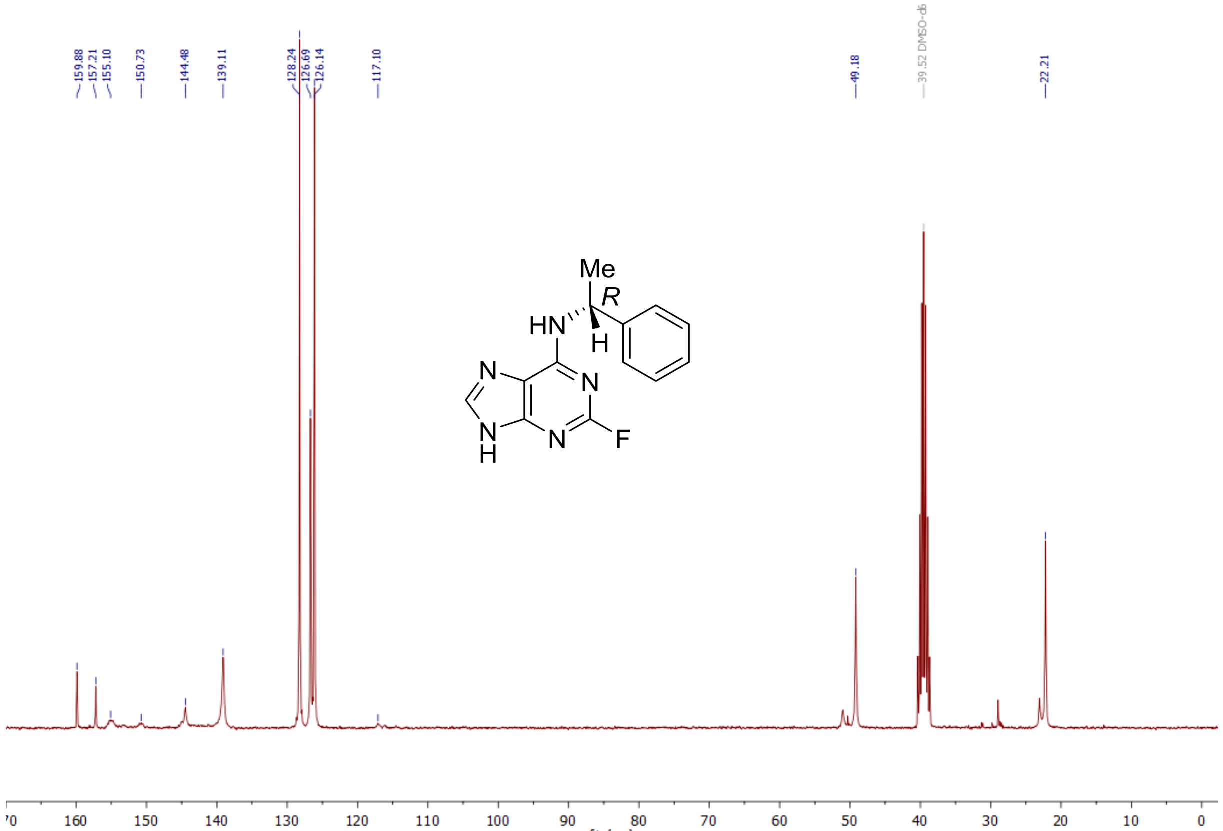

^{13}C-NMR-spectrum (75.5 MHz) of 2-fluoro,N^6-((R)-α-methylbenzyl)adenine (**6a**) in DMSO-d^6 at 303K

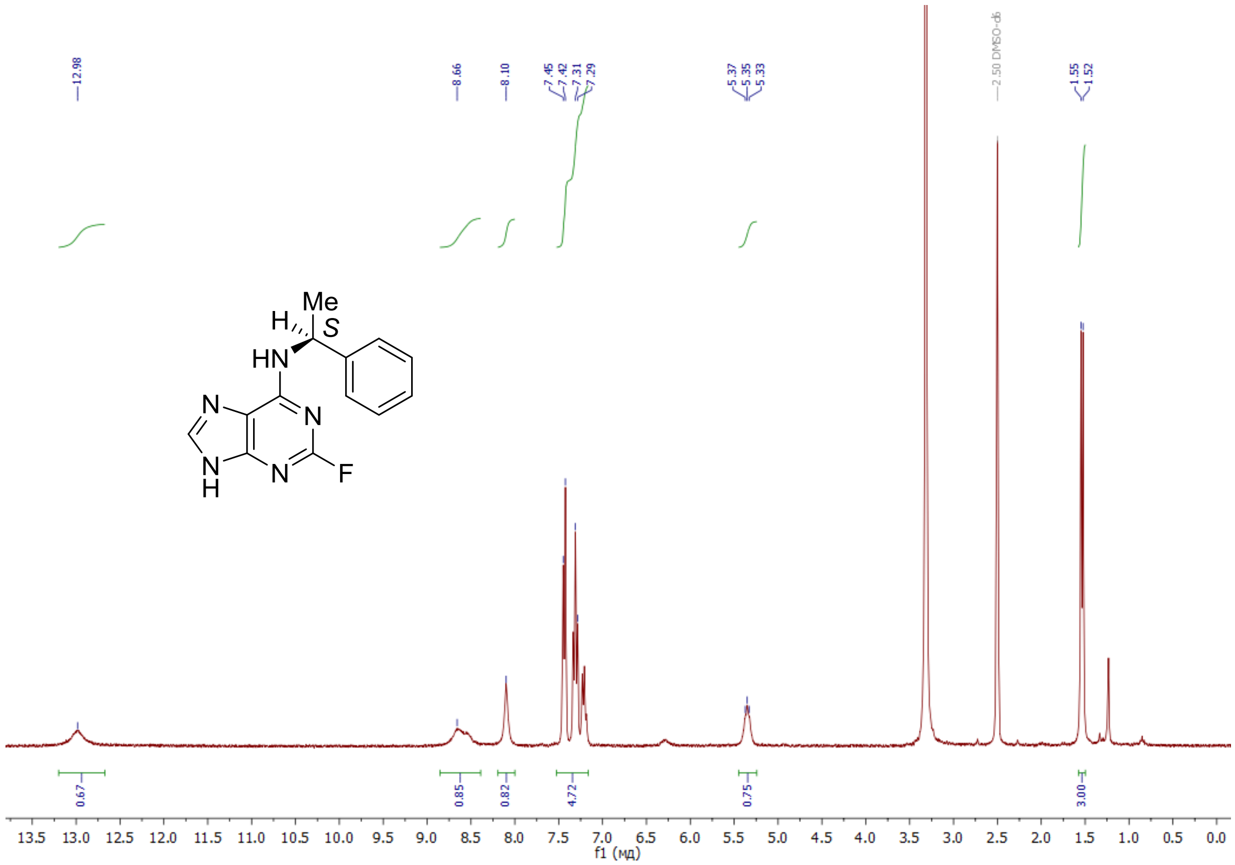

1H-NMR-spectrum (300.1 MHz) of 2-fluoro, *N*^{6}-((S)-α-methylbenzyl)adenine (**6b**) in DMSO-d^6 at 303K

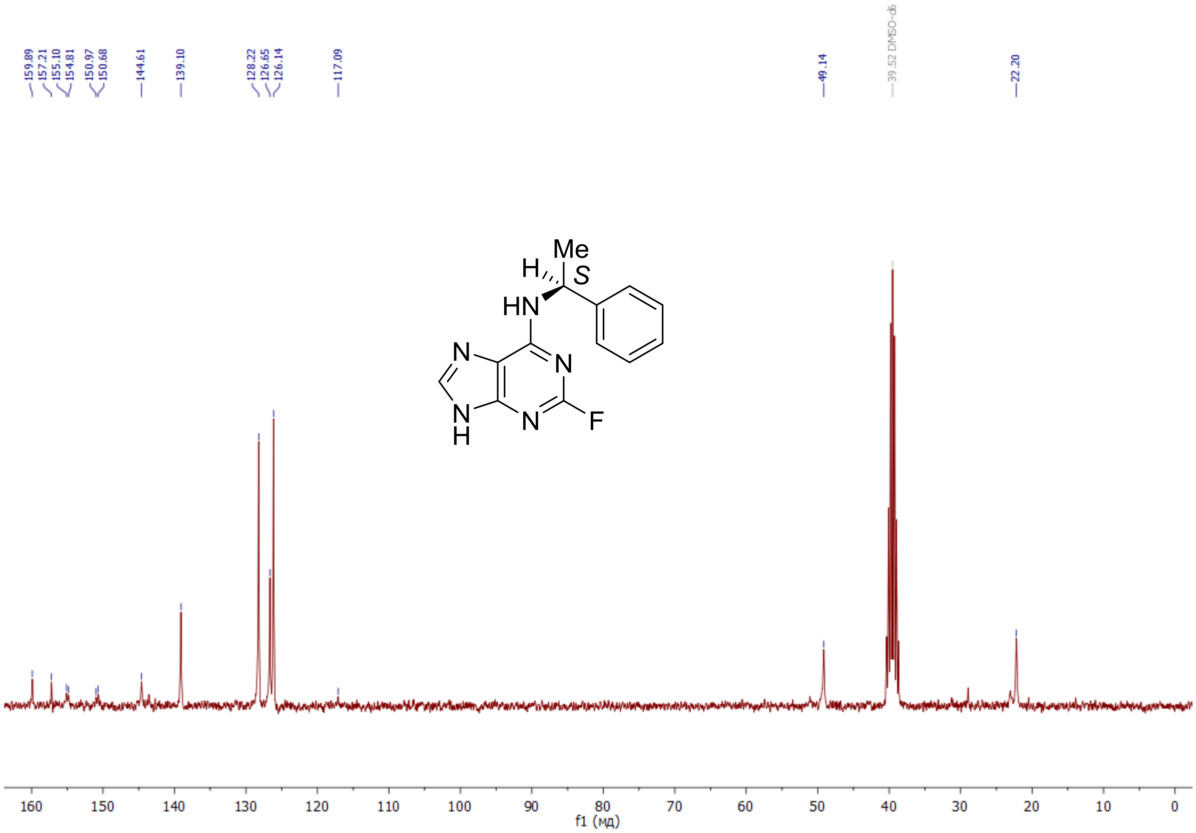

^{13}C-NMR-spectrum (75.5 MHz) of 2-fluoro, *N*^{6}-((S)-α-methylbenzyl)adenine (**6b**) in DMSO-d^6 at 303K

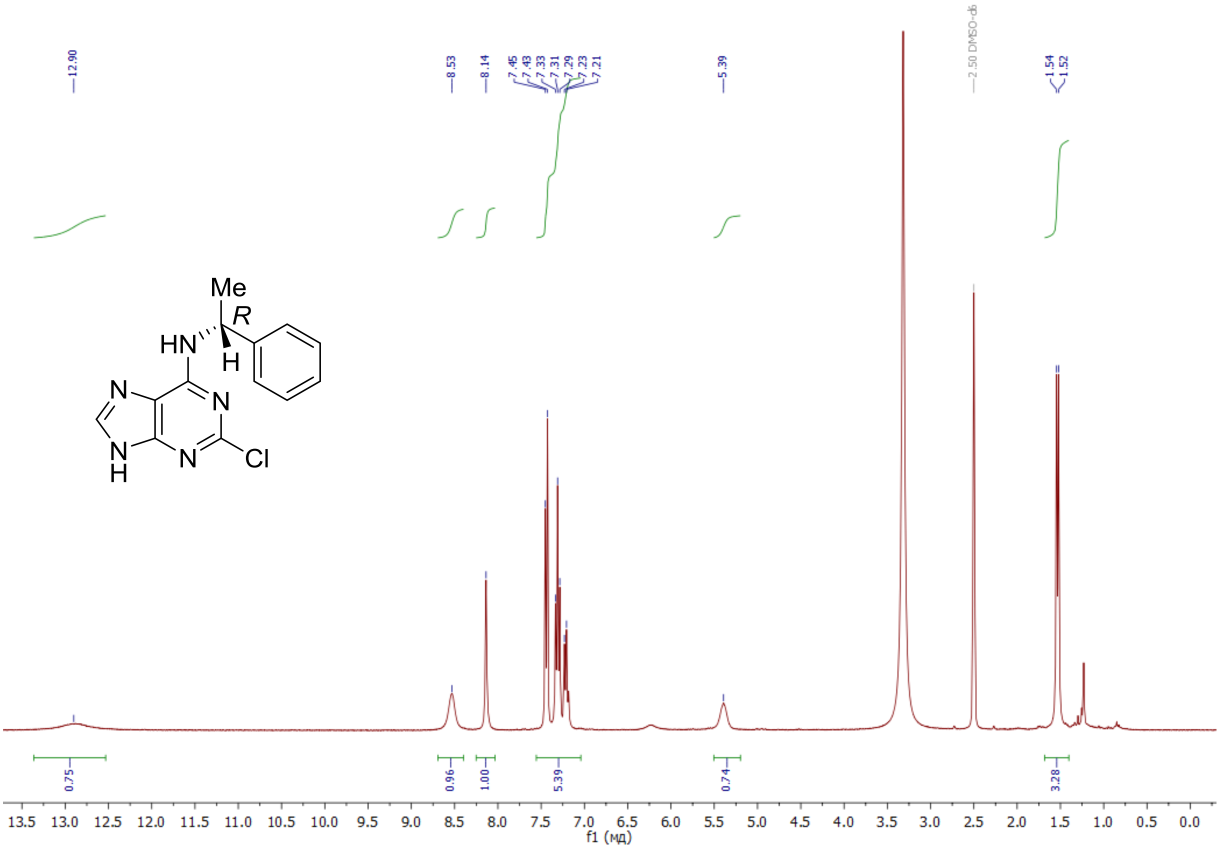

1H-NMR-spectrum (300.1 MHz) of 2-chloro, *N*^{6}-((R)-α-methylbenzyl)adenine (**7a**) in DMSO-d^6 at 303K

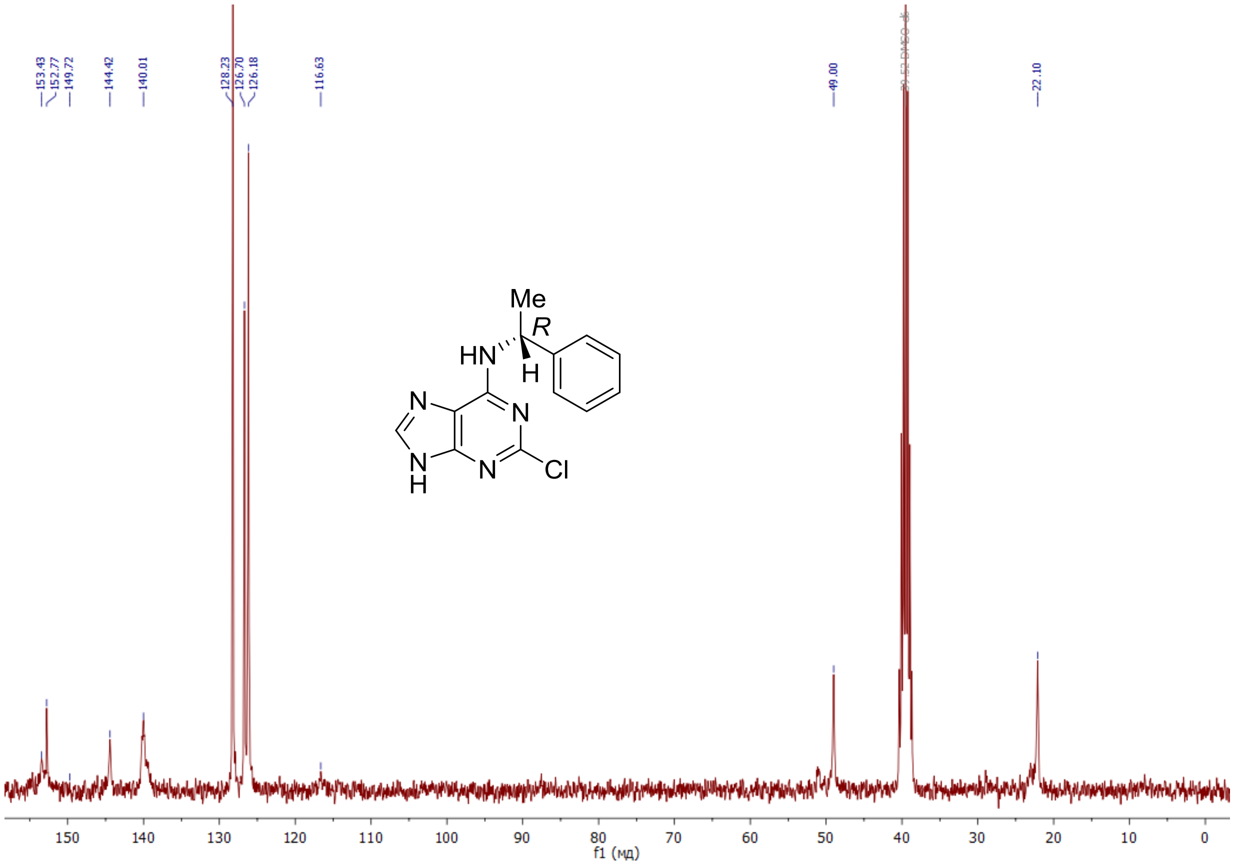

^{13}C-NMR-spectrum (75.5MHz) of 2-chloro, *N*^{6}-((R)-α-methylbenzyl)adenine (**7a)** in DMSO-d^6 at 303K

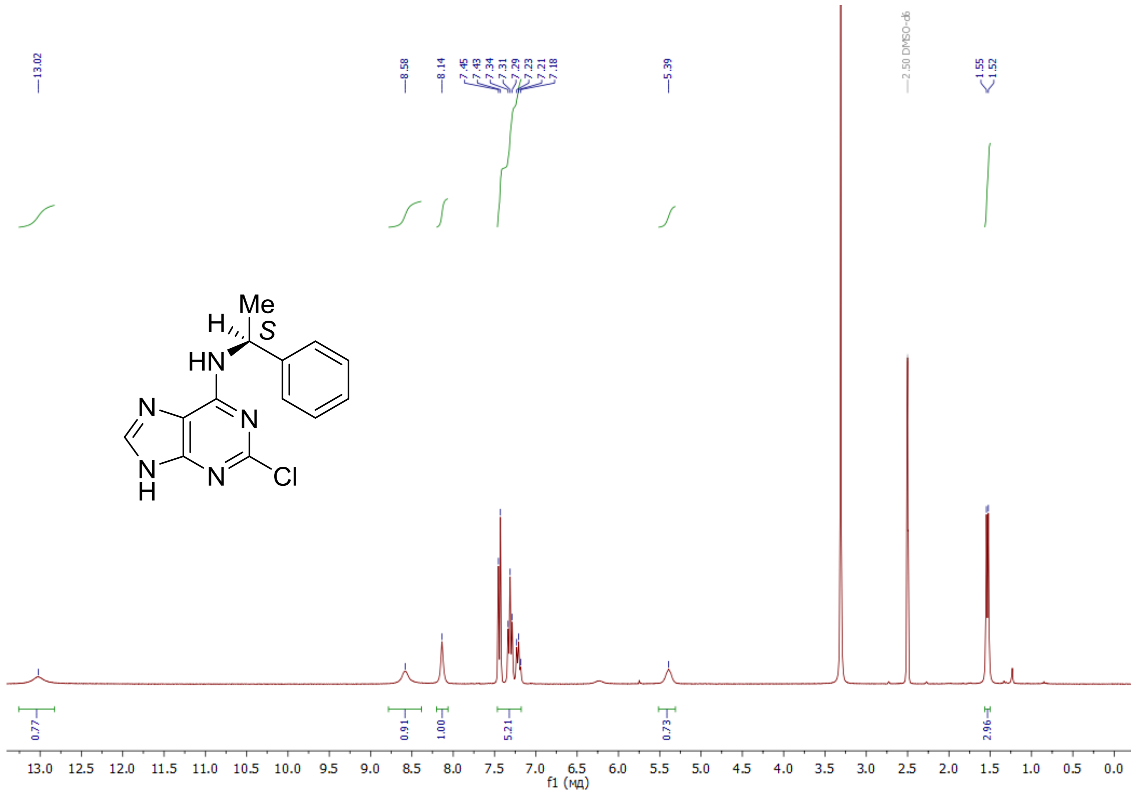

1H-NMR-spectrum (300.1 MHz) of 2-chloro, N^6-((S)-α-methylbenzyl)adenine (**7b**) in DMSO-d^6 at 303K

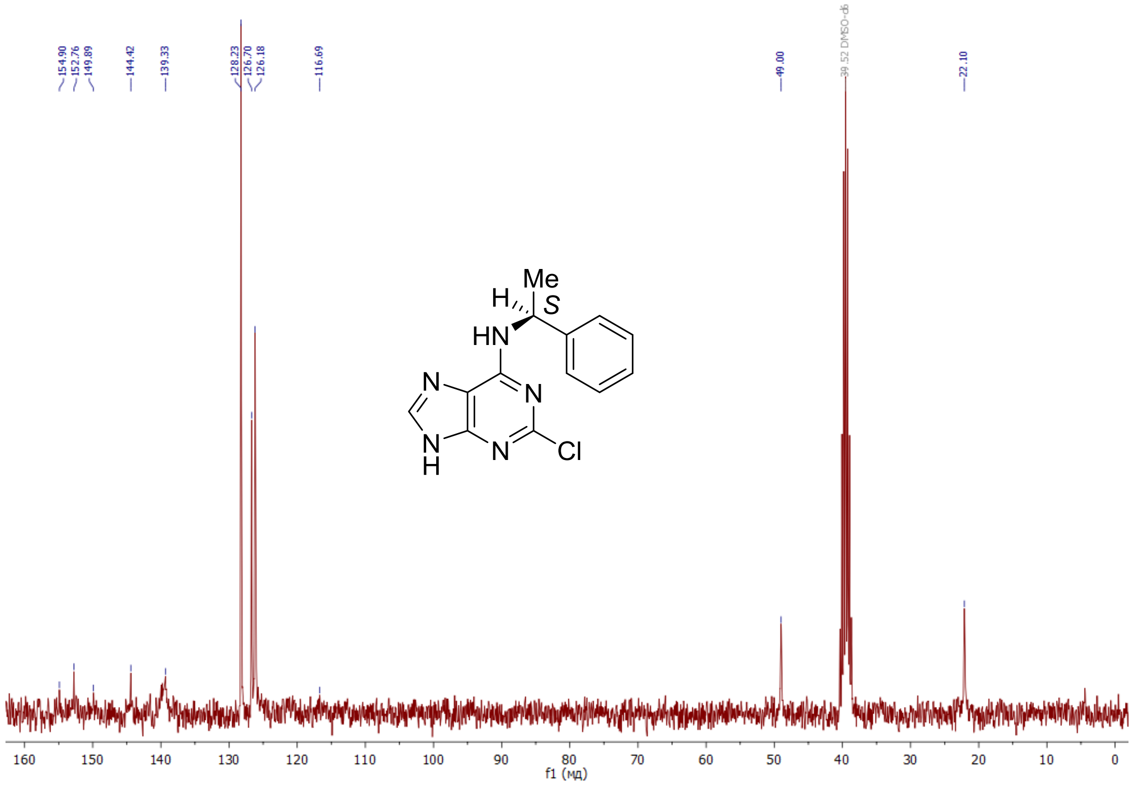

^{13}C-NMR-spectrum (75.5 MHz) of 2-chloro, N^6-((S)-α-methylbenzyl)adenine (**7b**) in DMSO-d^6 at 303K

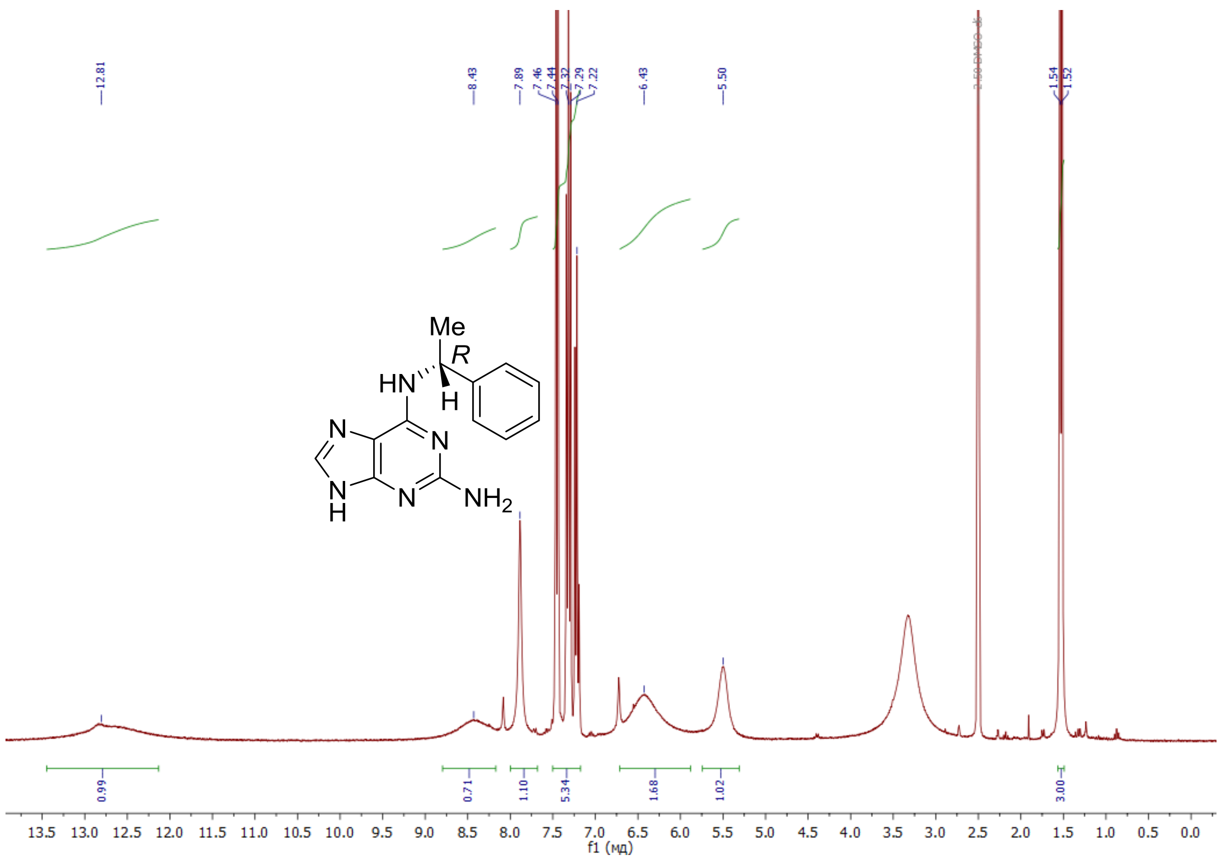

^{1}H-NMR-spectrum (300.1 MHz) of 2-amino, N^6-((R)-α-methylbenzyl)adenine (**8a**) in DMSO-d[6] at 303K

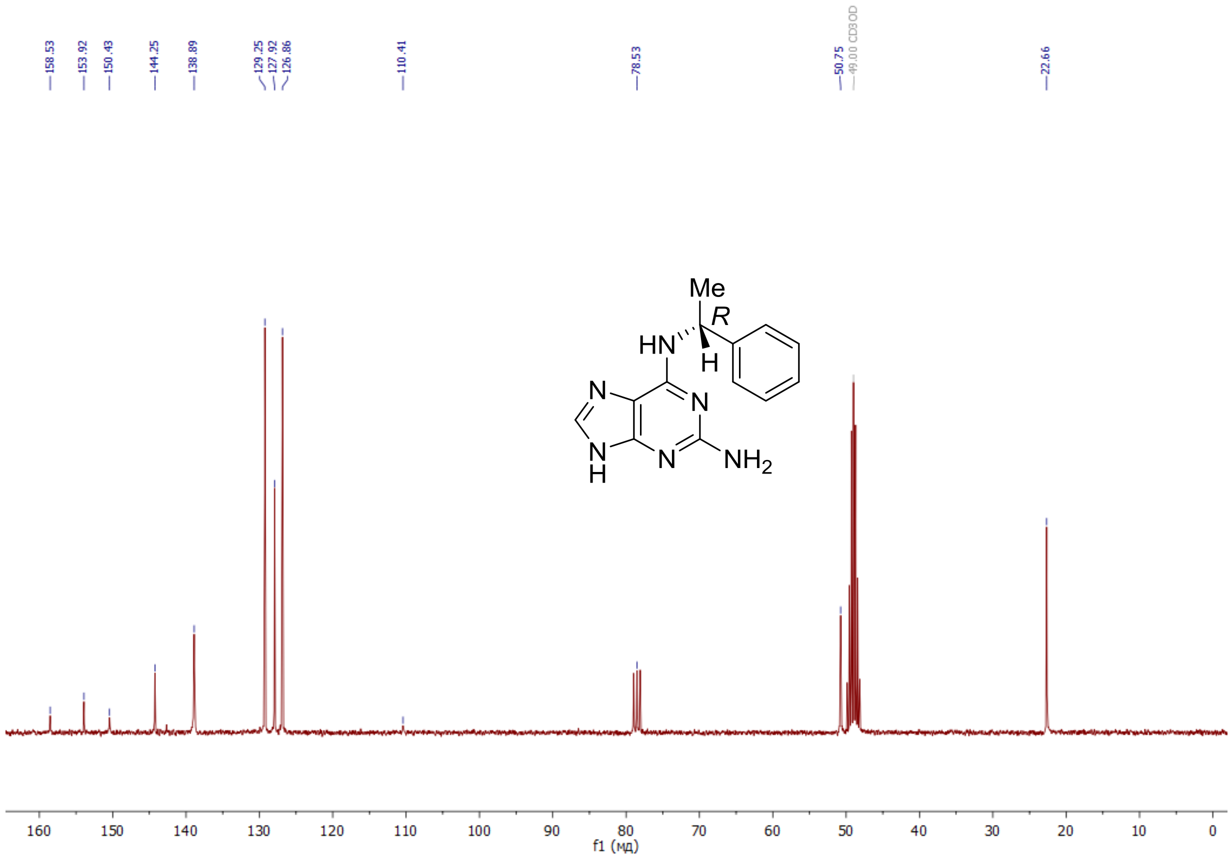

^{13}C-NMR-spectrum (75.5 MHz) of 2-amino, N^6-((R)-α-methylbenzyl)adenine (**8a**) in $CD_3OD/CDCl_3$ at 303K

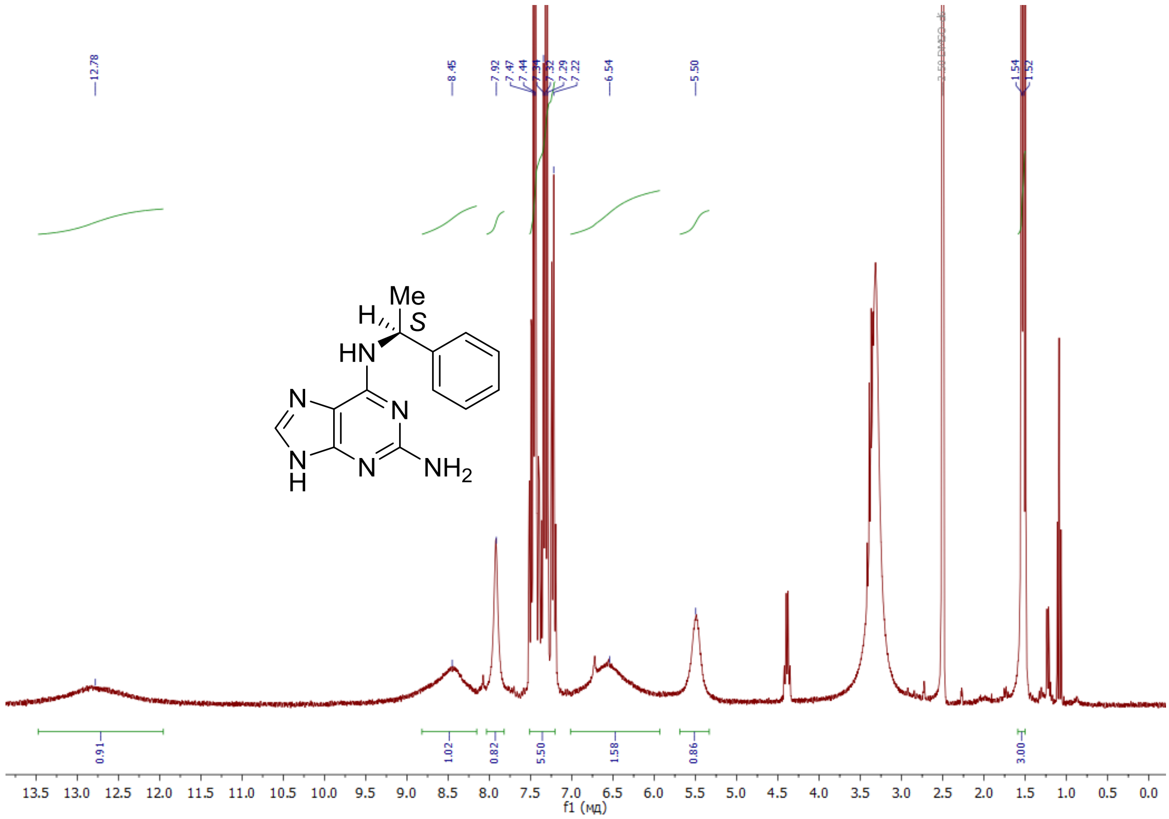

^{1}H-NMR-spectrum (300.1 MHz) of 2-amino, *N*^{6}-((S)-α-methylbenzyl)adenine (**8b**) in DMSO-d^{6} at 303K

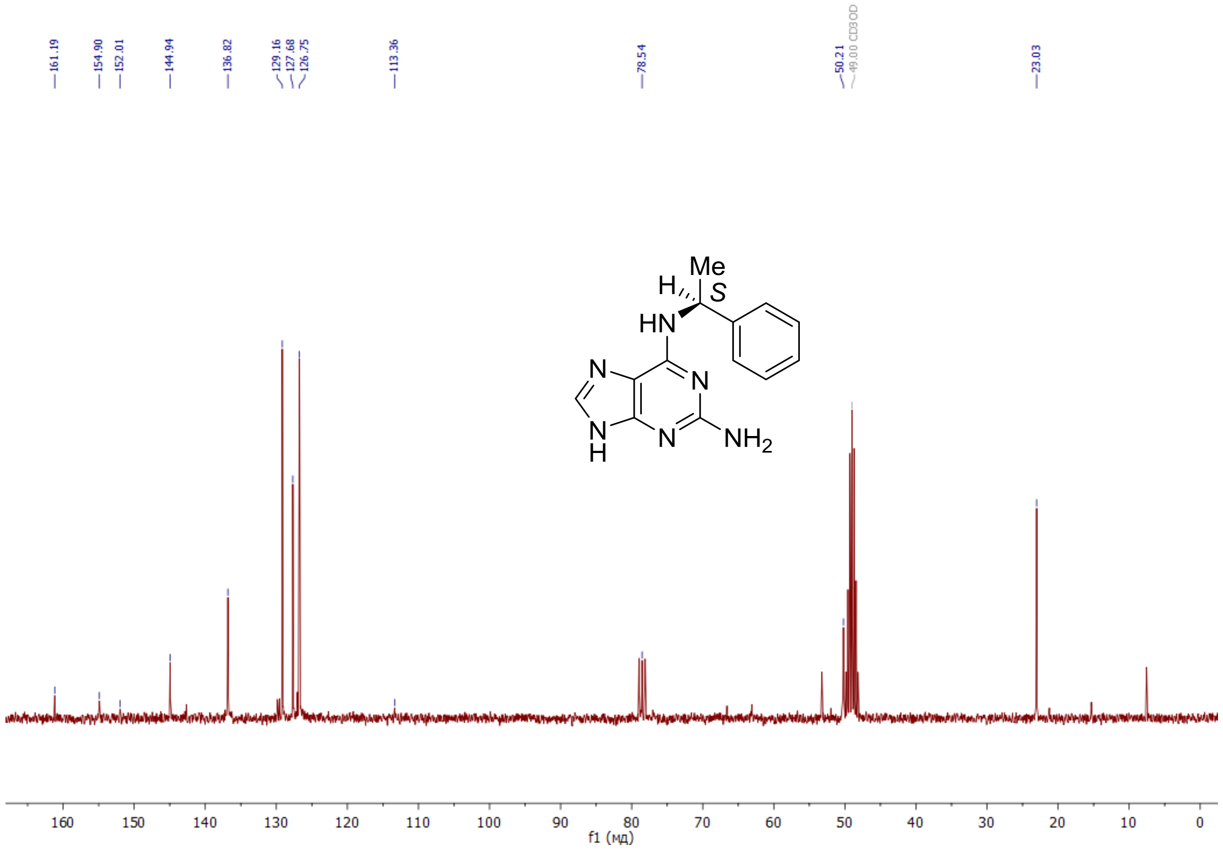

^{13}C-NMR-spectrum (75.5 MHz) of 2-amino, *N*^{6}-((S)-(−)-1-α-methylbenzyl)adenine (**8b**) in $CD_3OD/CDCl_3$ at 303K

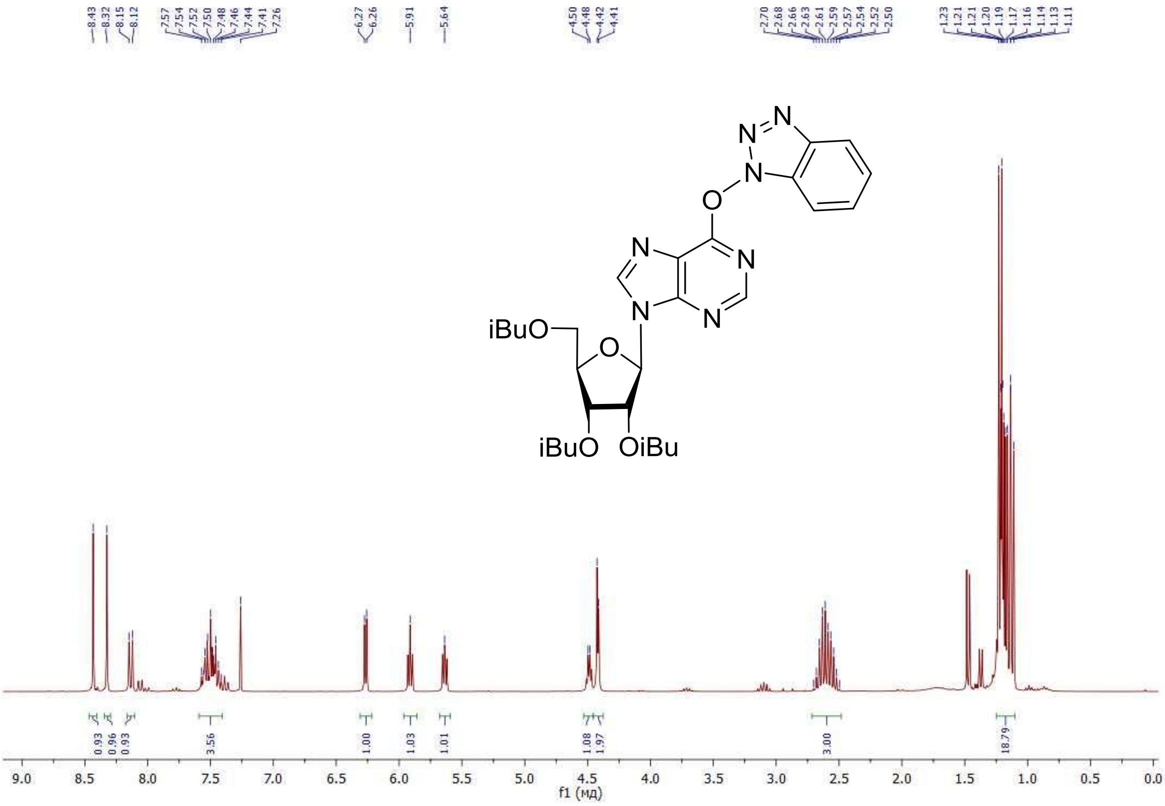

^{1}H-NMR-spectrum (300.1 MHz) of O^6-(benzotriazol-1-yl)-2′,3′,5′-tri-*O*-isobutyroyl inosine (10) in $CDCl_3$ at 303K.

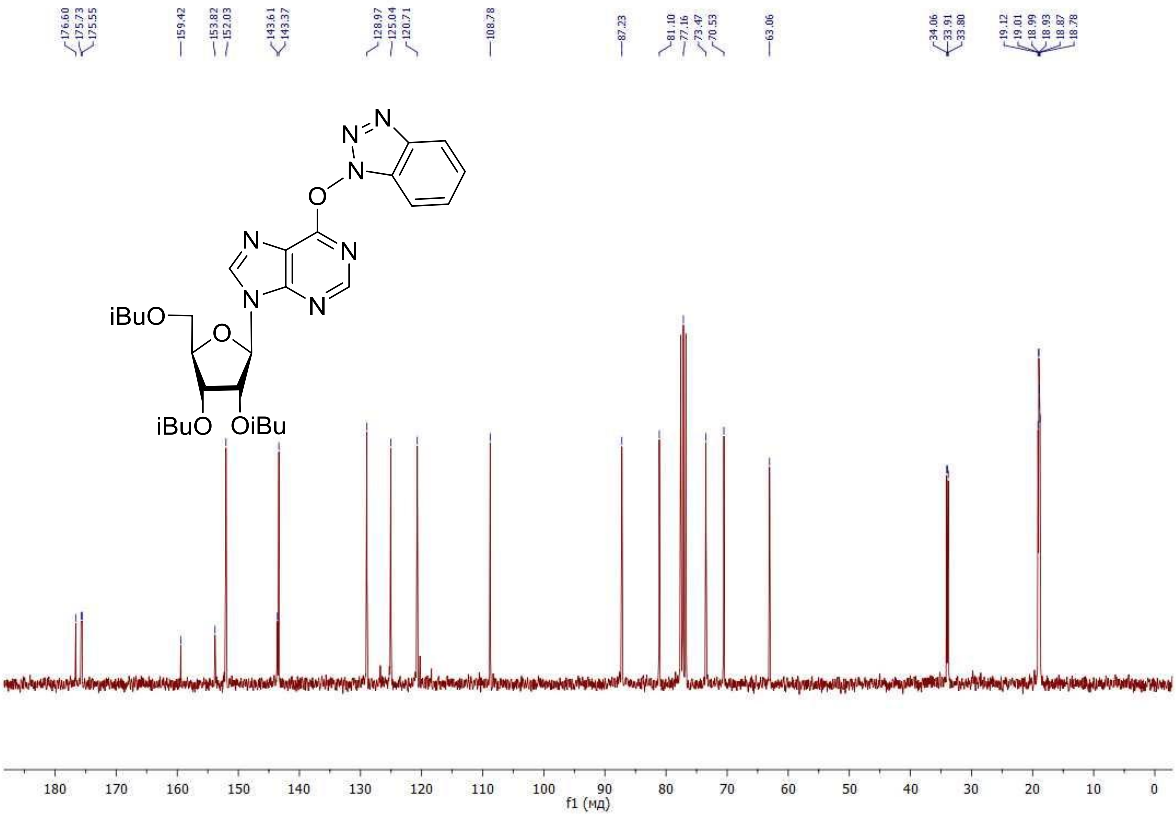

^{13}C-NMR-spectrum (75.5 MHz) of O^6-(benzotriazol-1-yl)-2′,3′,5′-tri-*O*-isobutyroyl inosine (10) in $CDCl_3$ at 303K.

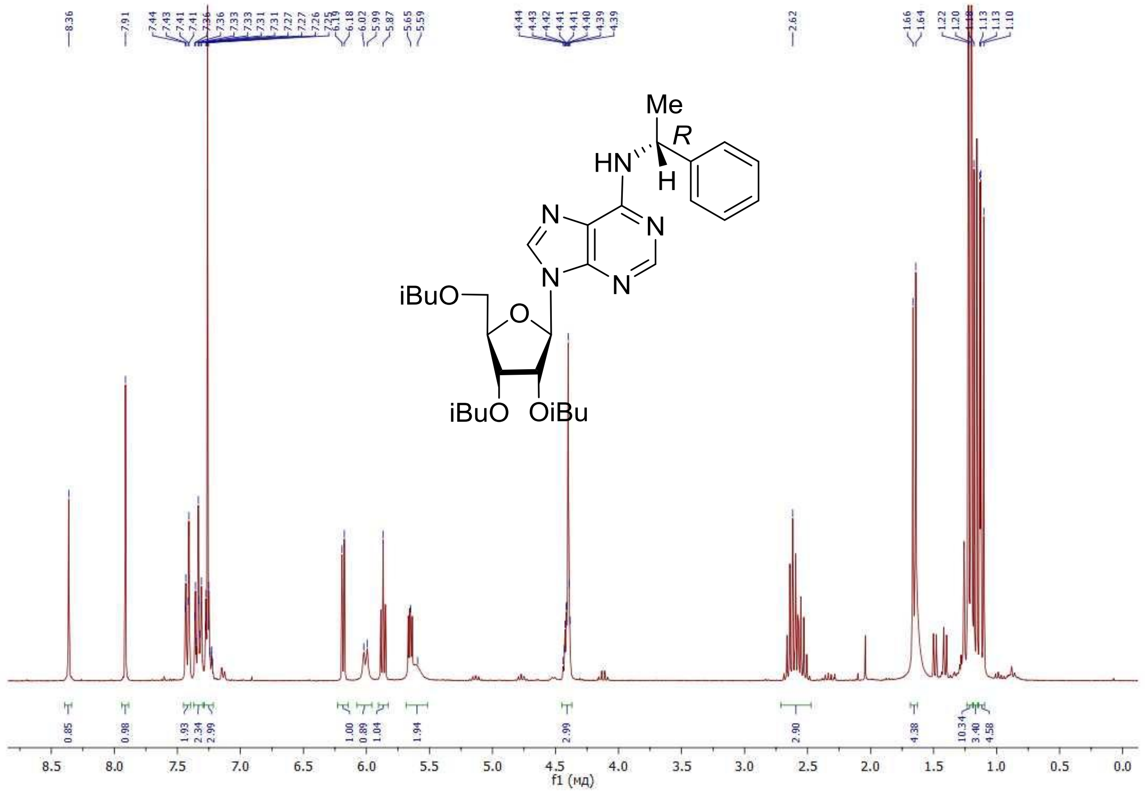

^{1}H-NMR-spectrum (300.1 MHz) of N^6-((R)-α-methylbenzyl)-2′,3′,5′-tri-*O*-isobutyroyl adenosine (**11a**) in $CDCl_3$ at 303K.

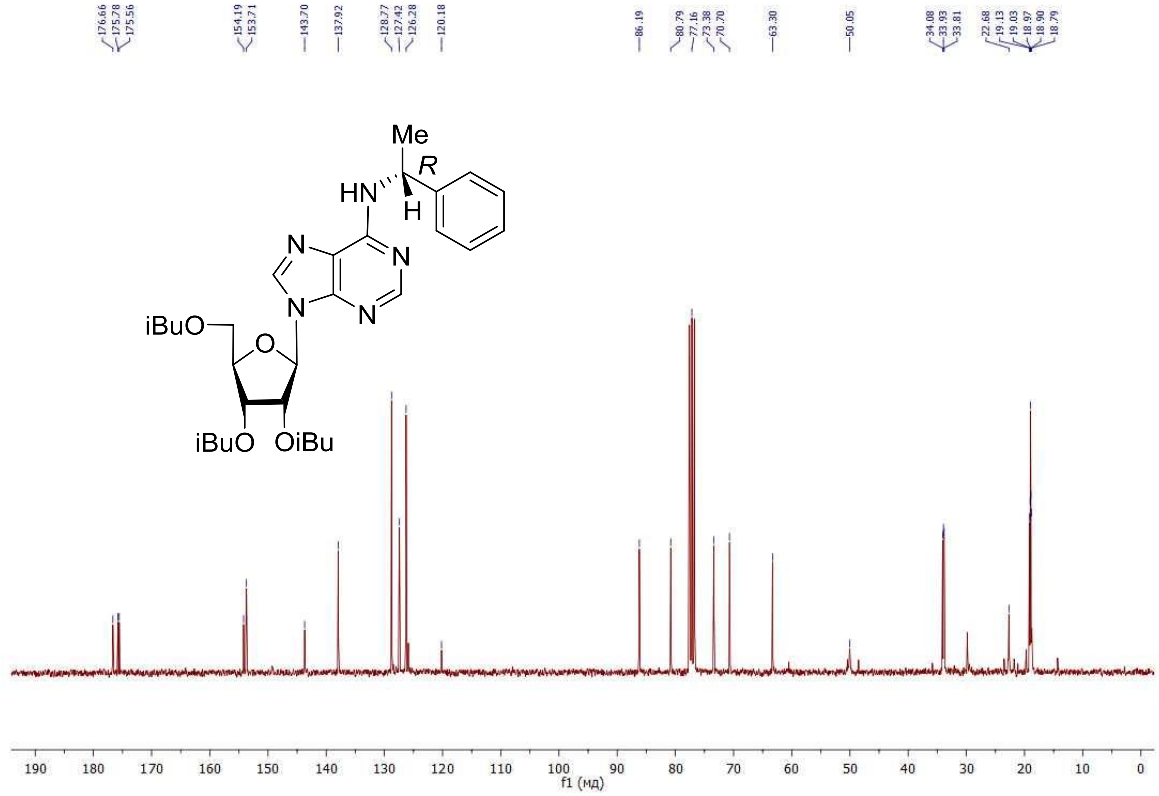

^{13}C-NMR-spectrum (75.5 MHz) of N^6-((R)-α-methylbenzyl)- 2′,3′,5′-tri-*O*-isobutyroyl adenosine (**11a**) in $CDCl_3$ at 303K.

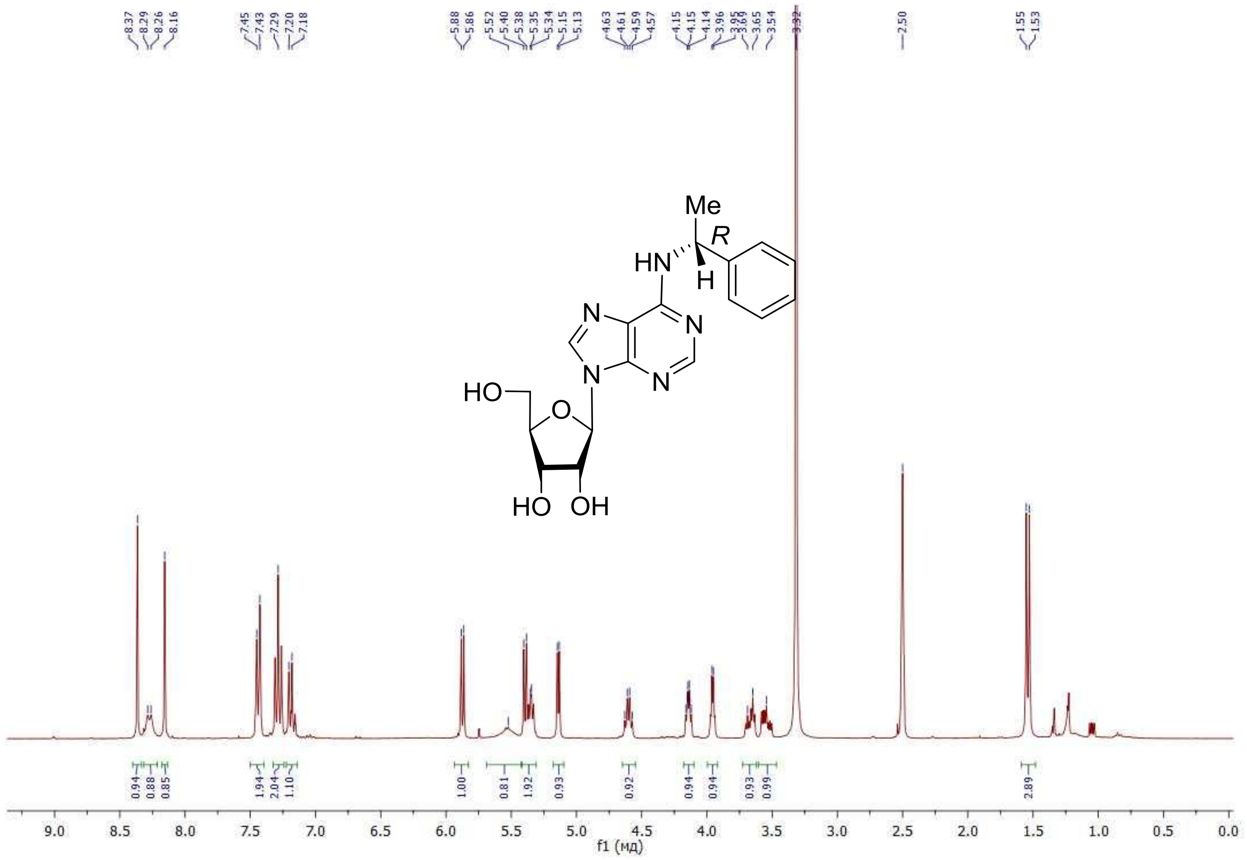

^{1}H-NMR-spectrum (300.1 MHz) of N^6-((R)-α-methylbenzyl)adenosine (**12a**) in DMSO-d_6 at 303K.

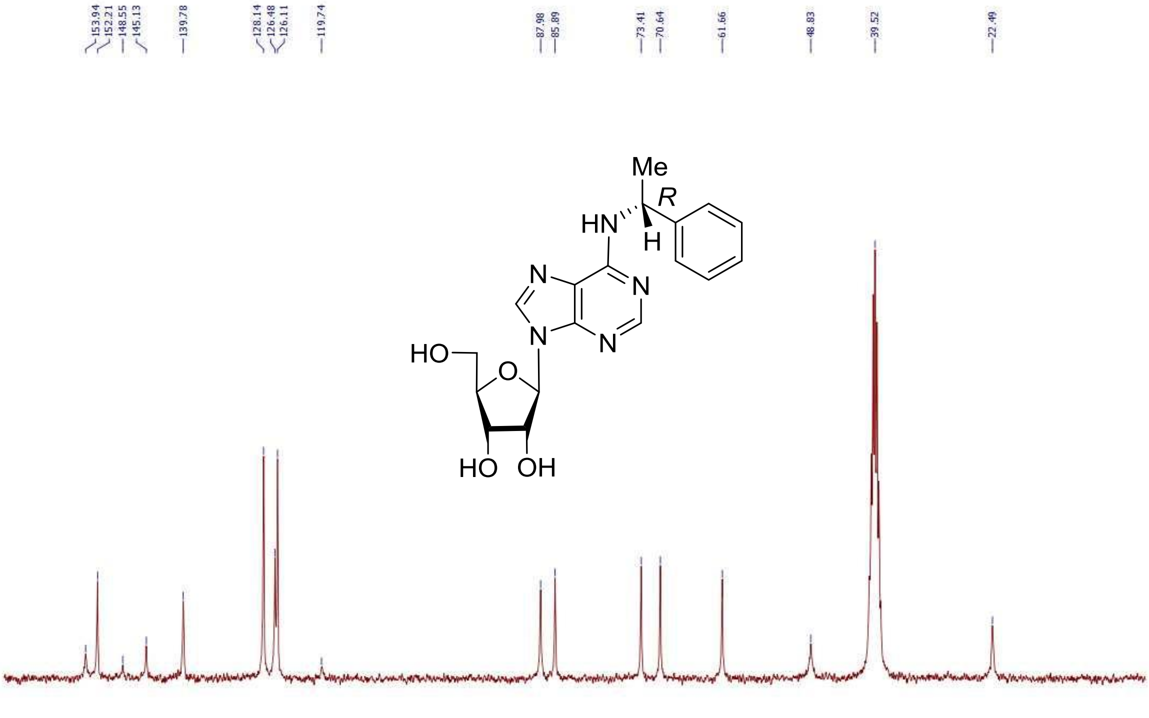

^{13}C-NMR-spectrum (75.5 MHz) of N^6-((R)-α-methylbenzyl)adenosine (**12a**) inDMSO-d_6 at 303K.

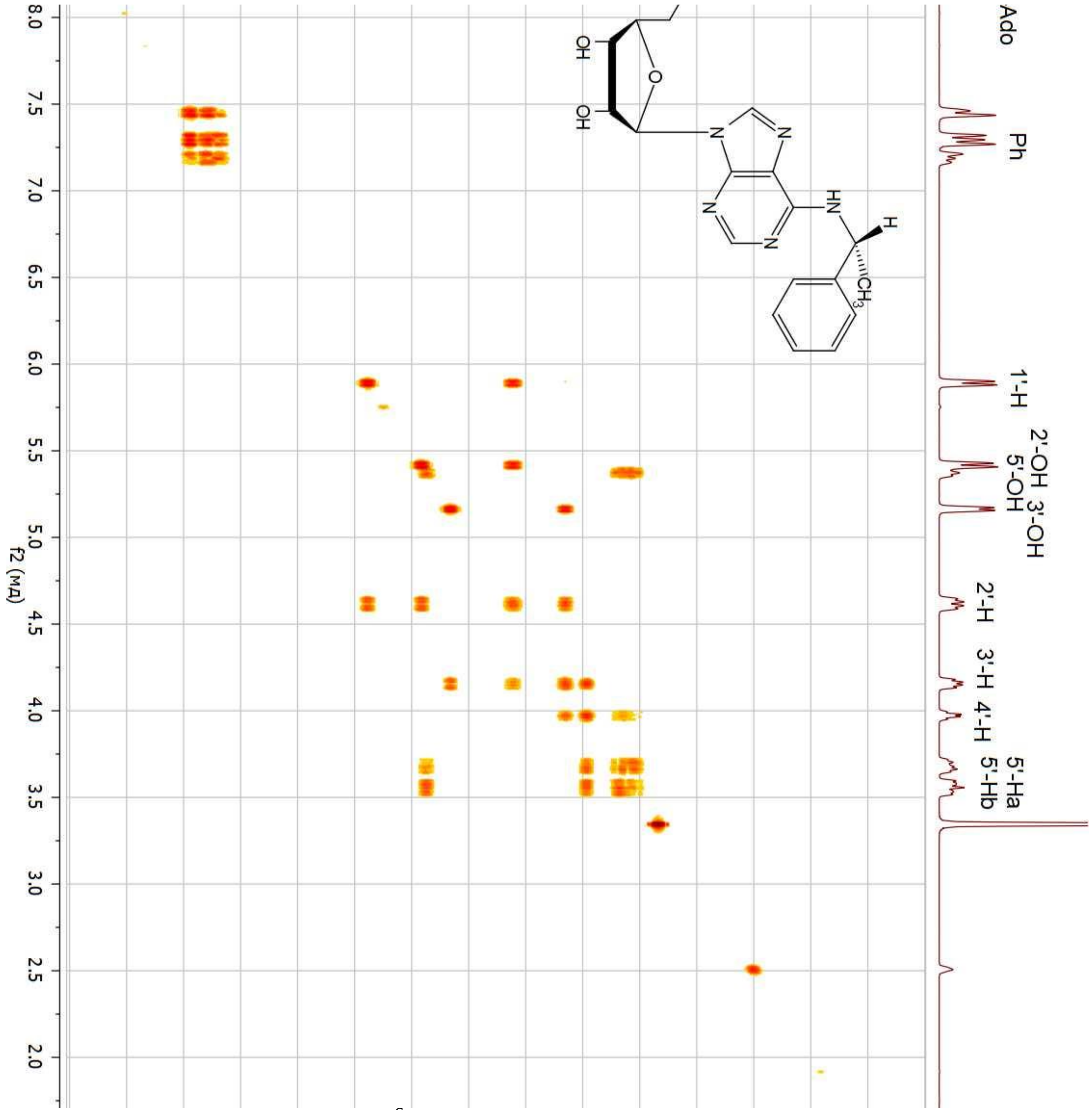

{^{1}H; ^{1}H}-COSY spectrum (300.1 MHz) of N^6-((R)-α-methylbenzyl)adenosine **(12a)** in DMSO-d_6 at 303K.

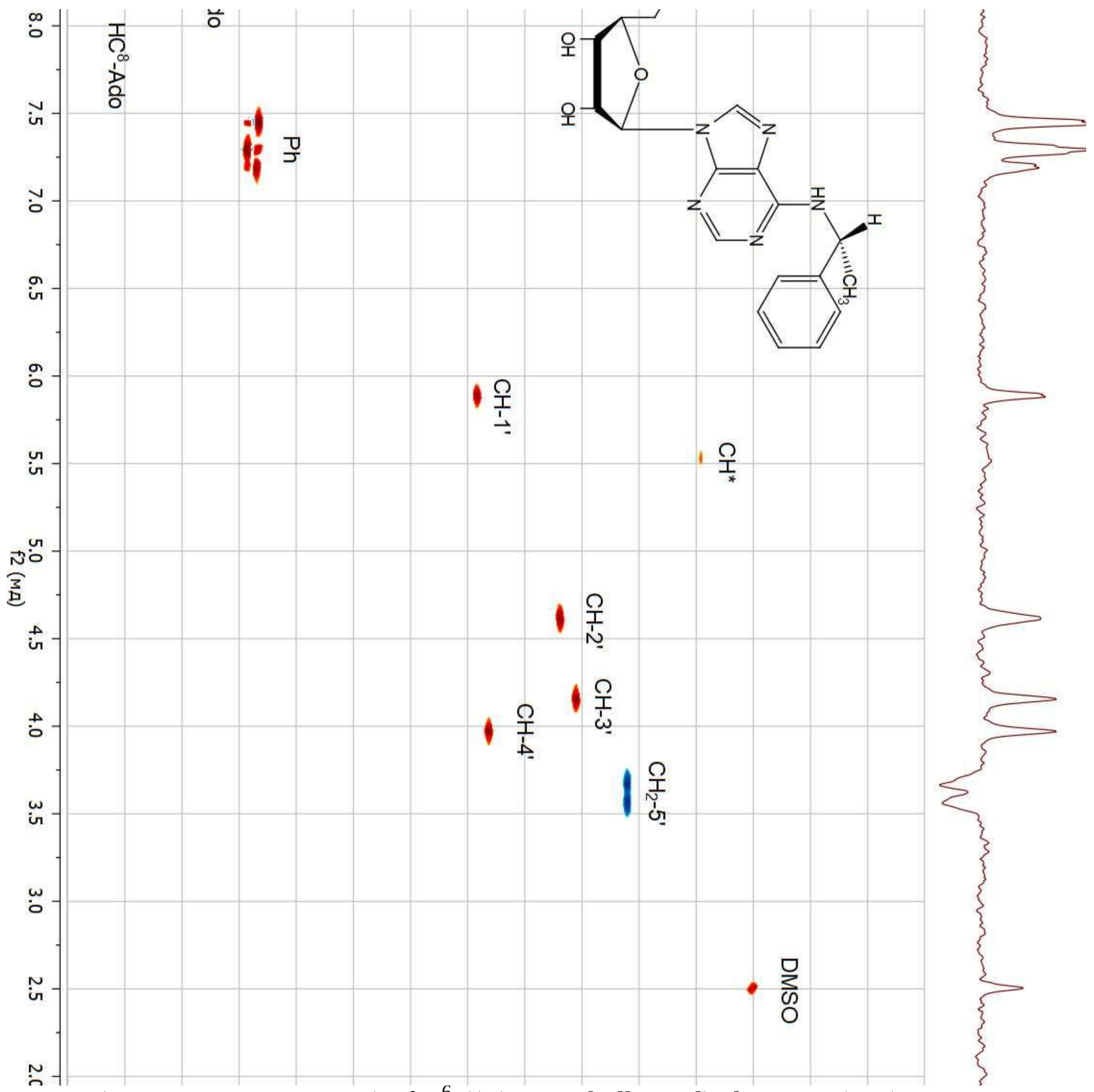

{^{1}H; ^{13}C}-HSQC spectrum (300.1 MHz; 75.5 MHz) of N^6-((R)-α-methylbenzyl)adenosine (**12a**) in DMSO-d_6 at 303K.

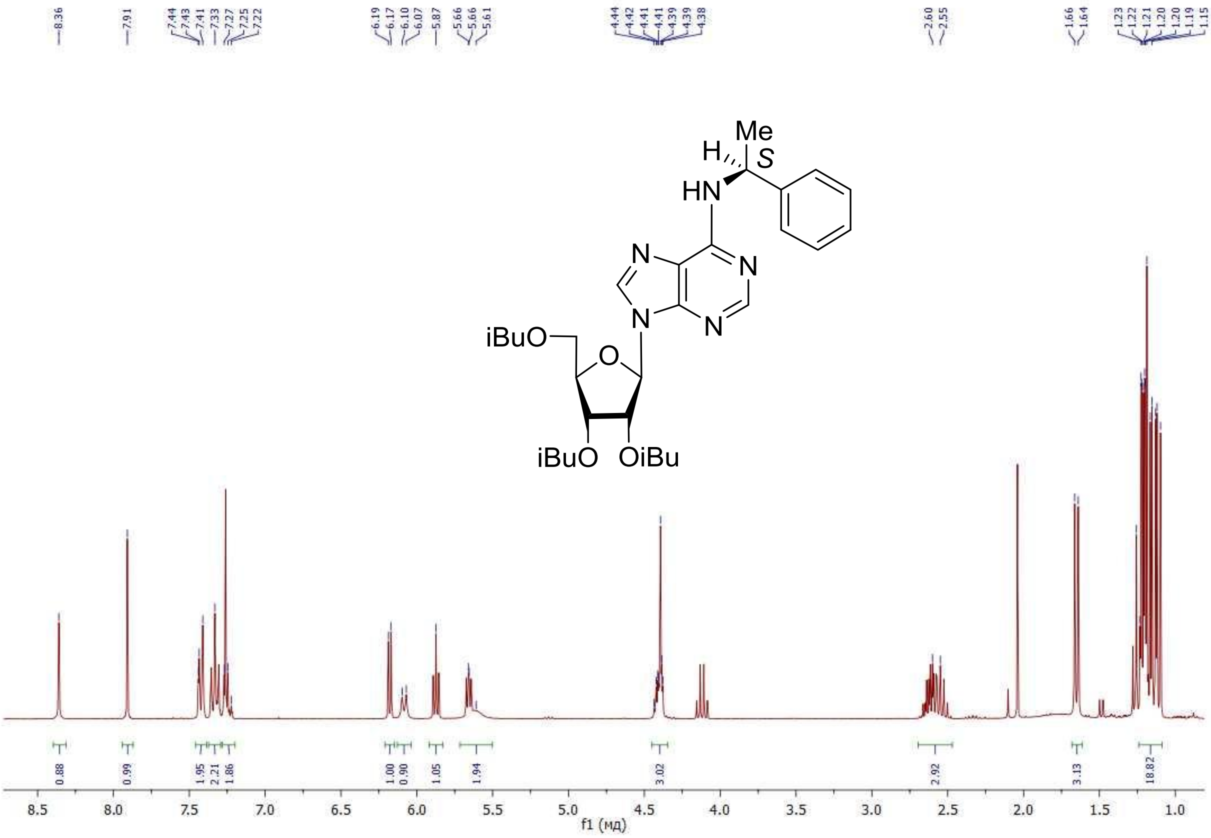

^{1}H-NMR-spectrum (300.1 MHz) of *N*[6]-((S)-α-methylbenzyl)-2′,3′,5′-tri-*O*-isobutyroyl adenosine (**11b**) in $CDCl_3$ at 303K.

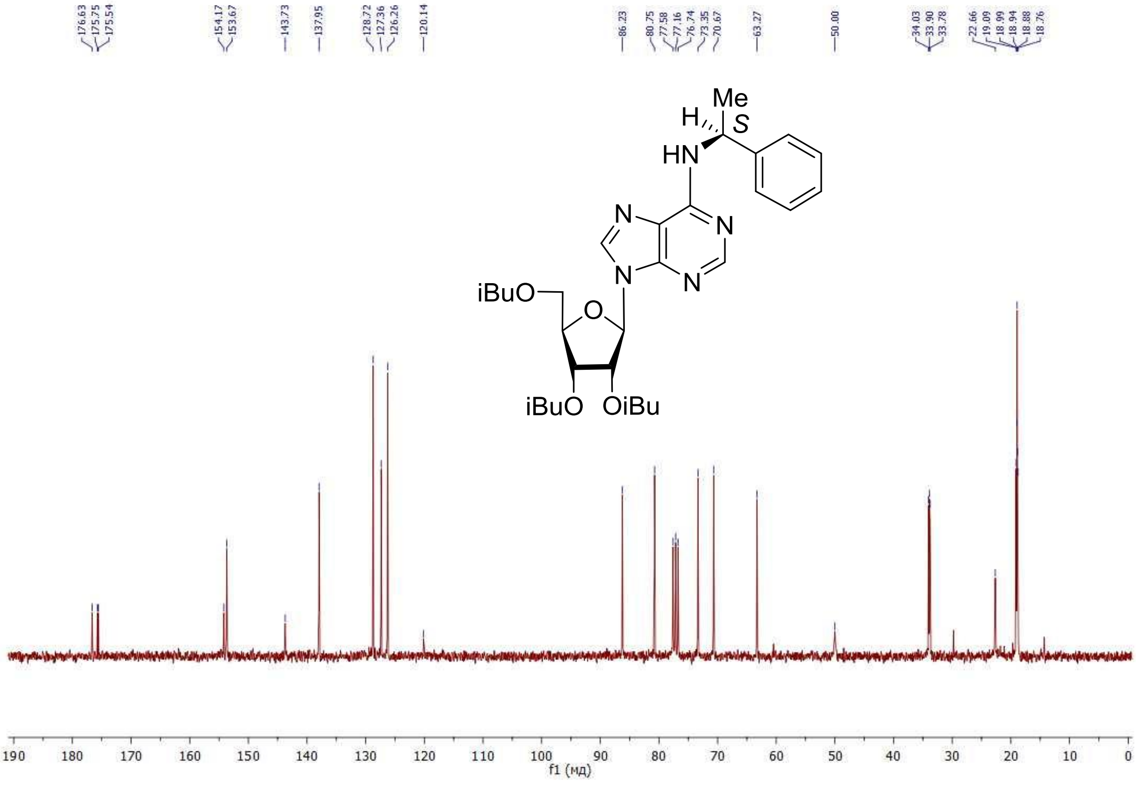

^{13}C-NMR-spectrum (75.5 MHz) of *N*[6]-((S)-α-methylbenzyl)-2′,3′,5′-tri-*O*-isobutyroyl adenosine (11b) in $CDCl_3$ at 303K.

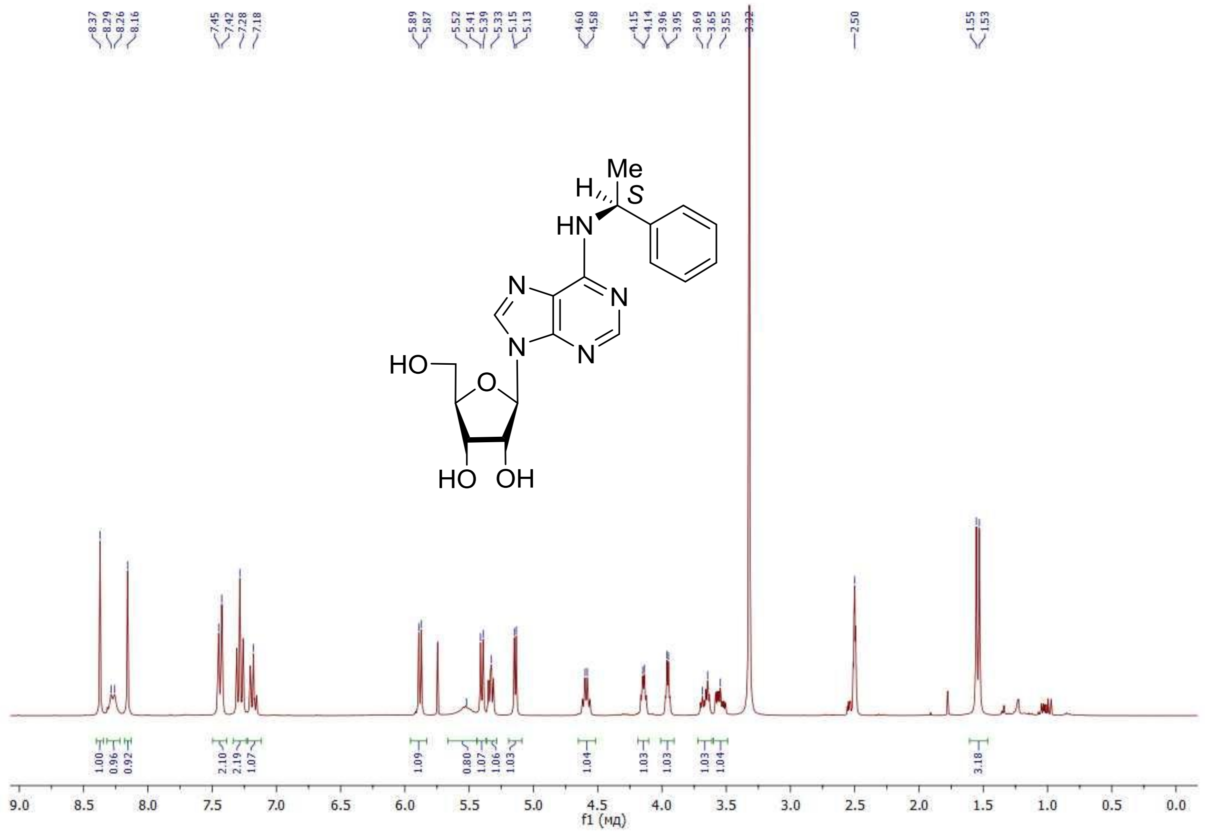

^{1}H-NMR-spectrum (300.1 MHz) of N^6-((S)-α-methylbenzyl)adenosine (**12b**) in DMSO-d_6 at 303K

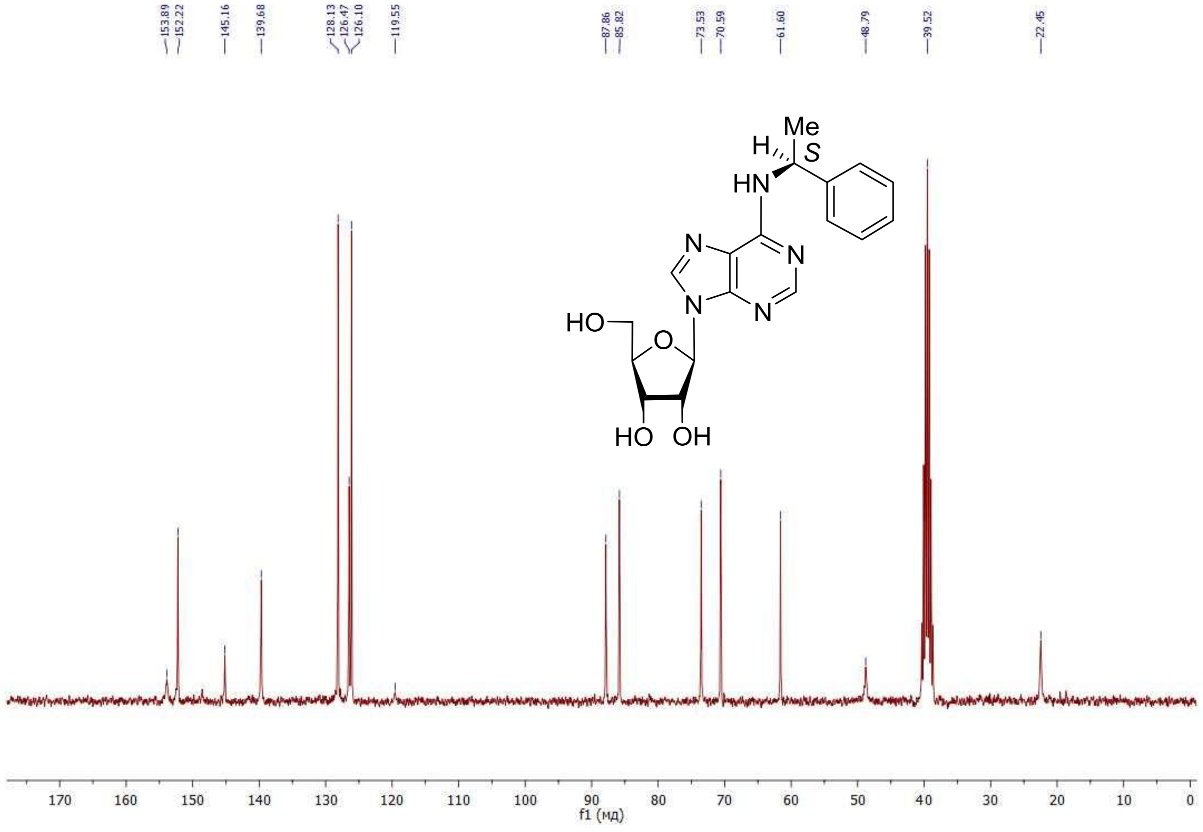

^{13}C-NMR-spectrum (75.5 MHz) of N^6-((S)-α-methylbenzyl)adenosine (**12b**) in DMSO-d_6 at 303K

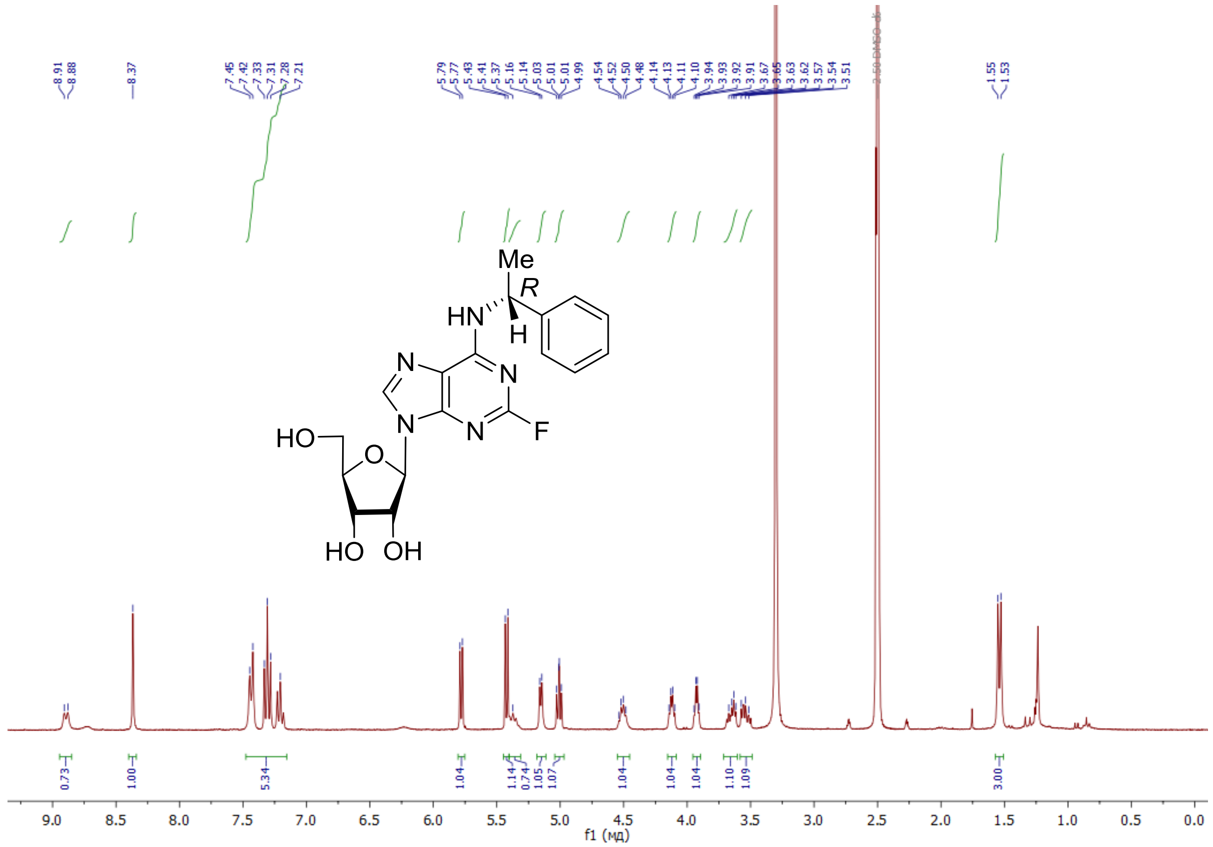

1H-NMR-spectrum (300.1 MHz) of 2-fluoro, N^6-((R)-α-methylbenzyl)adenosine (**13a**) in DMSO-d^6 at 303K

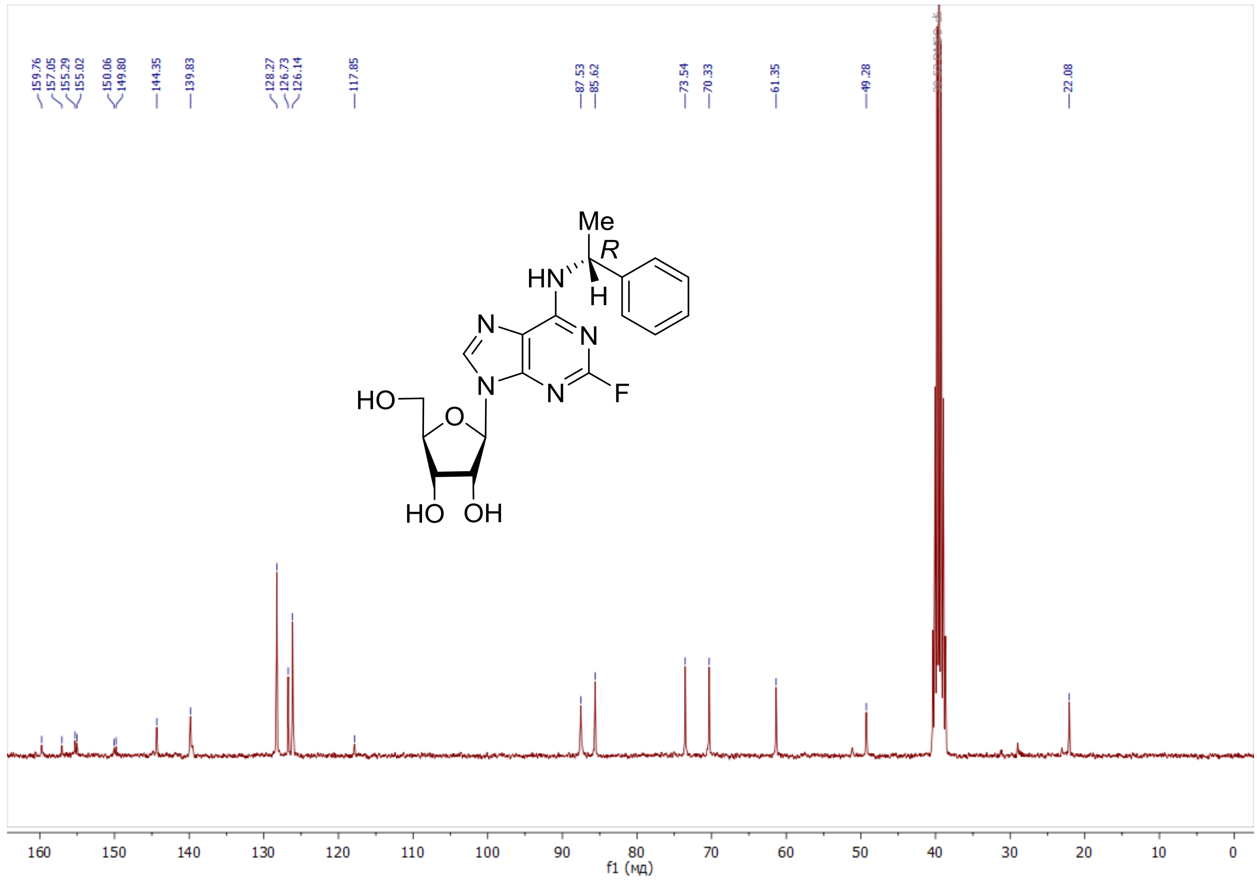

^{13}C-NMR-spectrum (75.5MHz) of 2-fluoro, N^6-((R)-α-methylbenzyl)adenosine (**13a**) in DMSO-d^6 at 303K

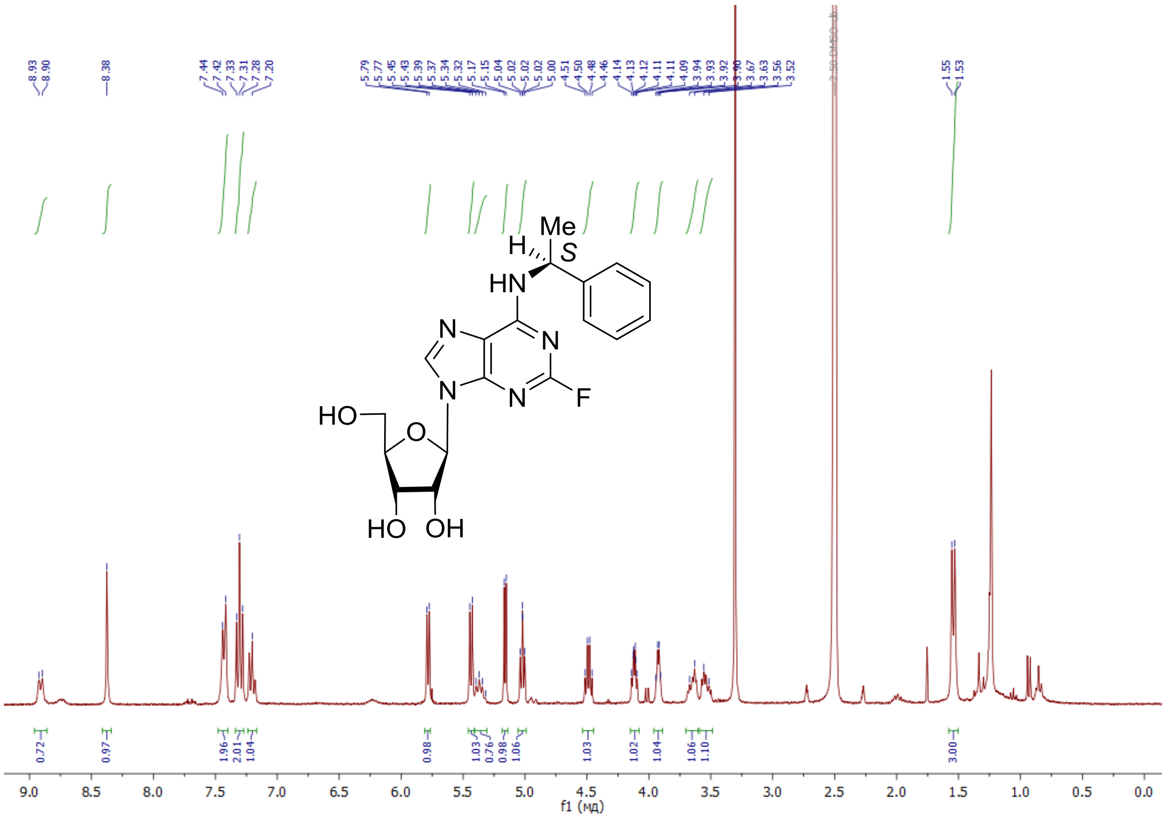

1H-NMR-spectrum (300.1 MHz) of 2-fluoro, *N*^{6}-((S)-α-methylbenzyl)adenosine (**13b**) in DMSO-d^6 at 303K

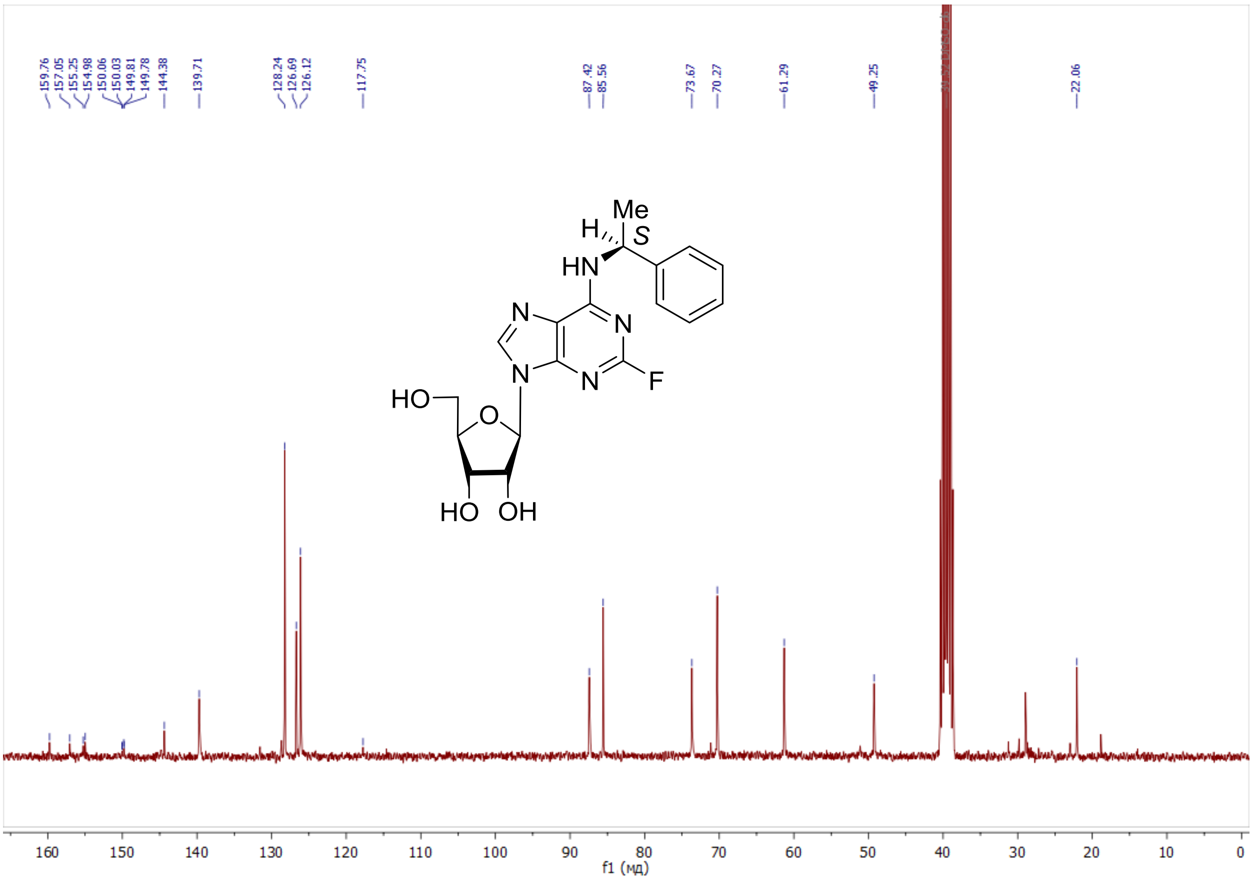

^{13}C-NMR-spectrum (75.5 MHz) of 2-fluoro, *N*^{6}-((S)-α-methylbenzyl)adenosine (**13b**) in DMSO-d^6 at 303K

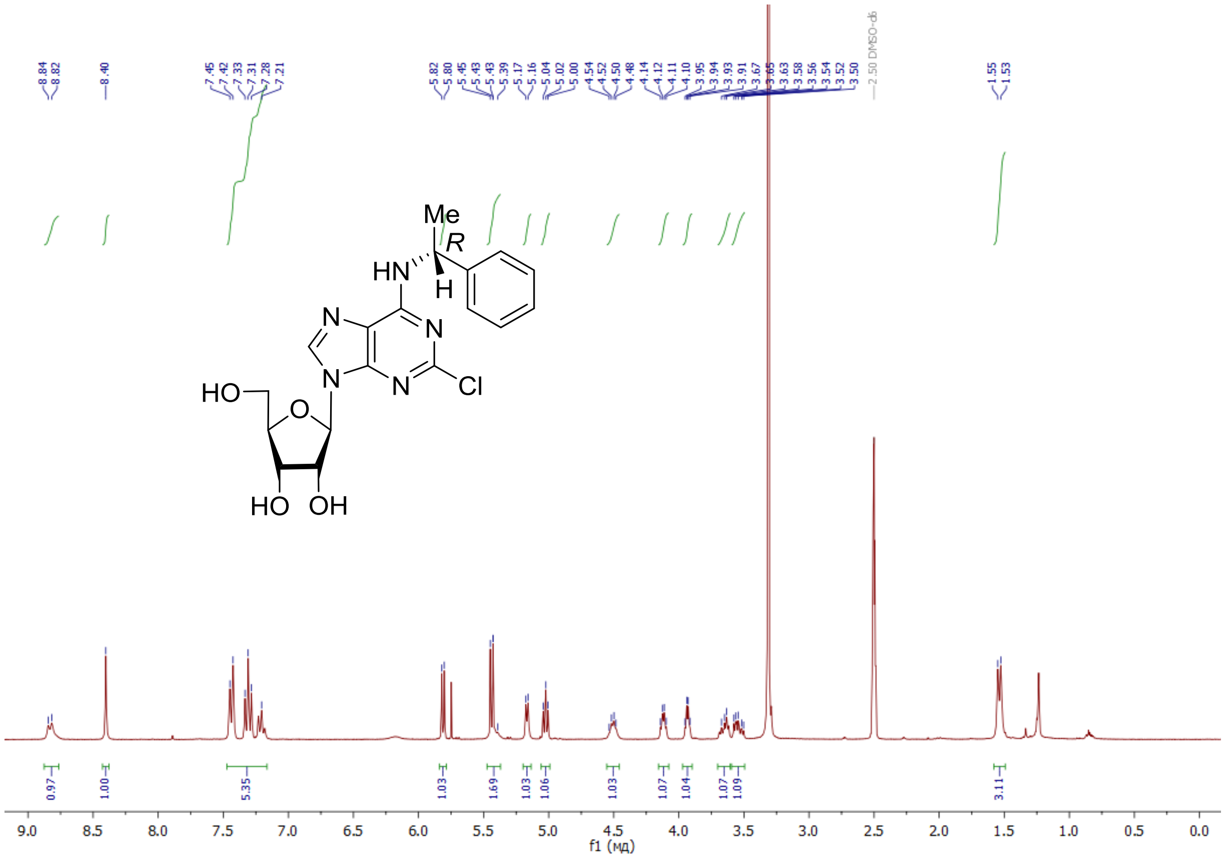

1H-NMR-spectrum (300.1 MHz) of 2-chloro, *N*^{6}-((R)-α-methylbenzyl)adenosine (**14a**) in DMSO-d^{6} at 303K

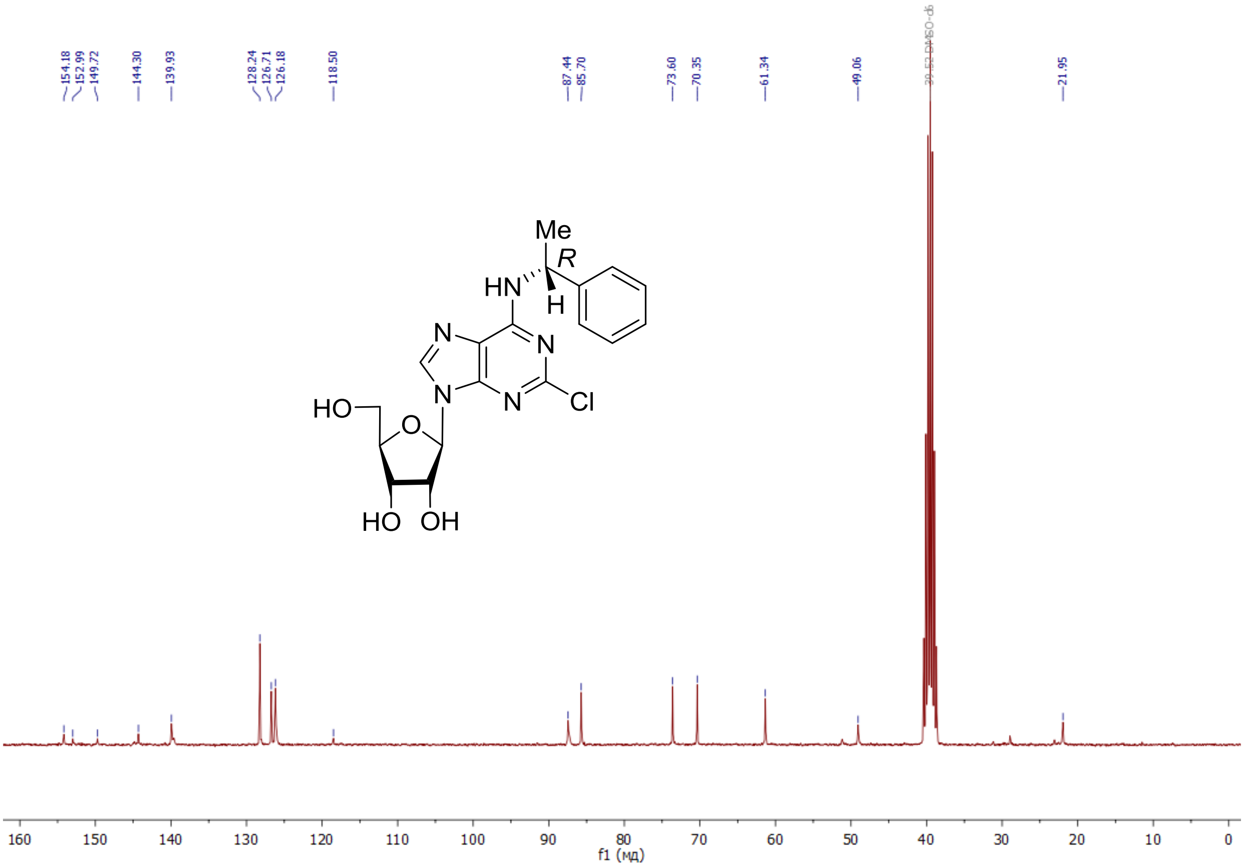

^{13}C-NMR-spectrum (75.5 MHz) of 2-chloro, *N*^{6}-((R)-α-methylbenzyl)adenosine (**14a**) in DMSO-d^{6} at 303K

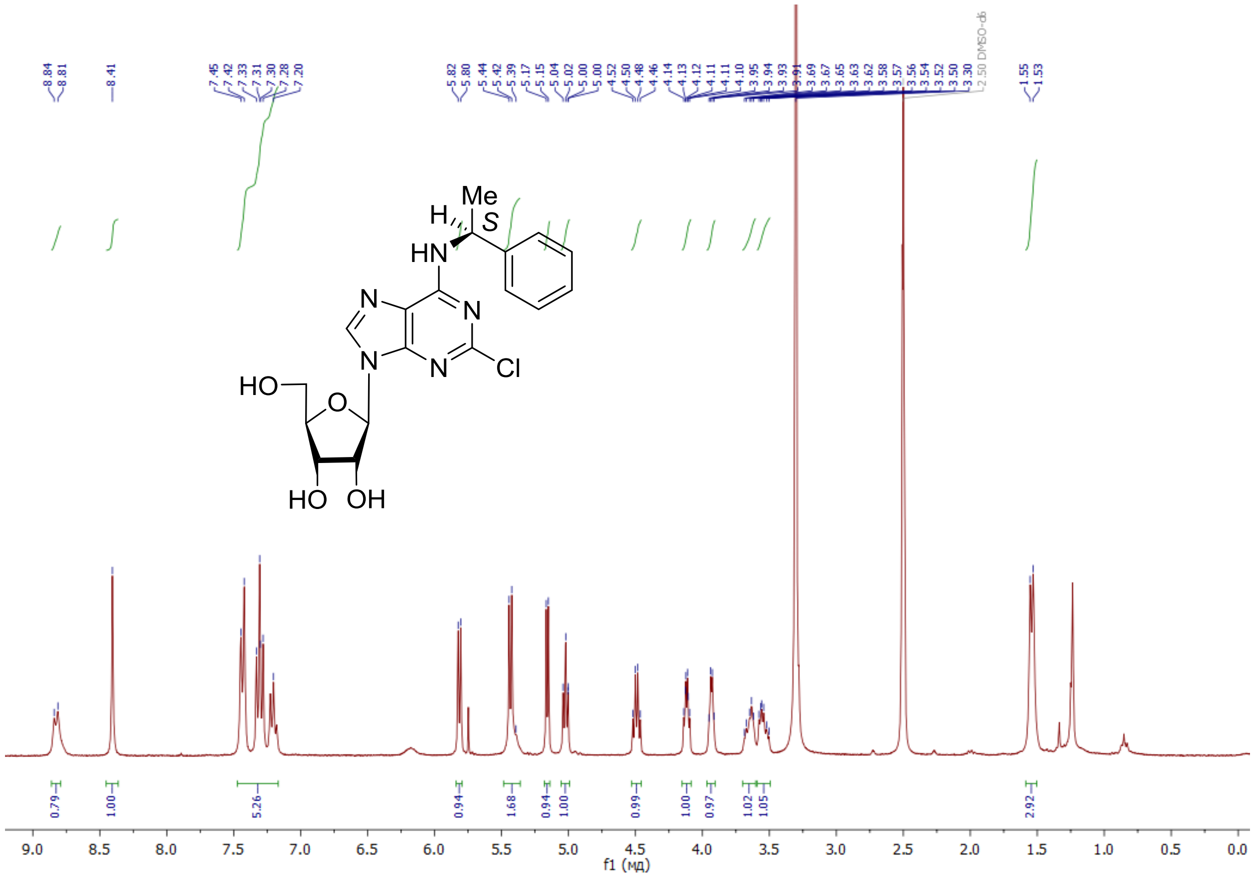

1H-NMR-spectrum (300.1 MHz) of 2-chloro, *N*^{6}-((S)-α-methylbenzyl)adenosine (**14b**) in DMSO-d^{6} at 303K

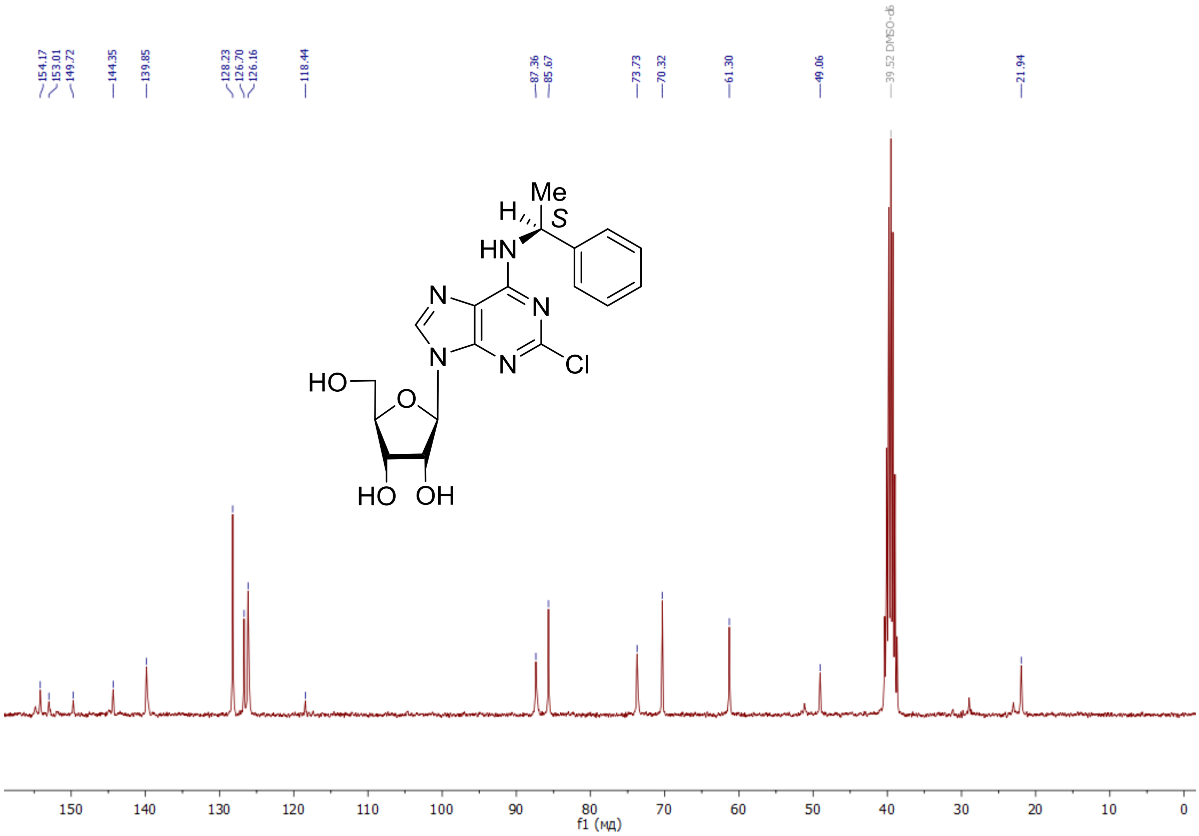

13C-NMR-spectrum (75.4 MHz) of 2-chloro, *N*[6]-((S)-α-methylbenzyl)adenosine (**14b)** in DMSO-d[6] at 303K

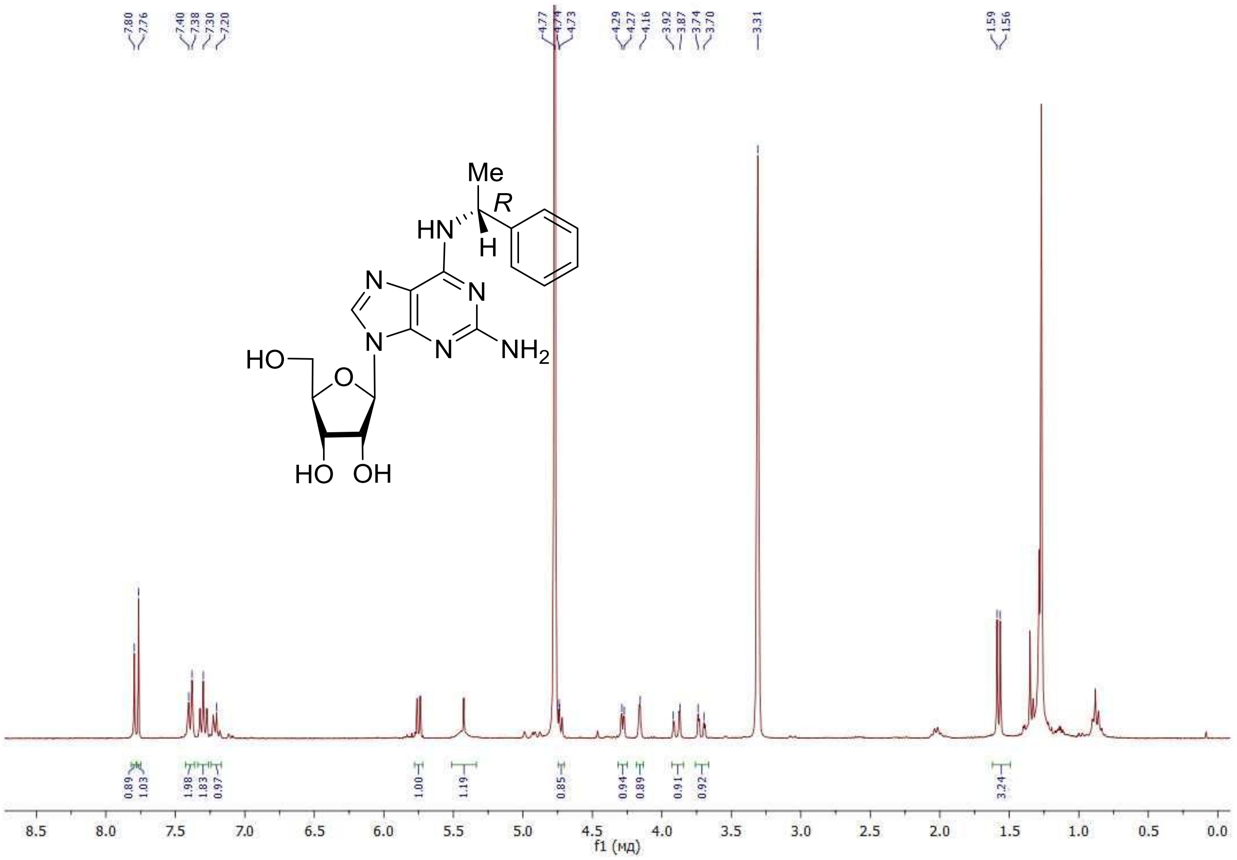

^{1}H-NMR-spectrum (300.1 MHz) of 2-amino, *N*[6]-((R)-α-methylbenzyl)adenosine (**16a**) in CD_3OD-$CDCl_3$– 9:1 (*v*/*v*) at 303K.

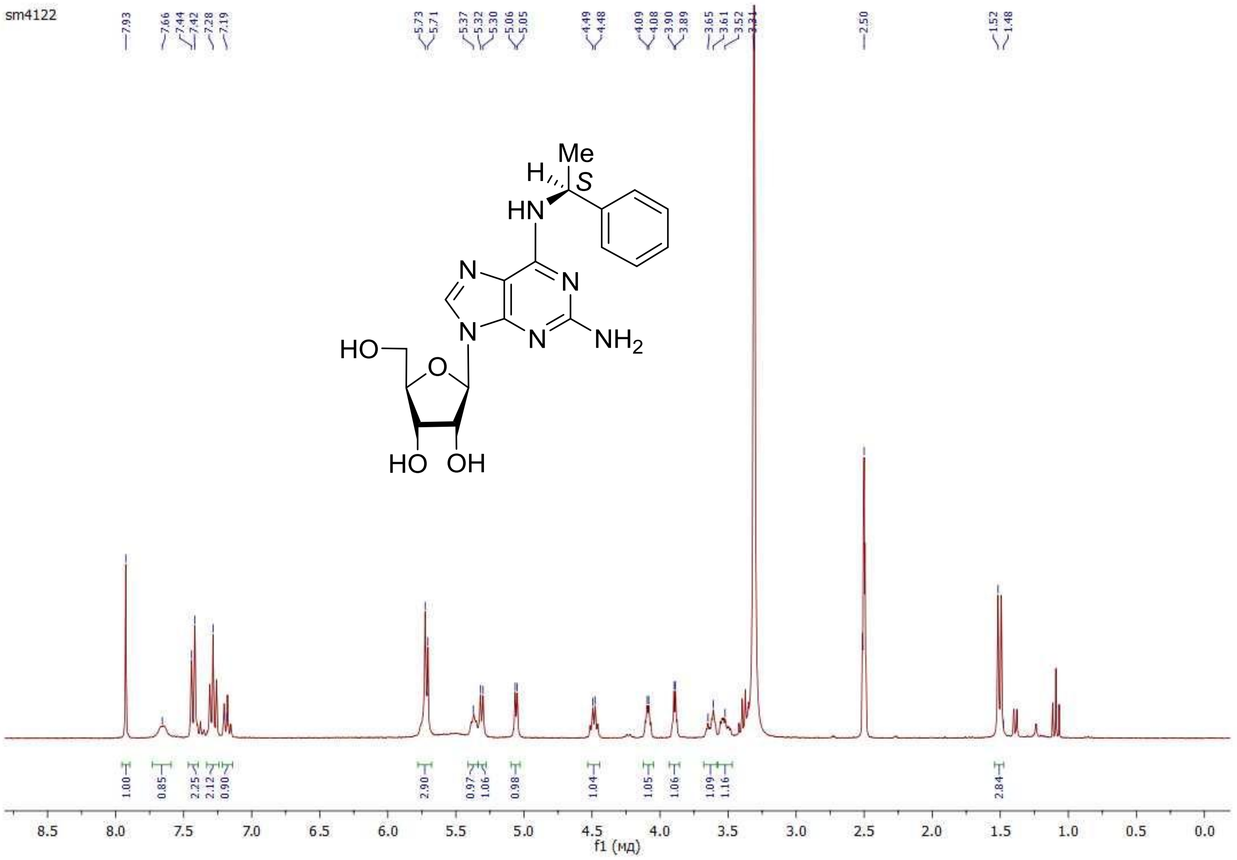

^{1}H-NMR-spectrum (300.1 MHz) of 2-amino, N^6-((S)-α-methylbenzyl)adenosine (**16b**) in DMSO-d_6 at 303K.

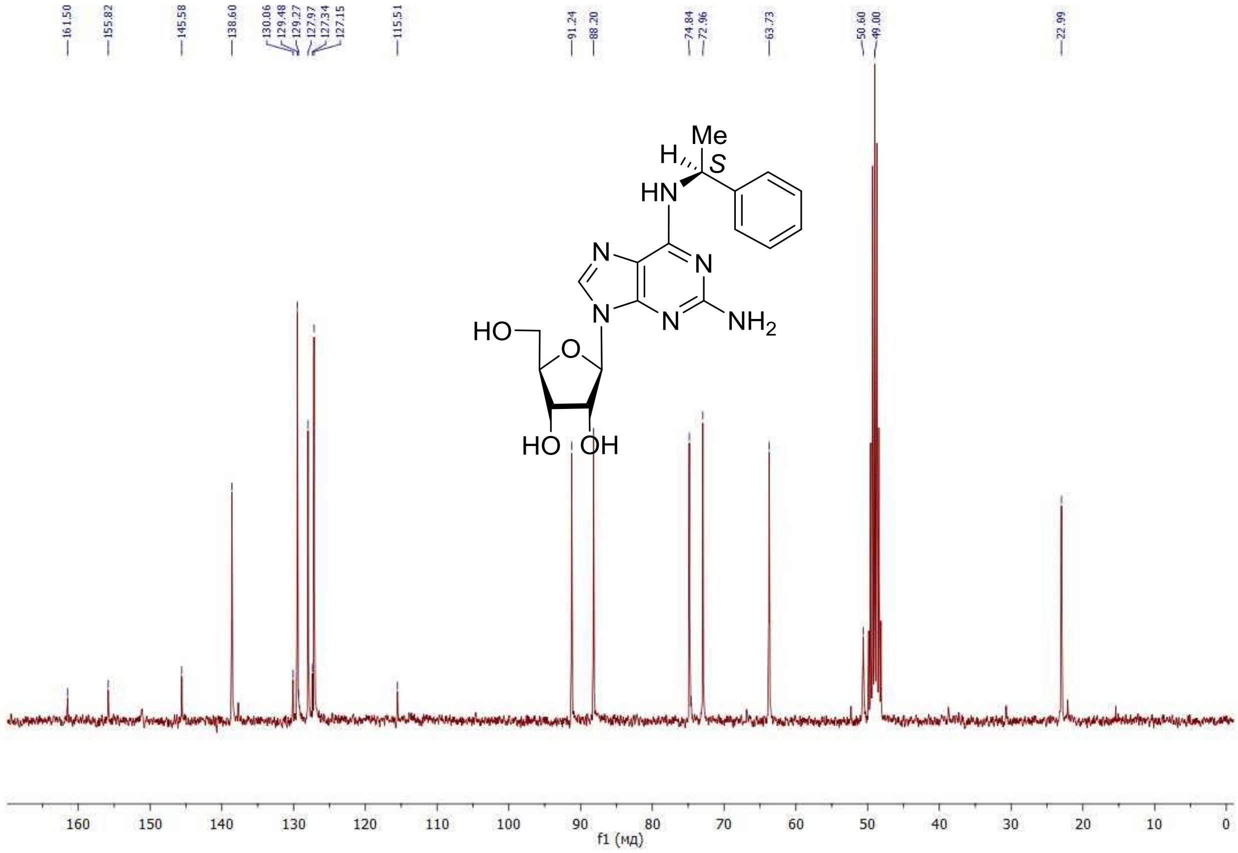

^{13}C-NMR-spectrum (75.5 MHz) of 2-amino, N^6-((S)-α-methylbenzyl)adenosine (**16b**) in CD_3OD at 303K.

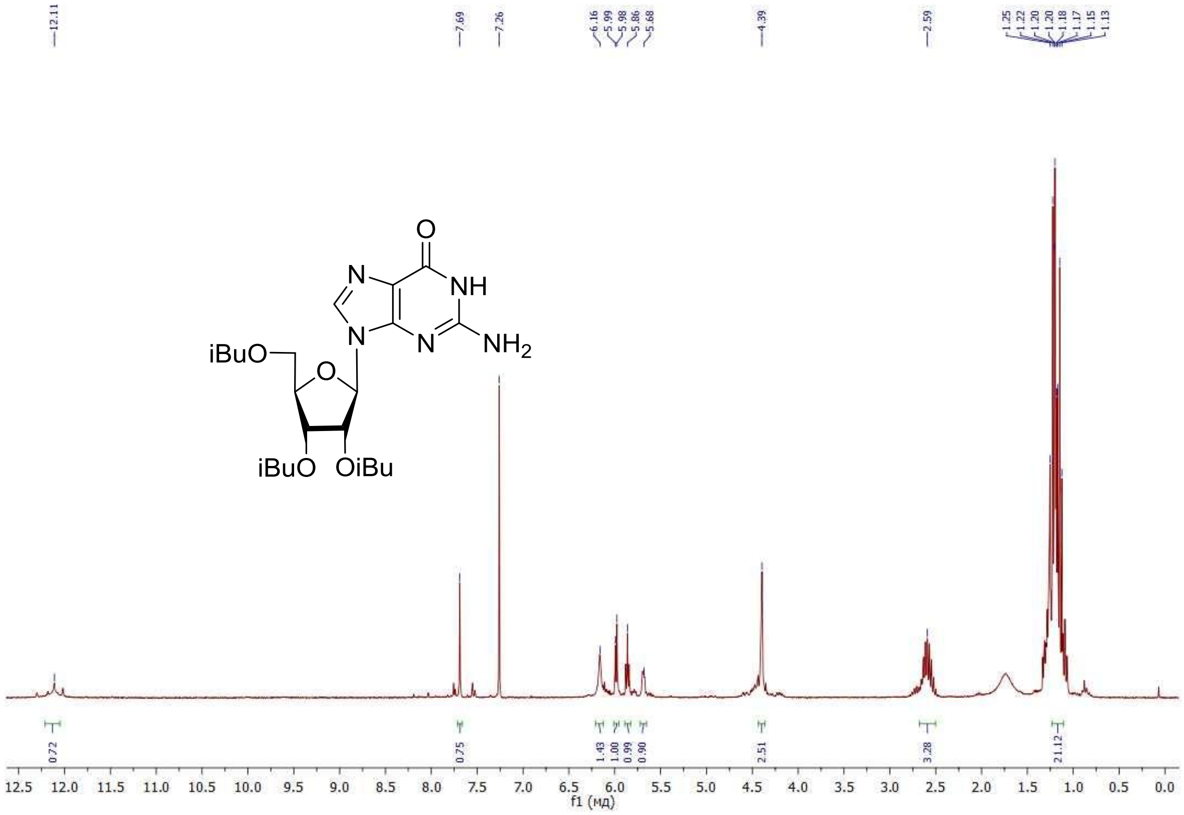

^{1}H-NMR-spectrum (300.1 MHz) of 2′,3′,5′-tri-*O*-isobutyroylguanosine (17) in $CDCl_3$ at 303K.

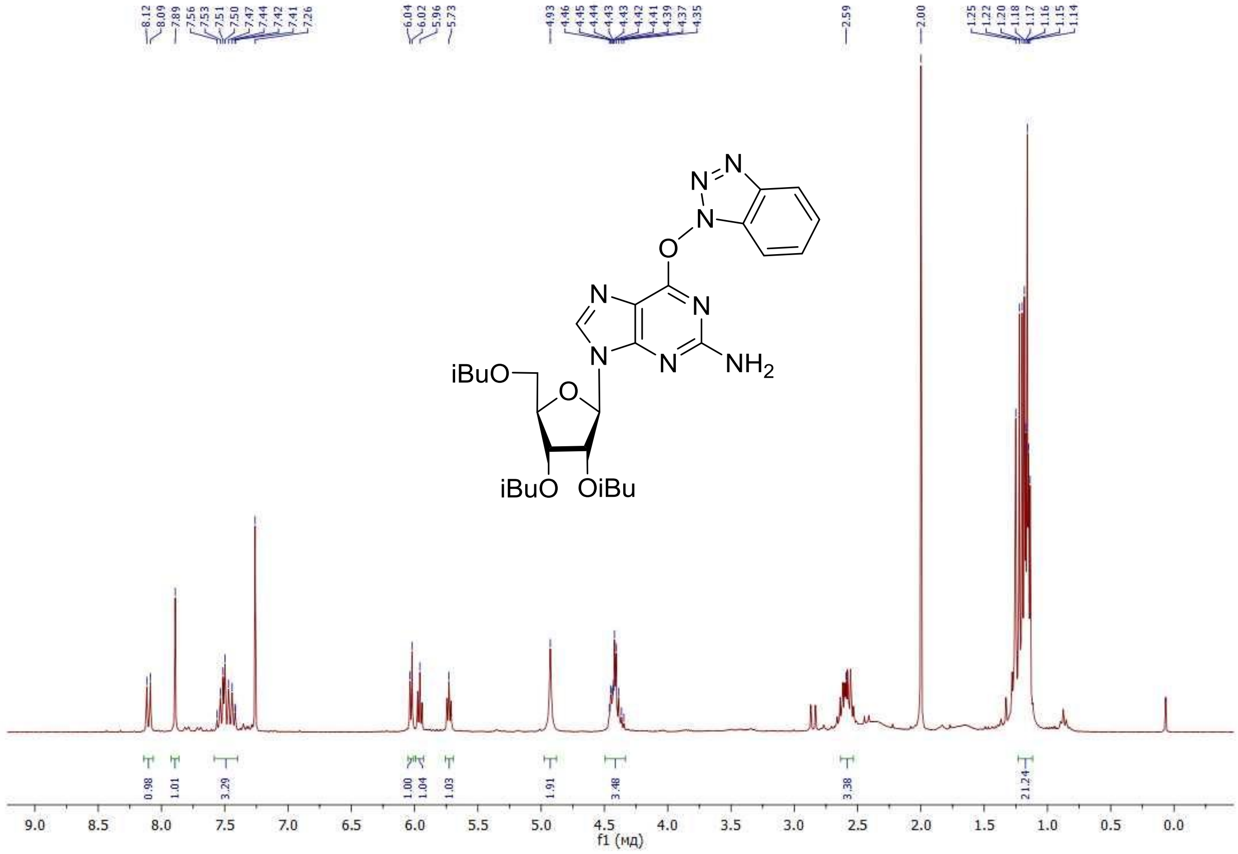

^{1}H-NMR-spectrum (300.1 MHz) of 2-amino, *O*^{6}-(benzotriazol-1-yl)-2′,3′,5′-tri-*O*-isobutyroyladenosine (**18**) in $CDCl_3$ at 303K.

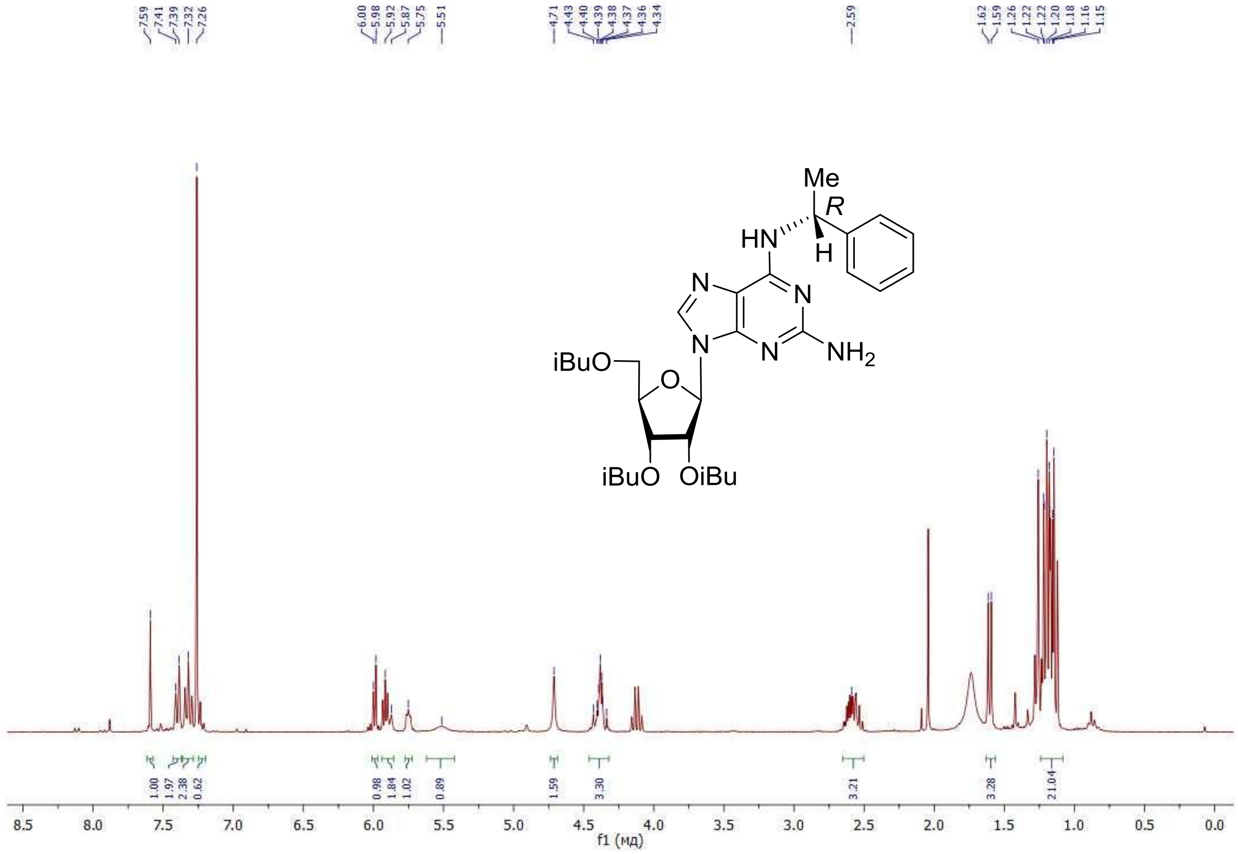

^{1}H-NMR-spectrum (300.1 MHz) of 2-amino, N^{6}-((R)-α-methylbenzyl)-2′,3′,5′-tri-*O*-isobutyroyladenosine (19) in $CDCl_3$ at 303K.

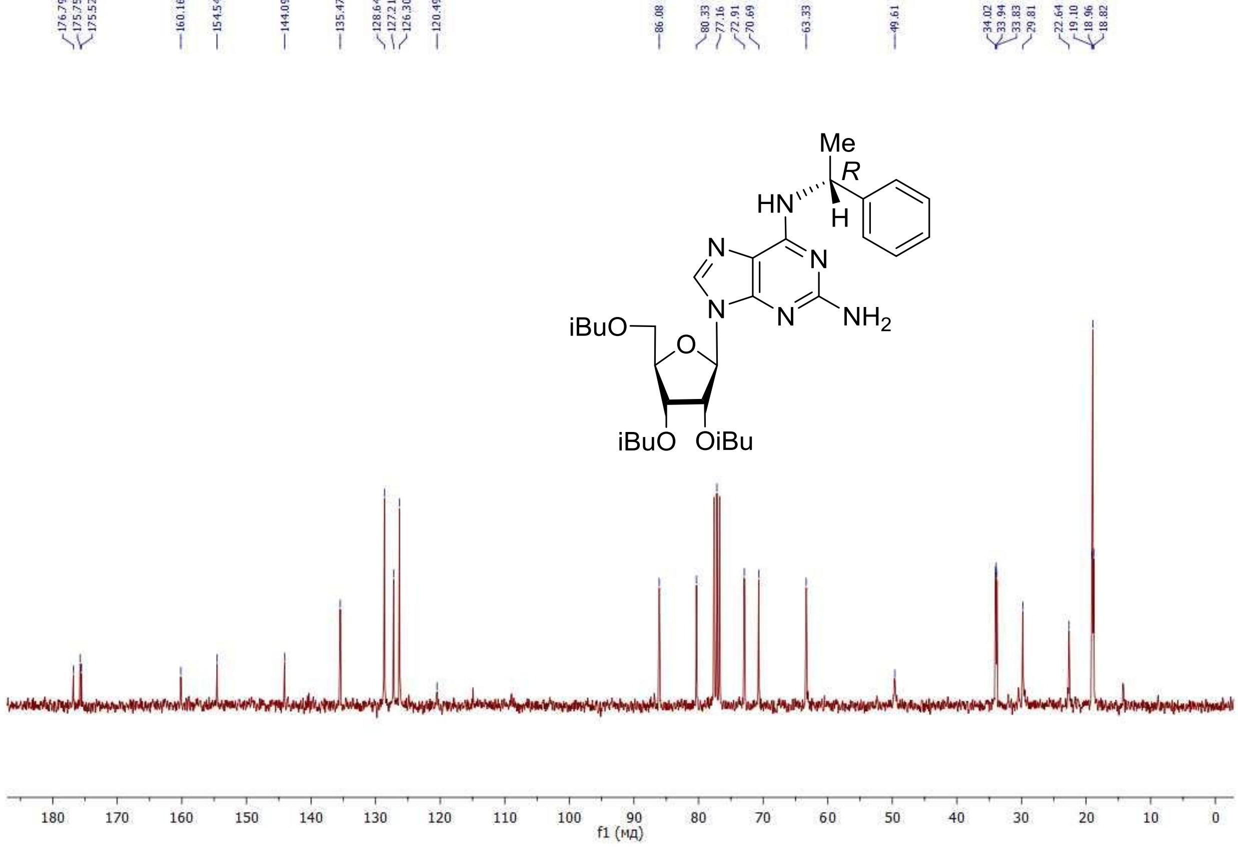

^{13}C-NMR-spectrum (75.5 MHz) of 2-amino, N^6-((R)-α-methylbenzyl)-2′,3′,5′-tri-*O*-isobutyroyladenosine (19) in $CDCl_3$ at 303K.

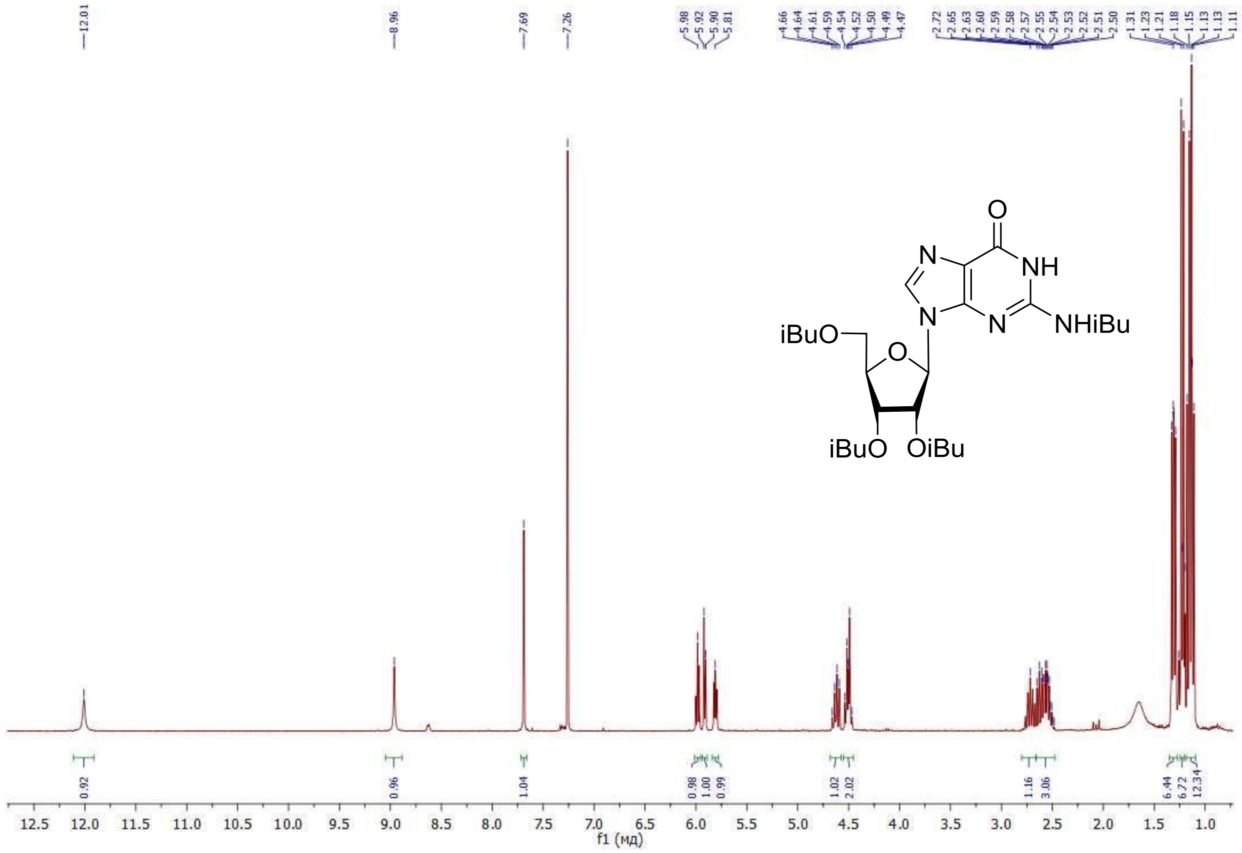

^{1}H-NMR-spectrum (300.1 MHz) of *2*-isobutyroylamino-2′,3′,5′-tri-*O*-isobutyroyladenosine (20) in $CDCl_3$ at 303K

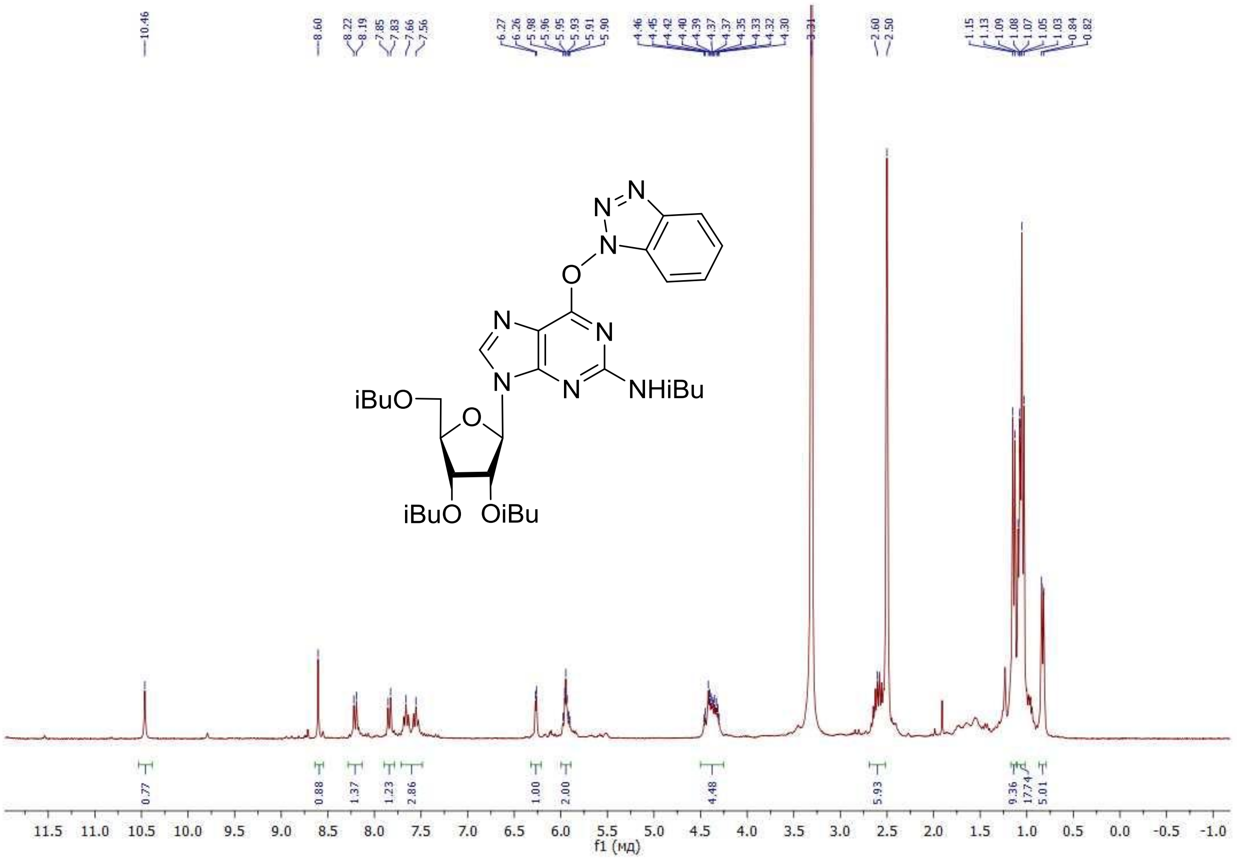

^{1}H-NMR-spectrum (300.1 MHz) of *O*[6]-(benzotriazol-1-yl)-*2*-isobutyroylamino-2′,3′,5′-tri-*O*-isobutyroyladenosine (21) in DMSO-d_6 at 303K.

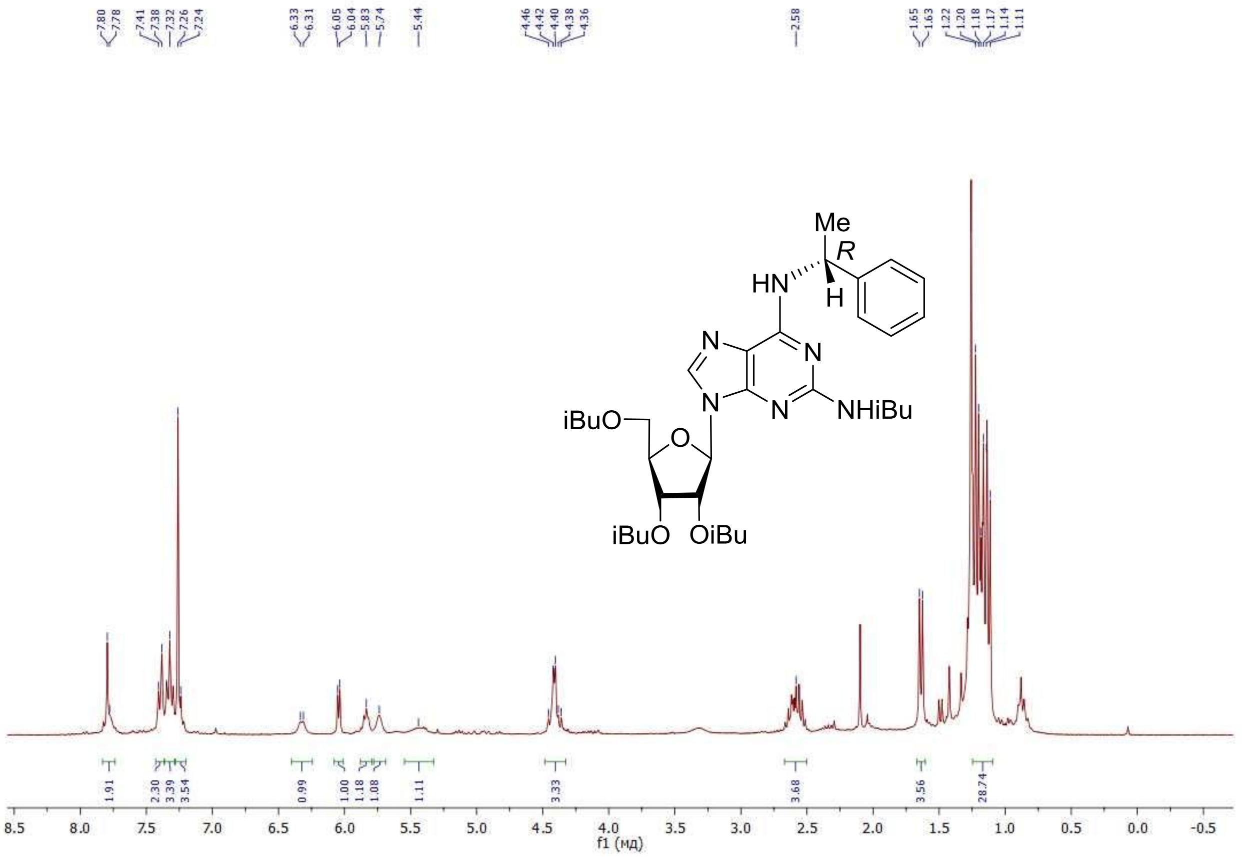

^{1}H-NMR-spectrum (300.1 MHz) of *2*-isobutyroylamino, *N*[6]-((R)-α-methylbenzyl)-2′,3′,5′-tri-*O*-isobutyroyladenosine (**22a**) inCDCl$_3$ at 303K.

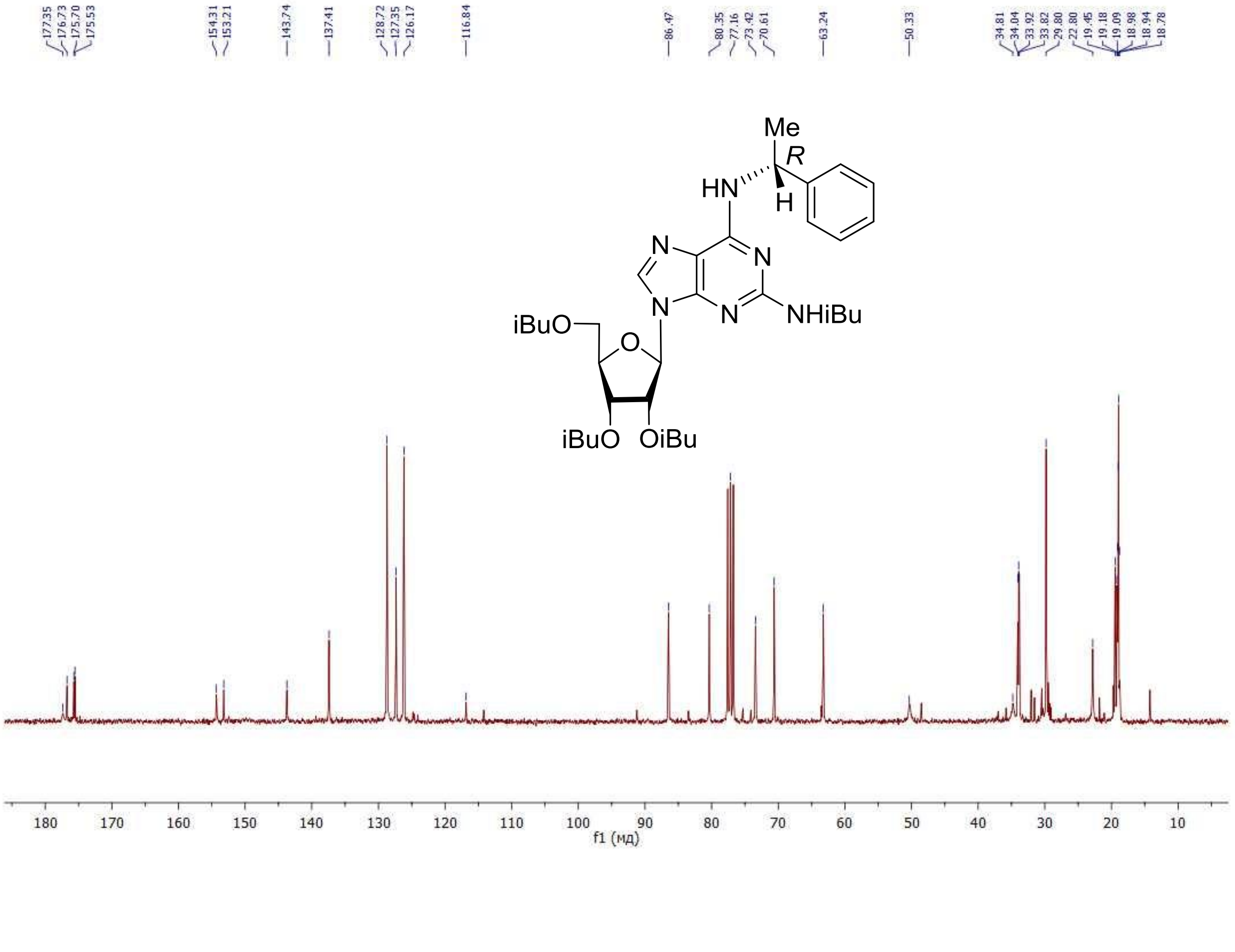

^{13}C-NMR-spectrum (75.5 MHz) of *2*-isobutyroylamino, N^6-((R)-α-methylbenzyl)-2′,3′,5′-tri-*O*-isobutyroyladenosine (**22a**) in $CDCl_3$ at 303K.

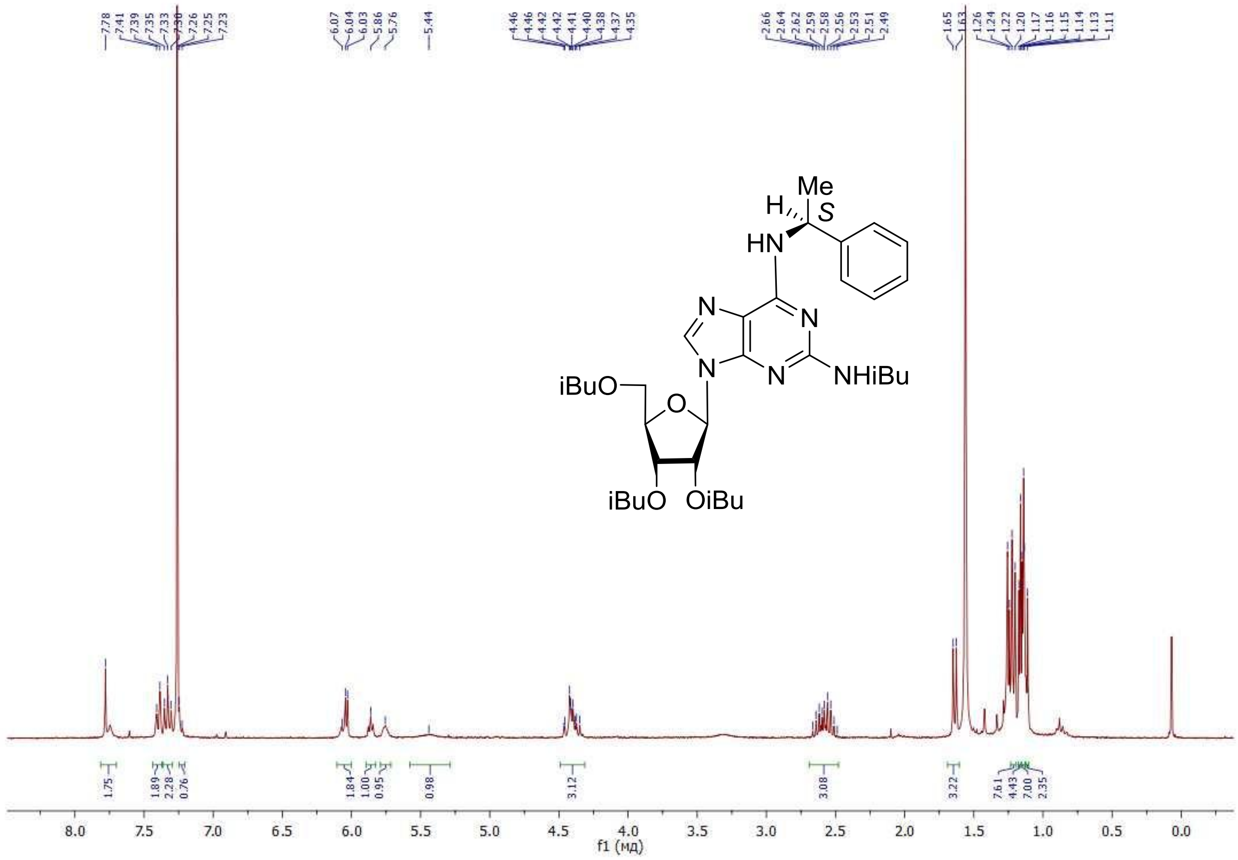

^{1}H-NMR-spectrum (300.1 MHz) of *2*-isobutyroylamino, N^6-((S)-α-methylbenzyl)-2′,3′,5′-tri-*O*-isobutyroyladenosine (**22b**) in $CDCl_3$ at 303K.

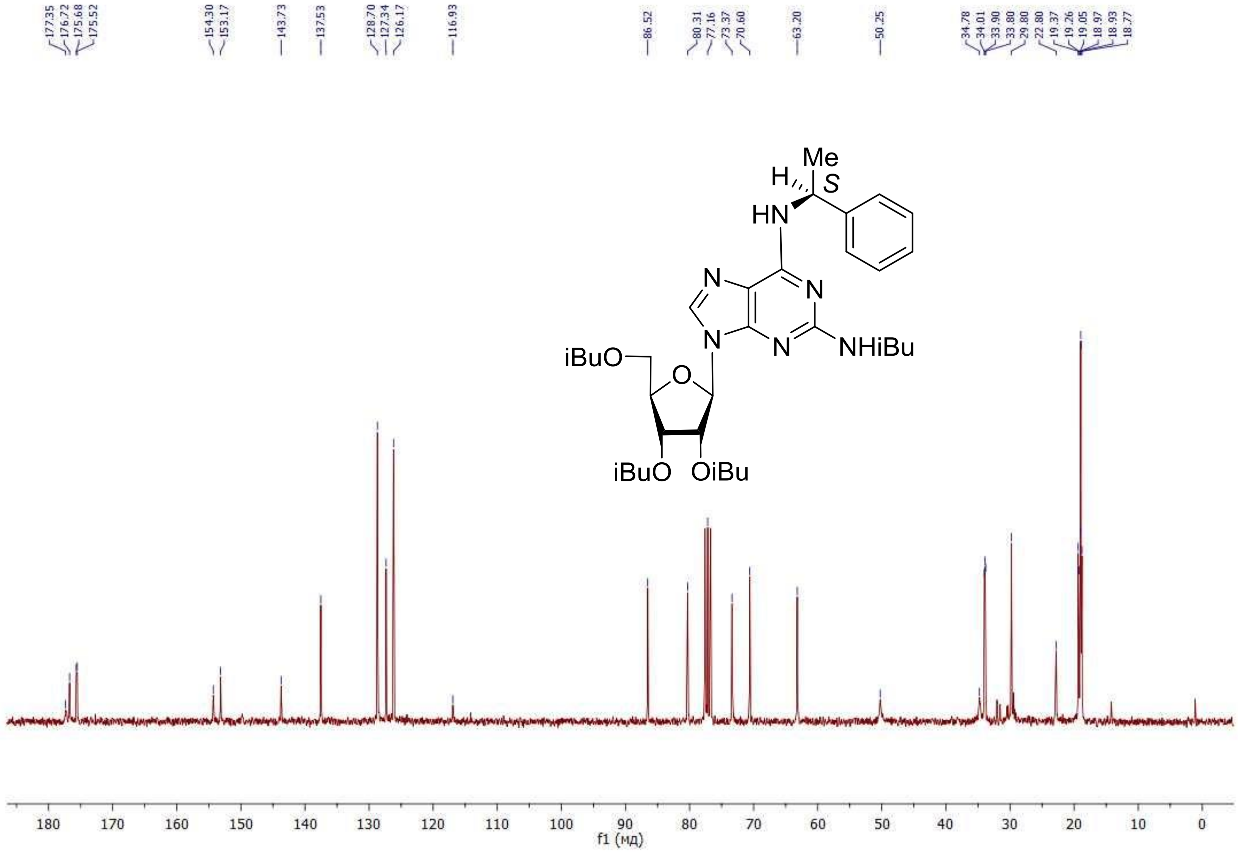

^{13}C-NMR-spectrum (75.5MHz) of2-isobutyroylamino, *N*^{6}-((S)-α-methylbenzyl)-2′,3′,5′-tri-*O*-isobutyroyladenosine (22b) in $CDCl_3$ at 303K.

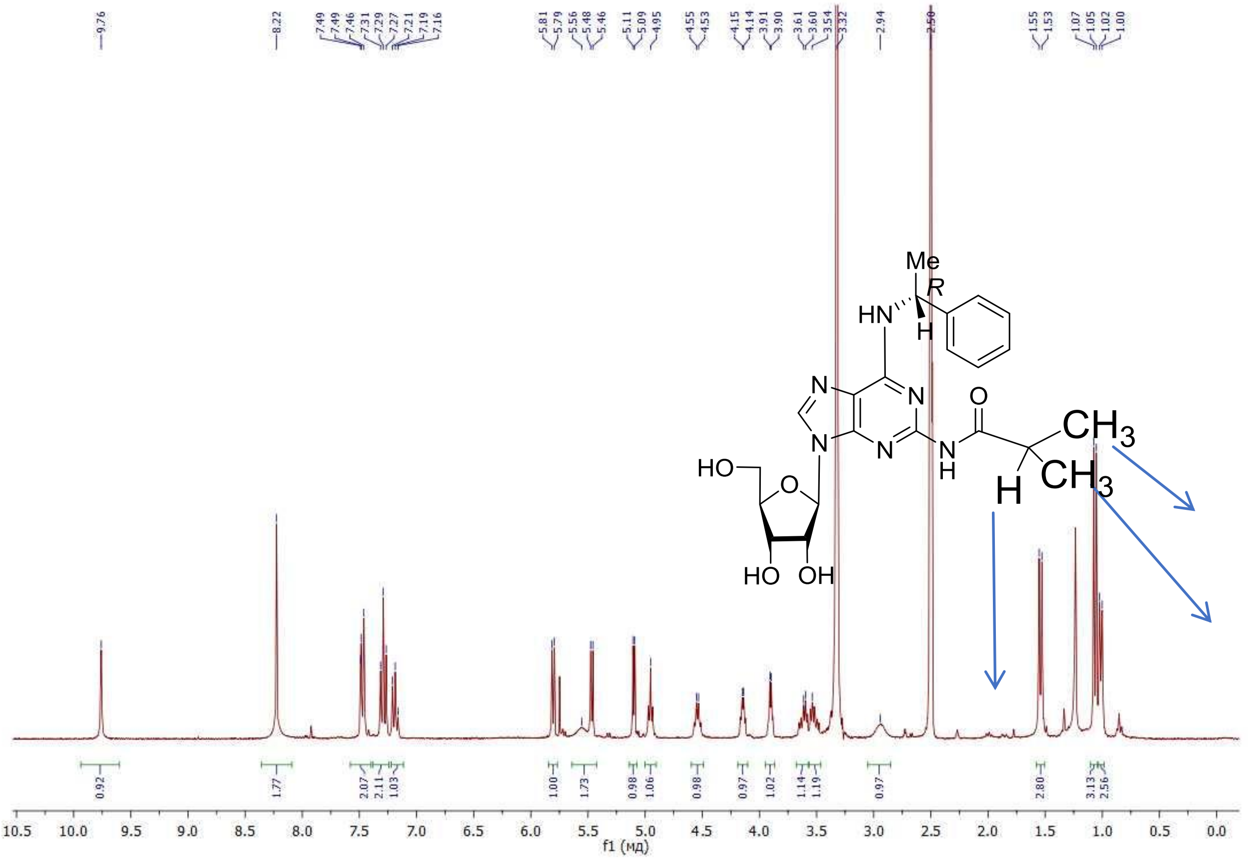

^{1}H-NMR-spectrum (300.1 MHz) of *2*-isobutyroylamino, *N*^{6}-((R)-α-methylbenzyl)adenosine (**23a)** in DMSO-*d*$_{6}$ at 303K

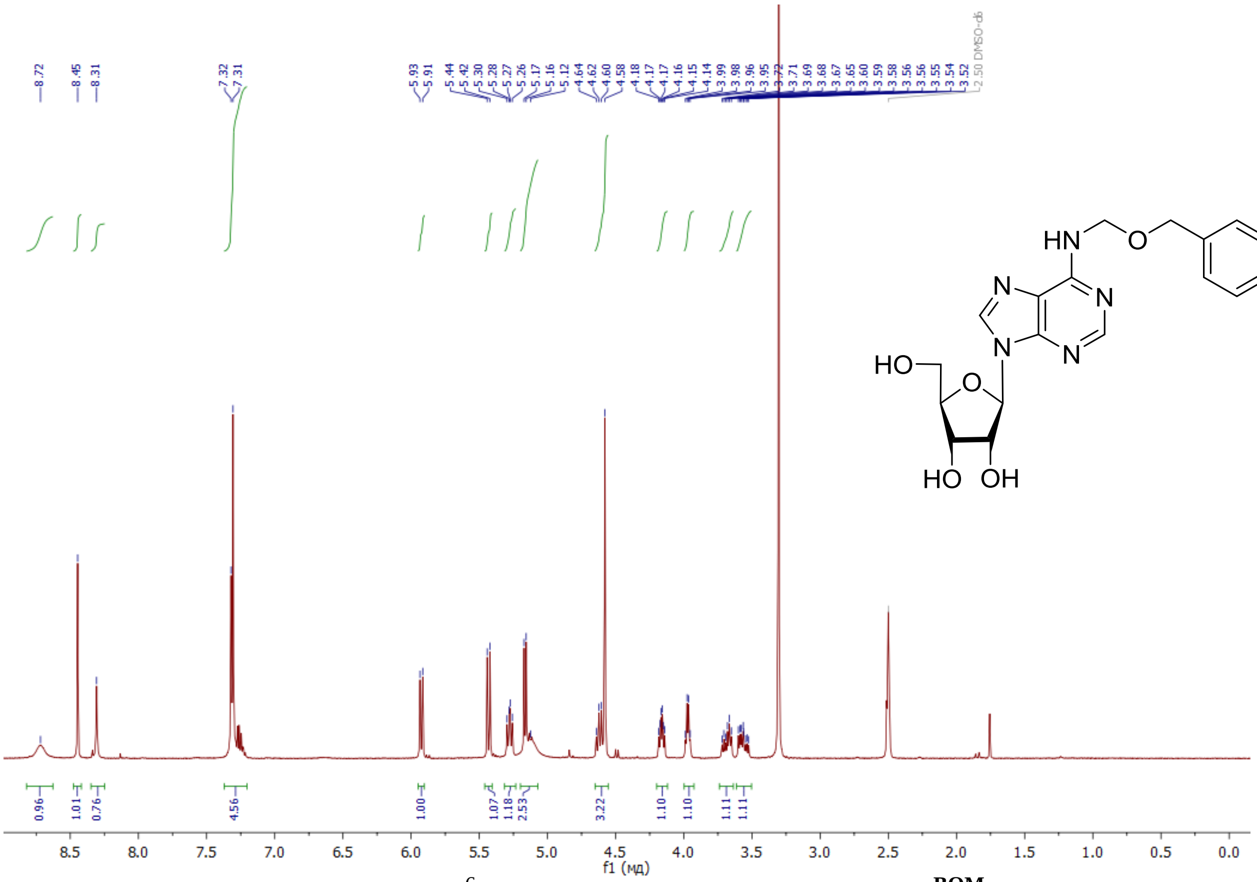

^{1}H-NMR-spectrum (300.1 MHz) of N^6-(benzyloxymethyl)adenosine (**Ado$^{\mathbf{BOM}}$**) in DMSO-d_6 at 303K

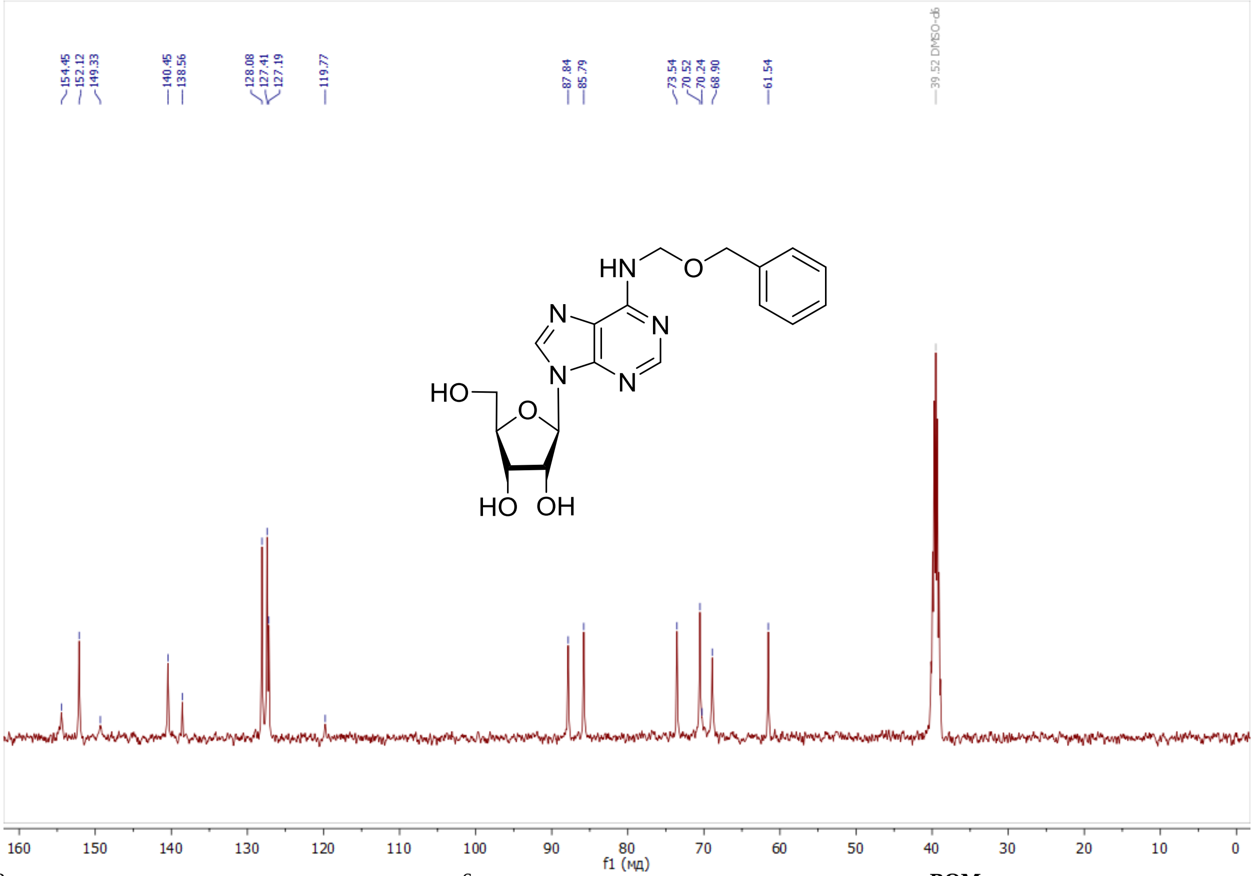

^{13}C-NMR-spectrum (75.5 MHz) of N^6-(benzyloxymethyl)adenosine (**Ado$^{\mathbf{BOM}}$**) in DMSO-d_6 at 303K

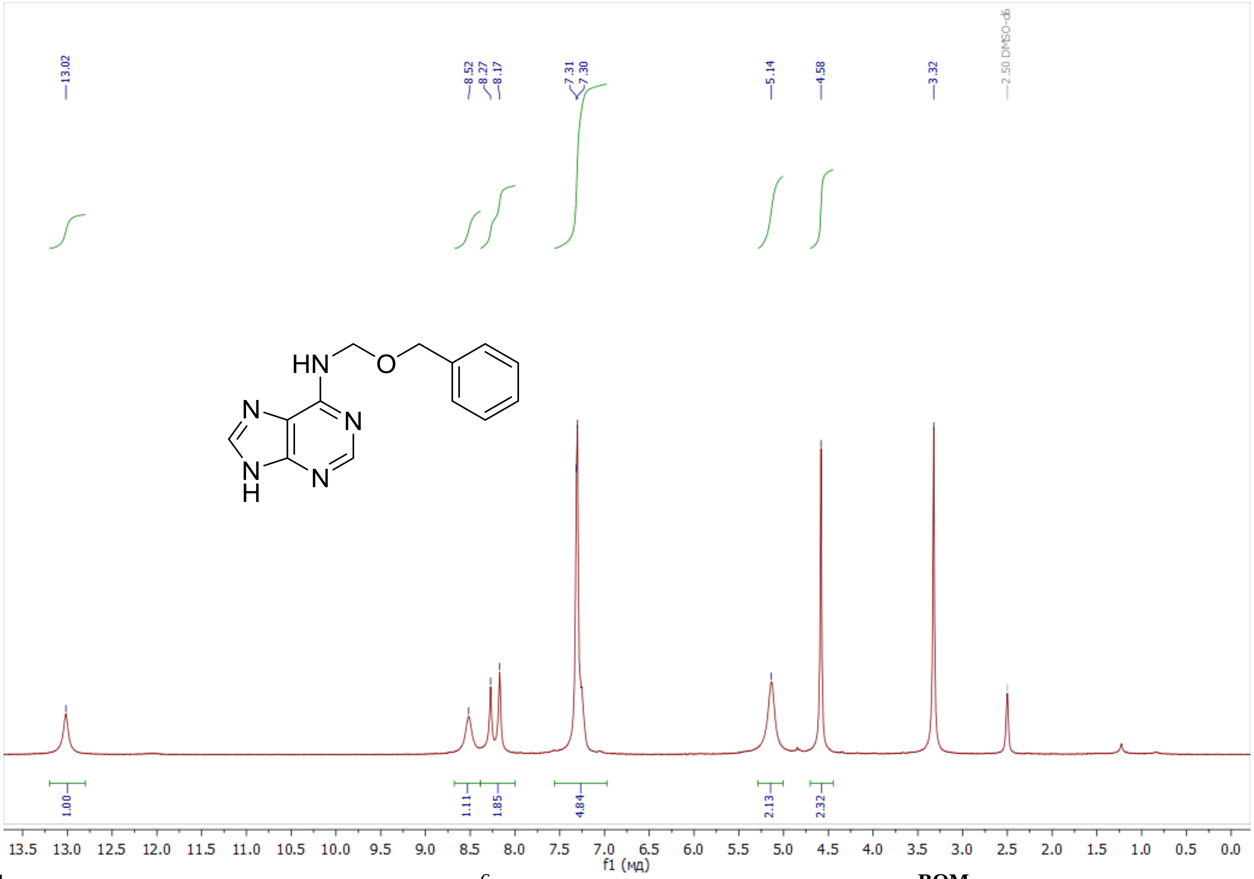

^{1}H-NMR-spectrum (300.1 MHz) of N^{6}-(benzyloxymethyl)adenine (**Ade^{BOM}**) in DMSO-d_6 at 303K

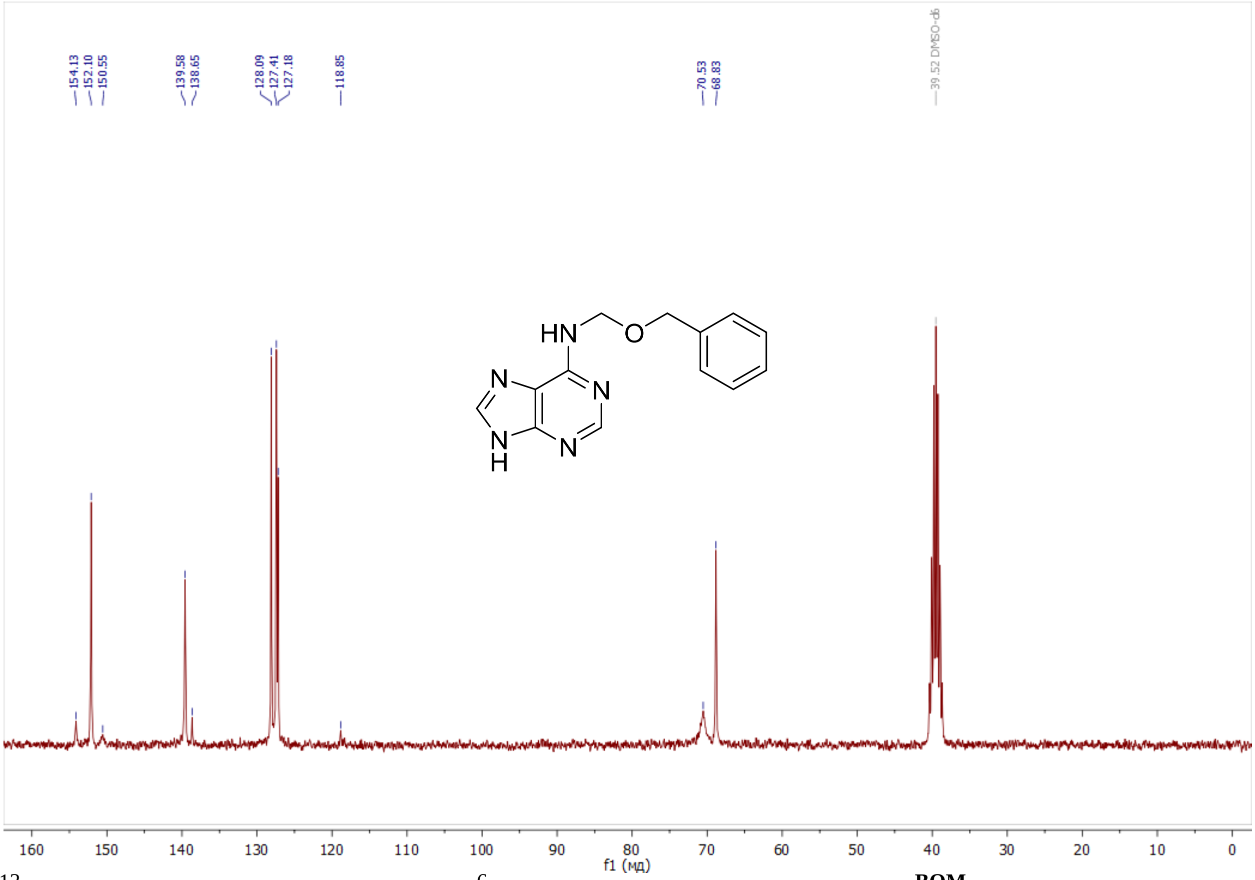

^{13}C-NMR-spectrum (75.5 MHz) of N^{6}-(benzyloxymethyl)adenine (**Ade^{BOM}**) in DMSO-d_6 at 303K

4. High resolution mass spectrometry (HRMS)

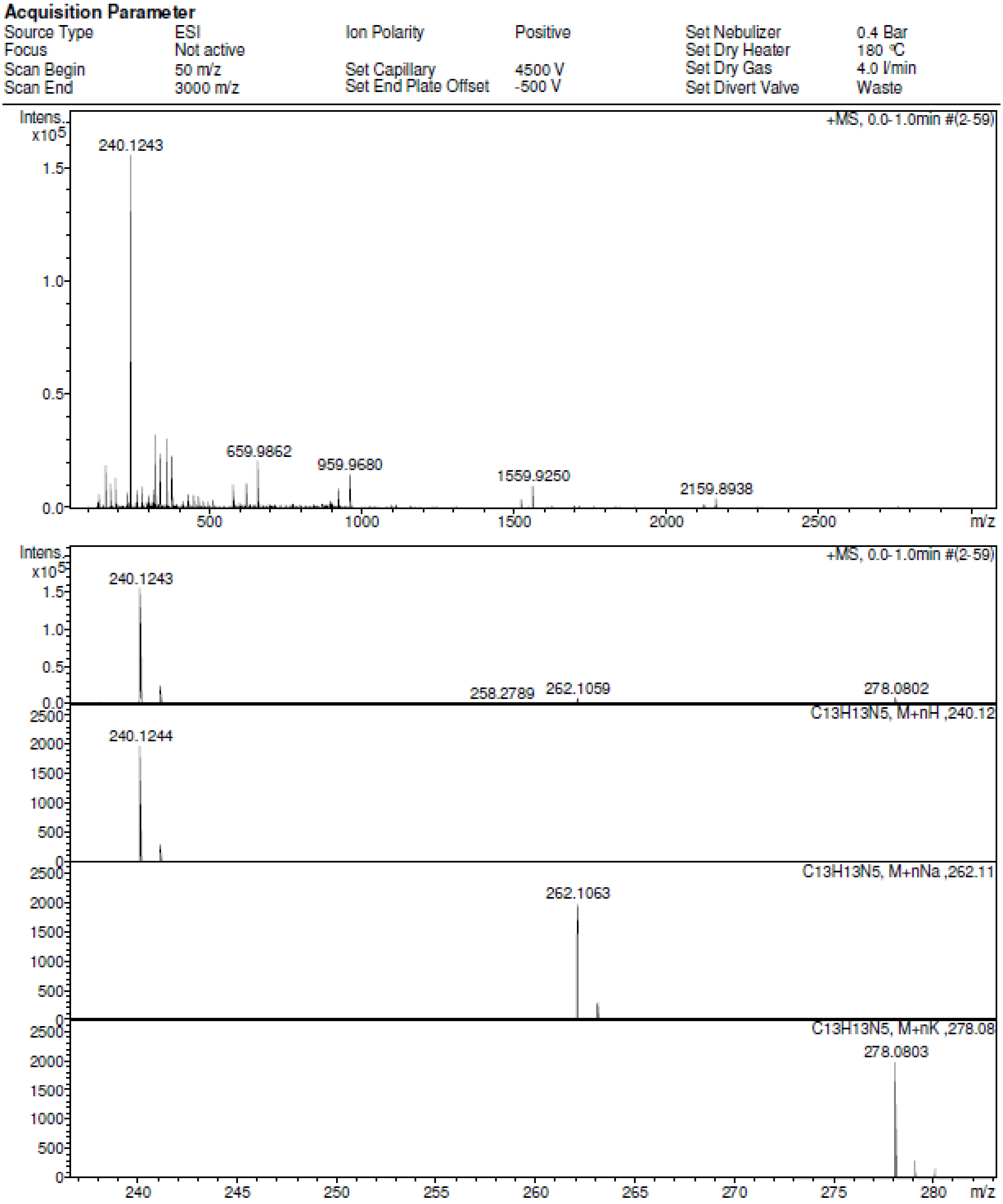

High-resolution mass spectrum (HRMS) of *N*^{6}-((R)-α-methylbenzyl)adenine (**5a**)

Acquisition Parameter

Source Type	ESI	Ion Polarity	Positive	Set Nebulizer	0.4 Bar
Focus	Not active			Set Dry Heater	180 °C
Scan Begin	50 m/z	Set Capillary	4500 V	Set Dry Gas	4.0 l/min
Scan End	3000 m/z	Set End Plate Offset	-500 V	Set Divert Valve	Waste

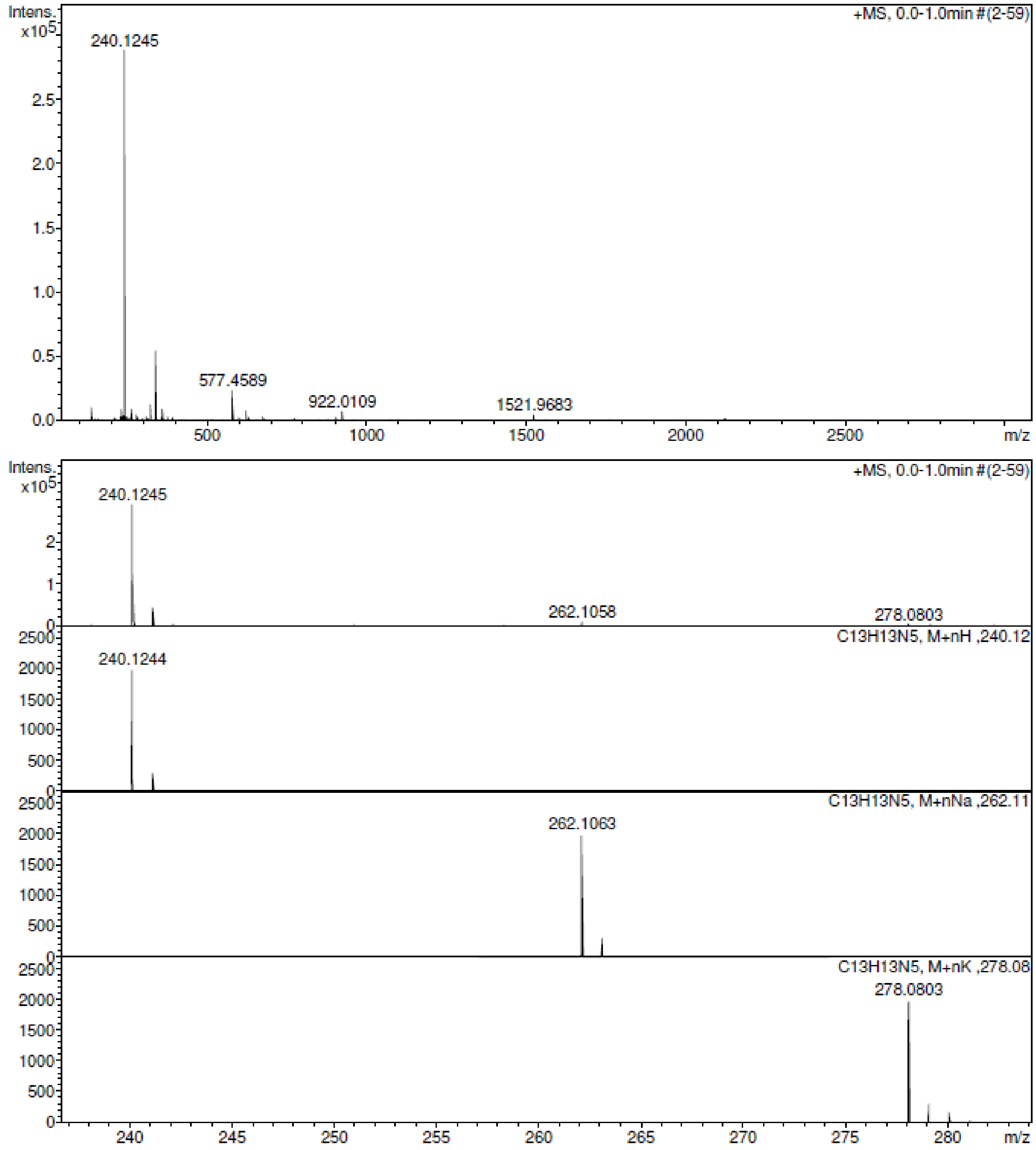

High-resolution mass spectrum (HRMS) of N^6-((S)-α-methylbenzyl)adenine (**5b**)

Acquisition Parameter

Source Type	ESI	Ion Polarity	Positive	Set Nebulizer	0.4 Bar
Focus	Not active			Set Dry Heater	180 °C
Scan Begin	50 m/z	Set Capillary	4500 V	Set Dry Gas	4.0 l/min
Scan End	1550 m/z	Set End Plate Offset	-500 V	Set Divert Valve	Source

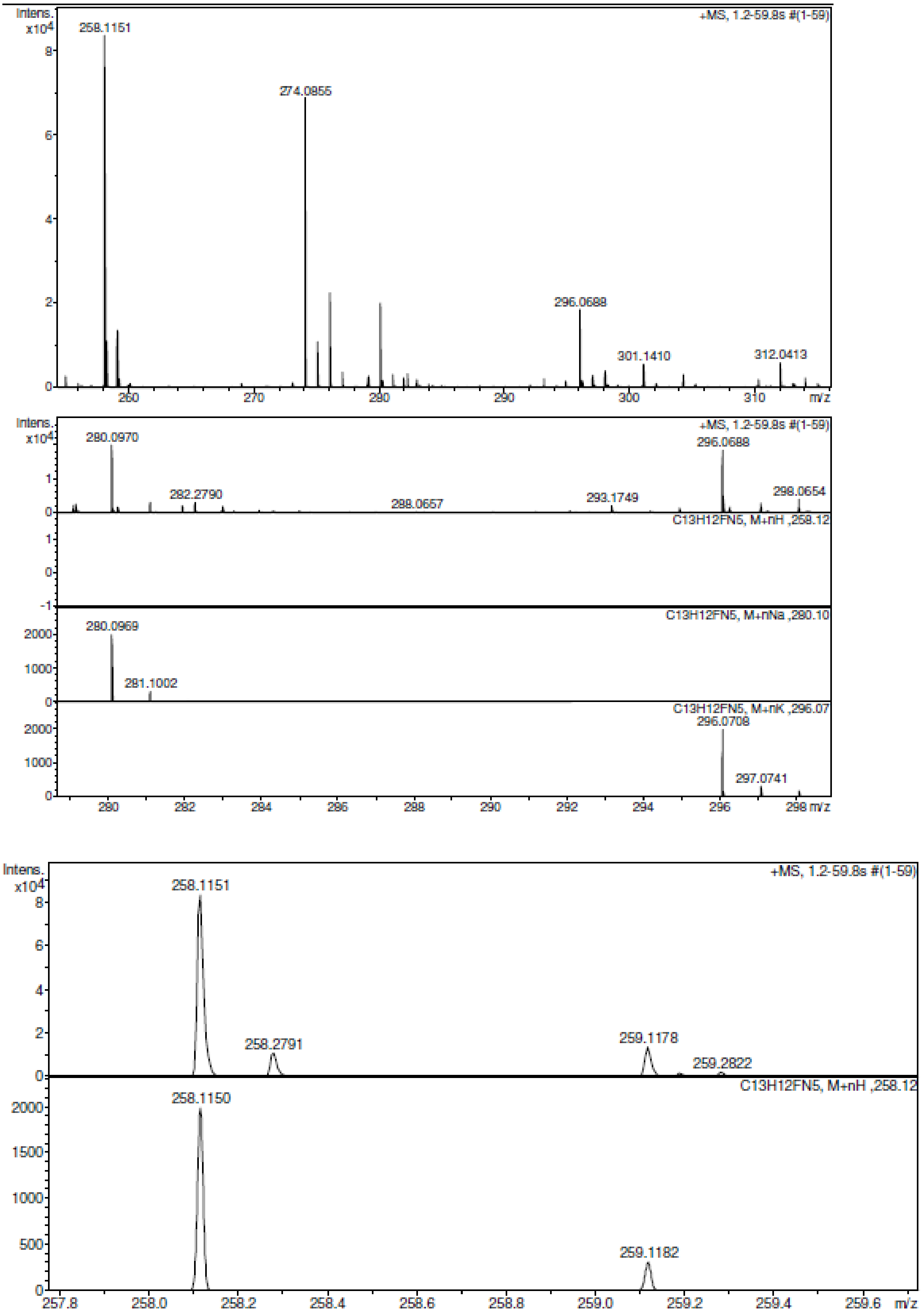

High-resolution mass spectrum (HRMS) of 2-fluoro, N^6-((R)-α-methylbenzyl)adenine (**6a**)

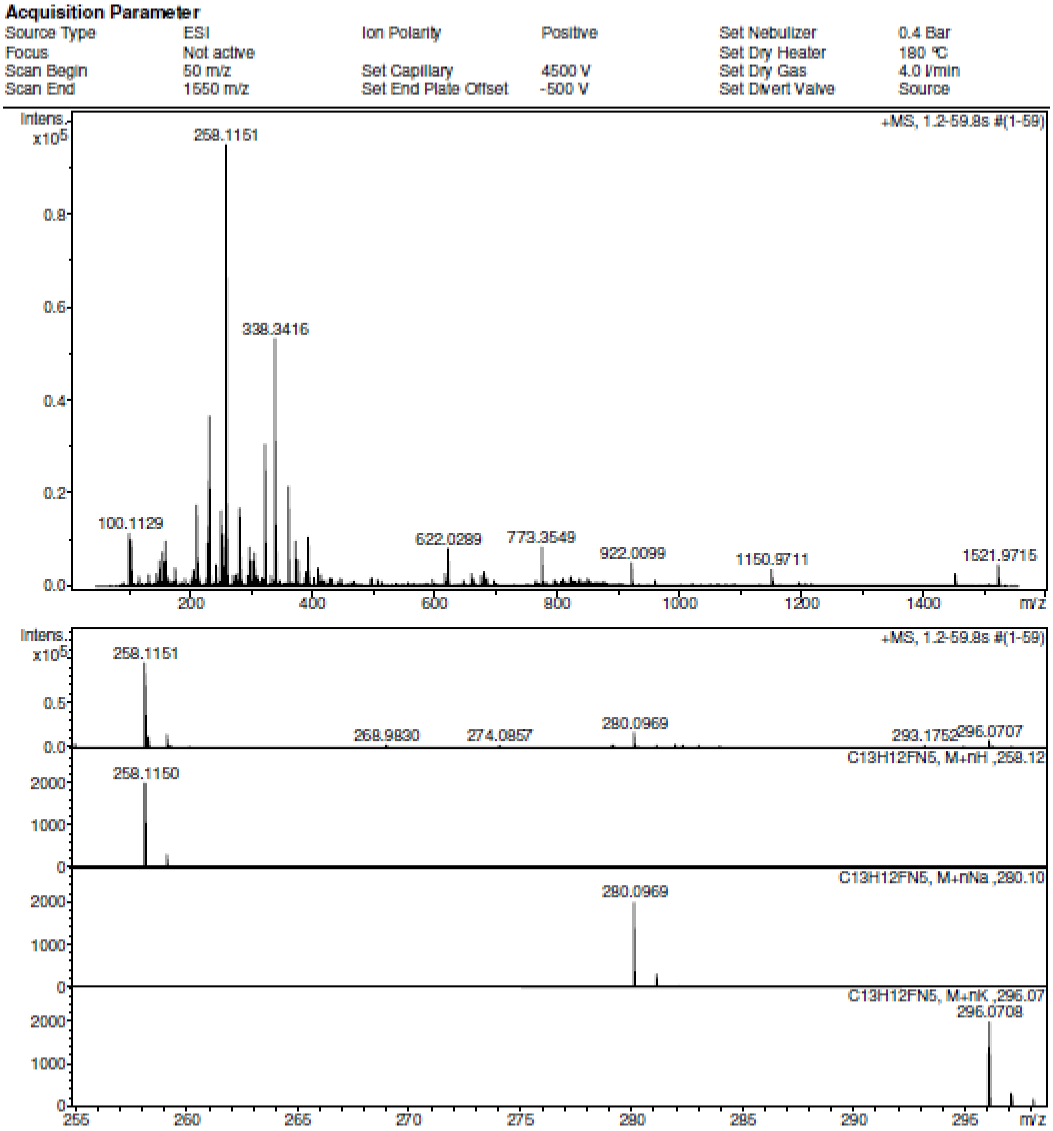

High-resolution mass spectrum (HRMS) of 2-fluoro, N^6-((S)-α-methylbenzyl)adenine (**6b**)

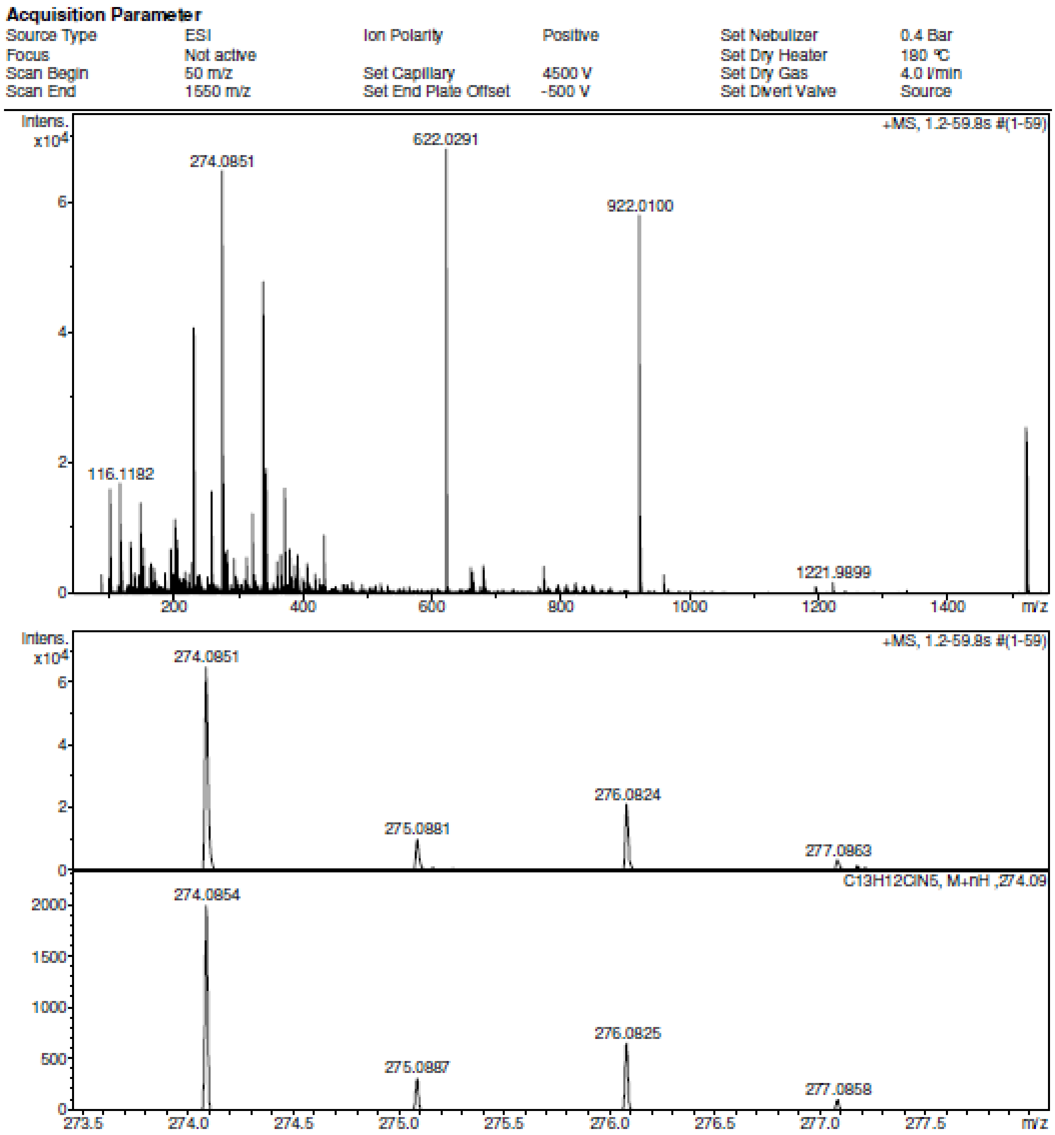

High-resolution mass spectrum (HRMS) of 2-chloro, *N*^{6}-((R)-α-methylbenzyl)adenine (**7a**)

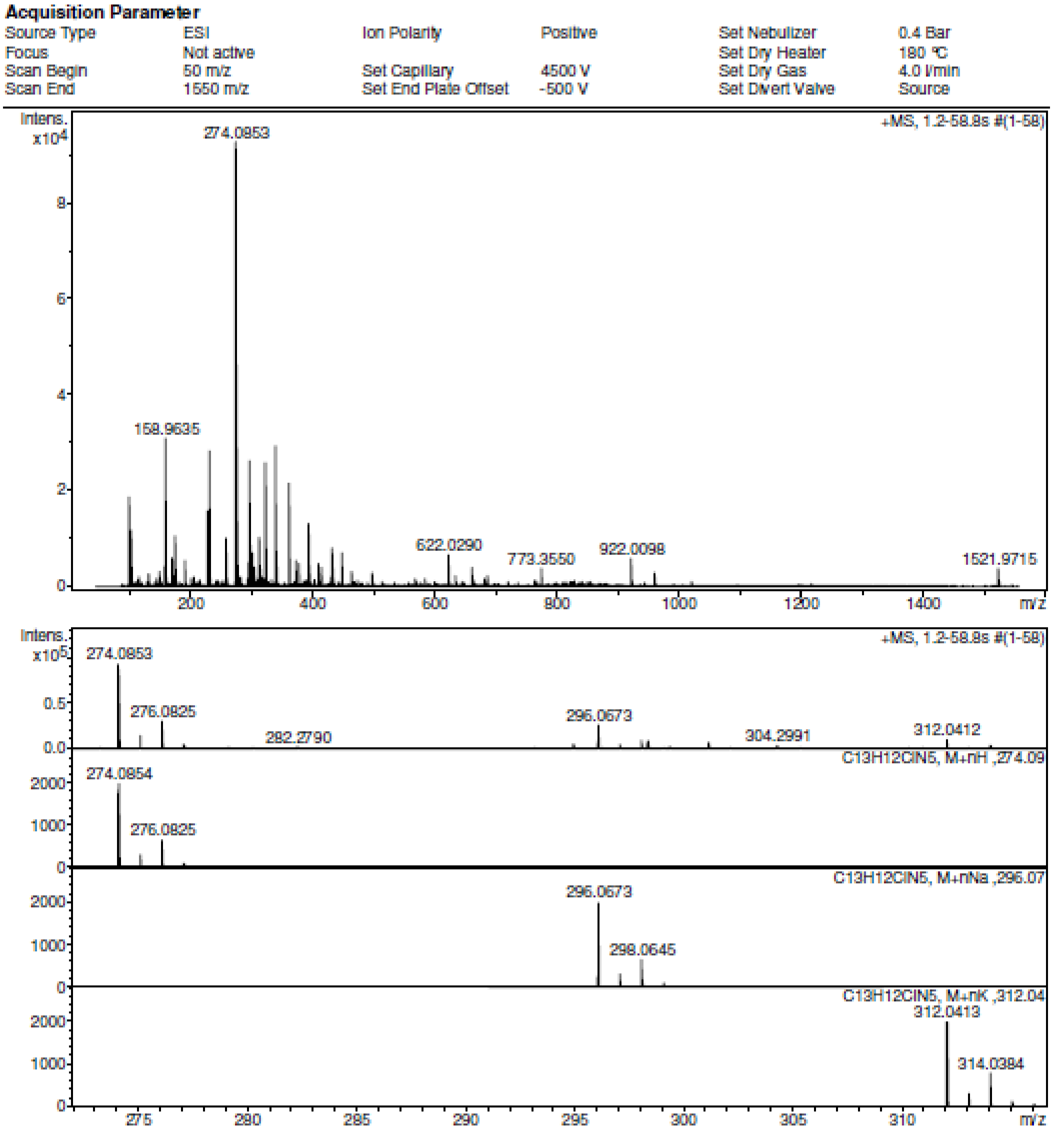

High-resolution mass spectrum (HRMS) of 2-chloro, N^6-((S)-α-methylbenzyl)adenine (**7b**)

Acquisition Parameter

Source Type	ESI	Ion Polarity	Positive	Set Nebulizer	0.4 Bar
Focus	Not active			Set Dry Heater	180 °C
Scan Begin	50 m/z	Set Capillary	4500 V	Set Dry Gas	4.0 l/min
Scan End	3000 m/z	Set End Plate Offset	-500 V	Set Divert Valve	Waste

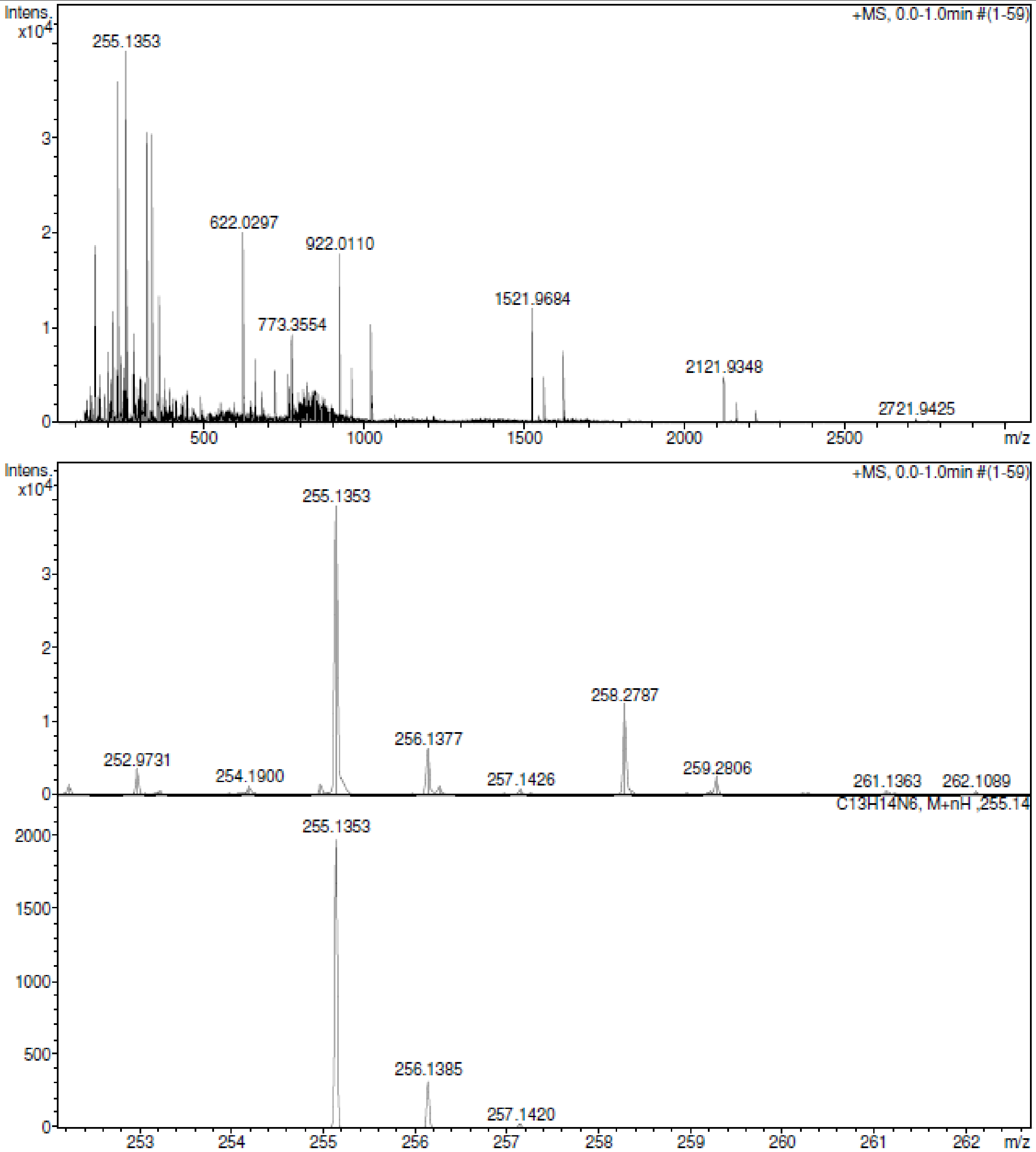

High-resolution mass spectrum (HRMS) of 2-amino, N^6-((R)-α-methylbenzyl)adenine **(8a)**

Acquisition Parameter

Source Type	ESI	Ion Polarity	Positive	Set Nebulizer	0.4 Bar
Focus	Not active			Set Dry Heater	180 °C
Scan Begin	50 m/z	Set Capillary	4500 V	Set Dry Gas	4.0 l/min
Scan End	3000 m/z	Set End Plate Offset	-500 V	Set Divert Valve	Waste

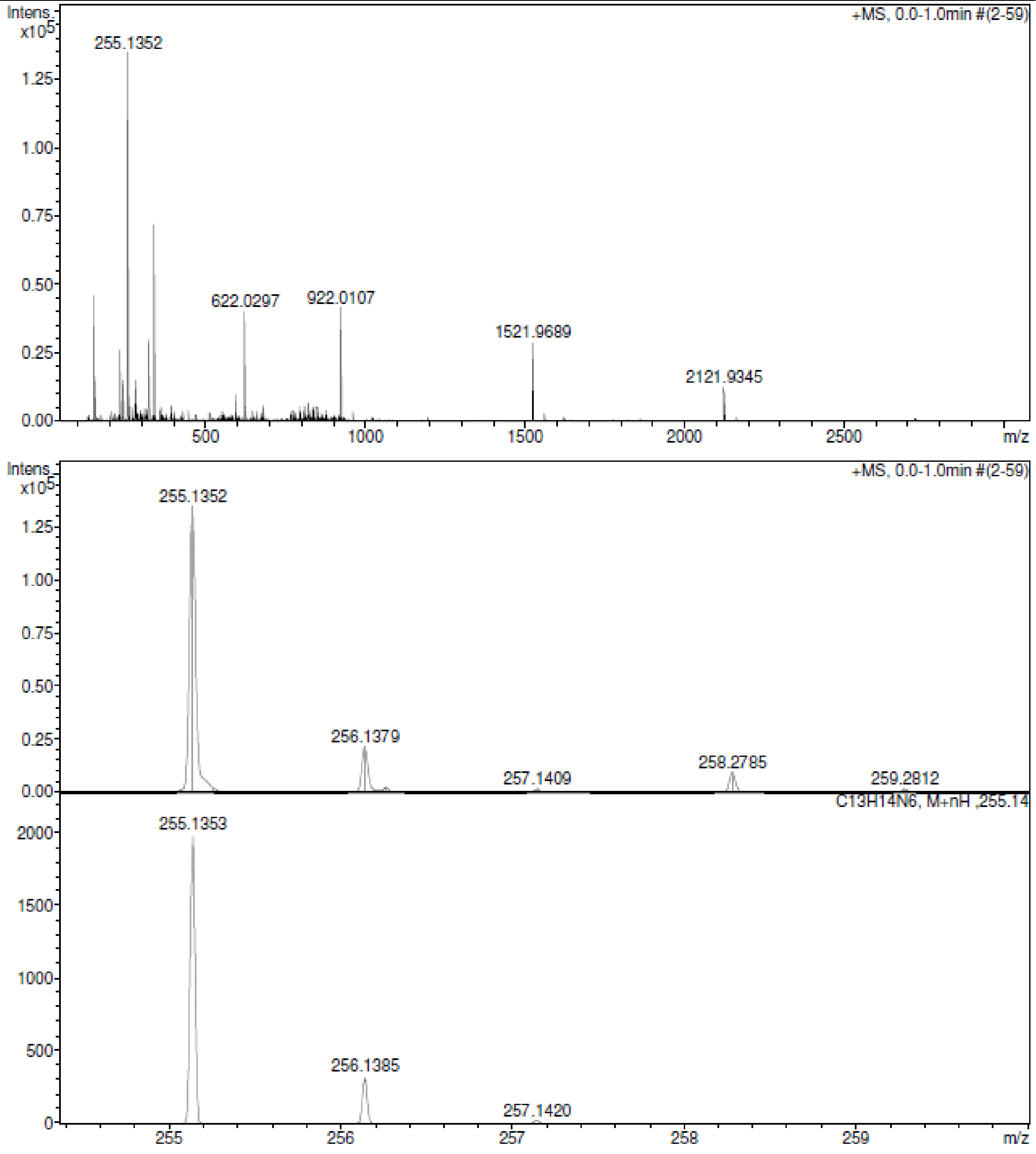

High-resolution mass spectrum (HRMS) of 2-amino, N^6-((S)-α-methylbenzyl)adenine (8b)

Acquisition Parameter

Source Type	ESI	Ion Polarity	Positive	Set Nebulizer	0.4 Bar
Focus	Not active			Set Dry Heater	180 °C
Scan Begin	50 m/z	Set Capillary	4500 V	Set Dry Gas	4.0 l/min
Scan End	3000 m/z	Set End Plate Offset	-500 V	Set Divert Valve	Waste

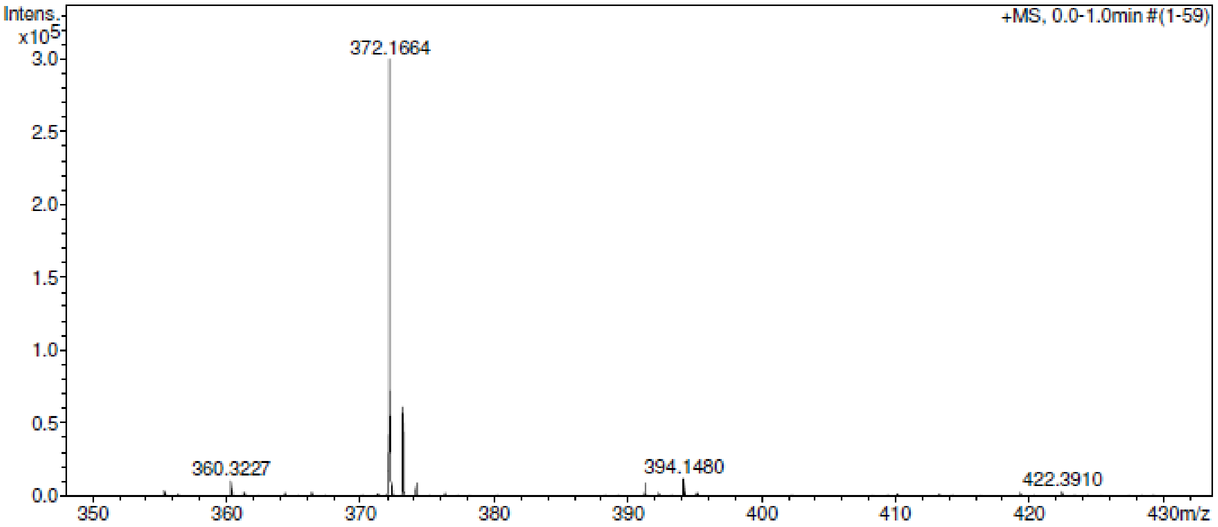

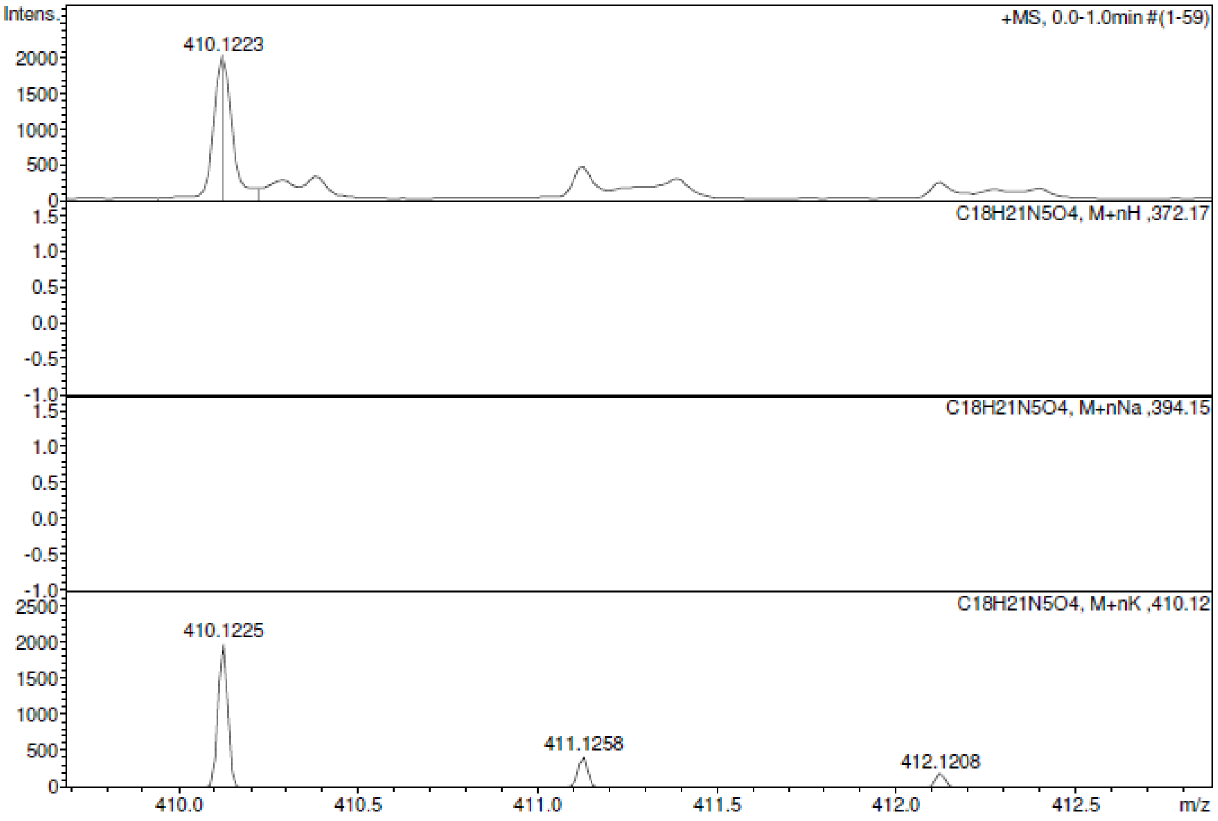

Acquisition Parameter

Source Type	ESI	Ion Polarity	Positive	Set Nebulizer	0.4 Bar
Focus	Not active			Set Dry Heater	180 °C
Scan Begin	50 m/z	Set Capillary	4500 V	Set Dry Gas	4.0 l/min
Scan End	3000 m/z	Set End Plate Offset	-500 V	Set Divert Valve	Waste

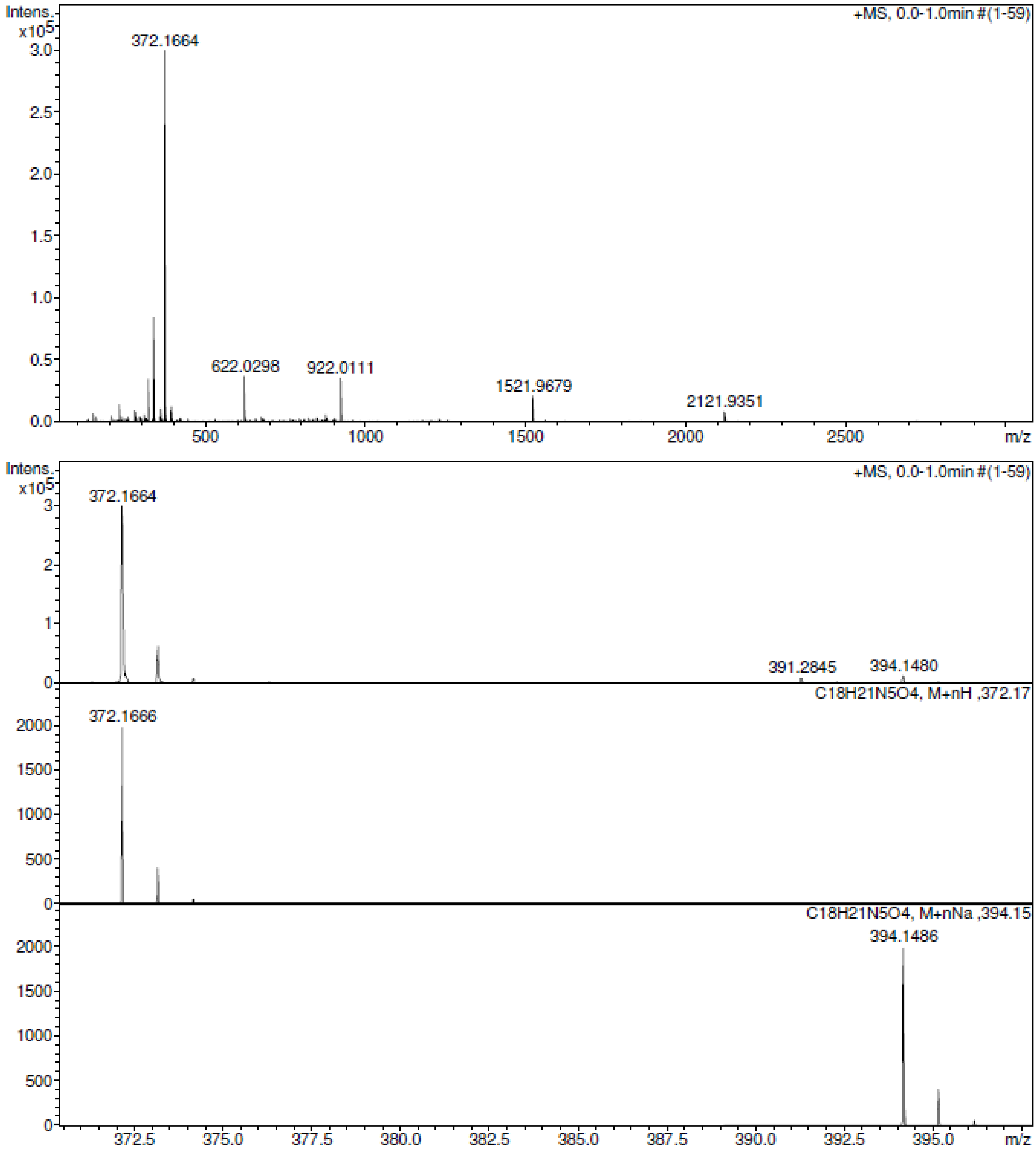

High-resolution mass spectrum (HRMS) of N^6-((R)-α-methylbenzyl)adenosine (**12a**)

Acquisition Parameter

Source Type	ESI	Ion Polarity	Positive	Set Nebulizer	0.4 Bar
Focus	Not active			Set Dry Heater	180 °C
Scan Begin	50 m/z	Set Capillary	4500 V	Set Dry Gas	4.0 l/min
Scan End	3000 m/z	Set End Plate Offset	-500 V	Set Divert Valve	Waste

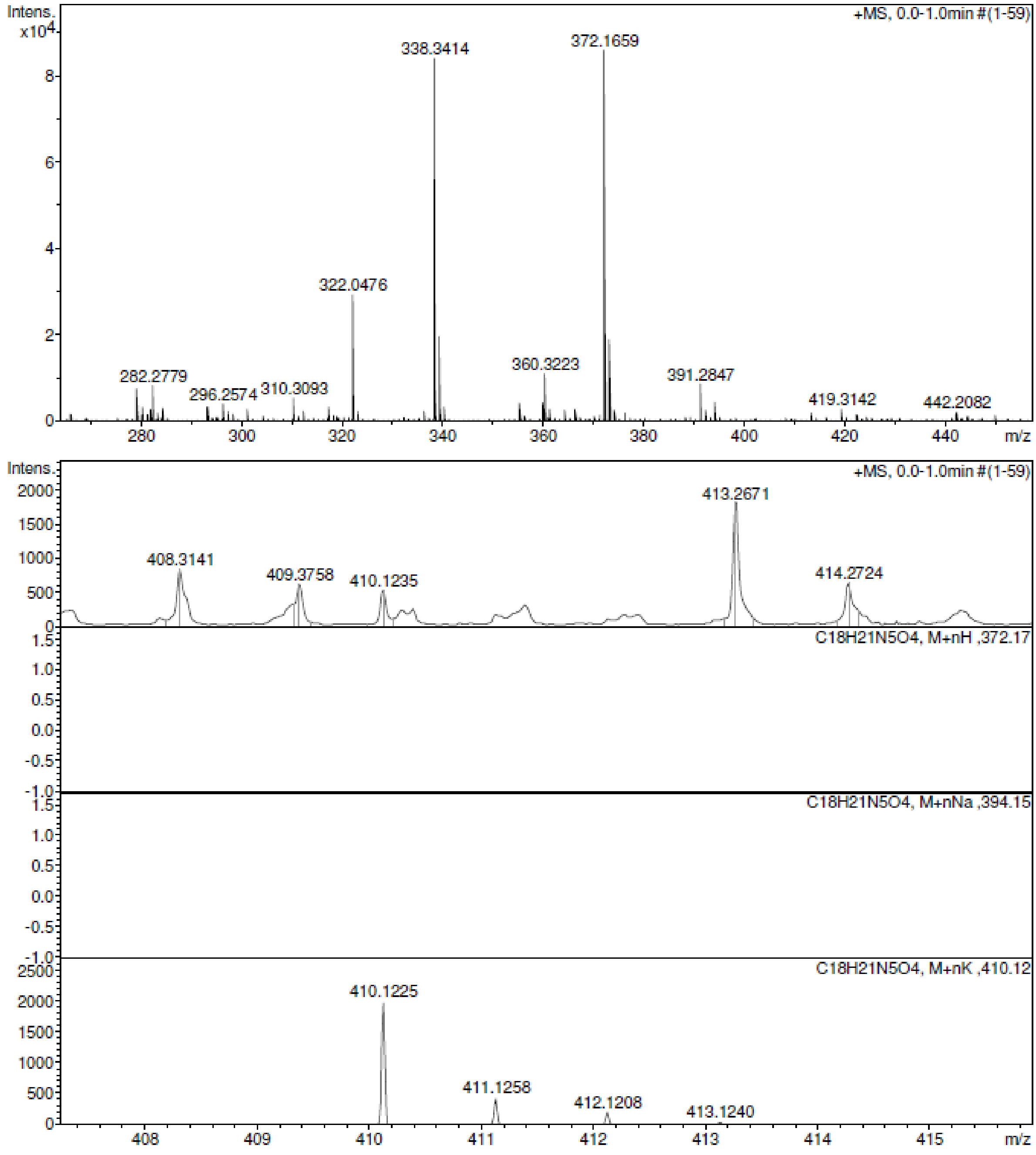

Acquisition Parameter

Source Type	ESI	Ion Polarity	Positive	Set Nebulizer	0.4 Bar
Focus	Not active			Set Dry Heater	180 °C
Scan Begin	50 m/z	Set Capillary	4500 V	Set Dry Gas	4.0 l/min
Scan End	3000 m/z	Set End Plate Offset	-500 V	Set Divert Valve	Waste

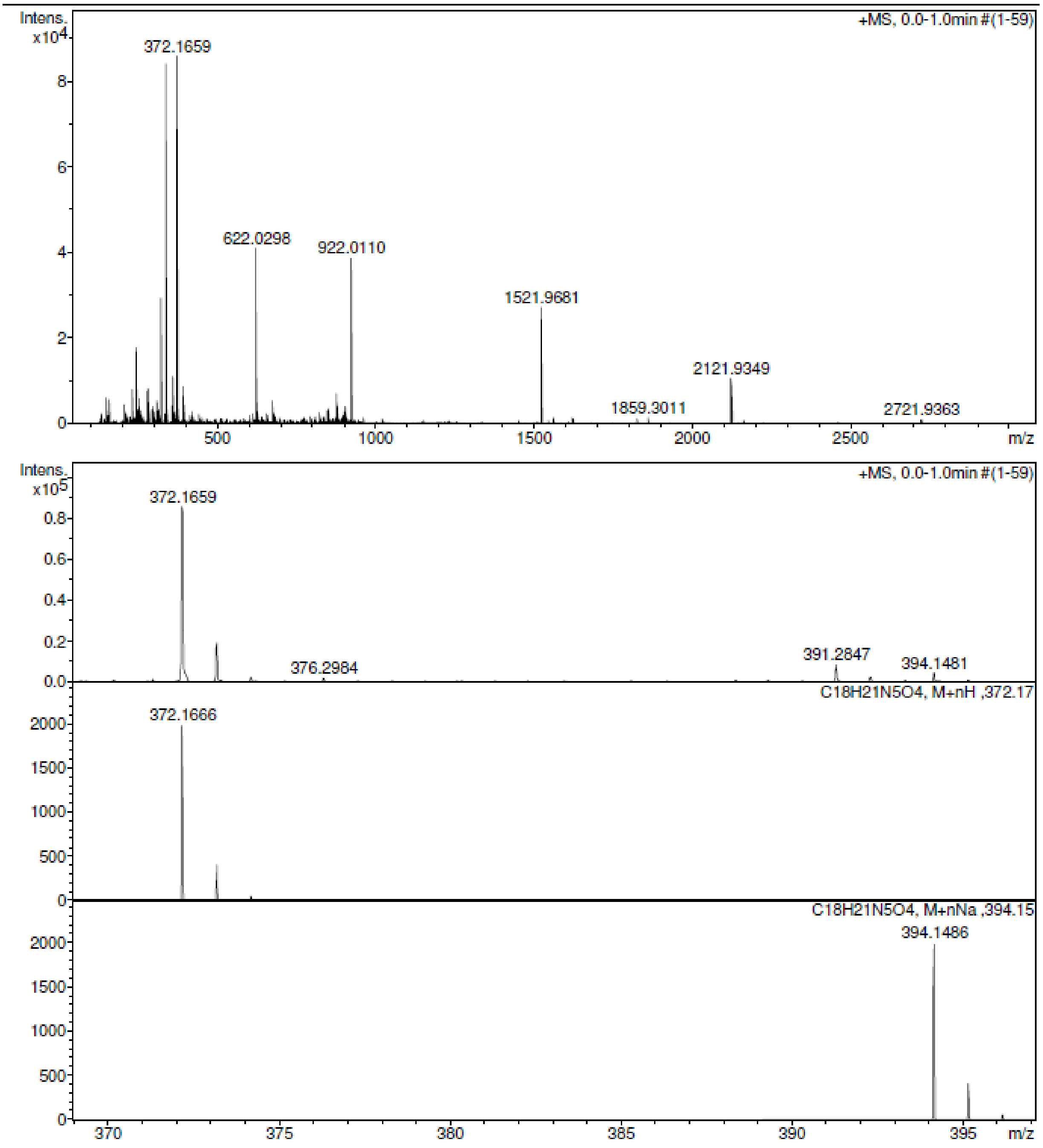

HRMS-spectrum ofN^6-((S)-α-methylbenzyl)adenosine (**12b**)

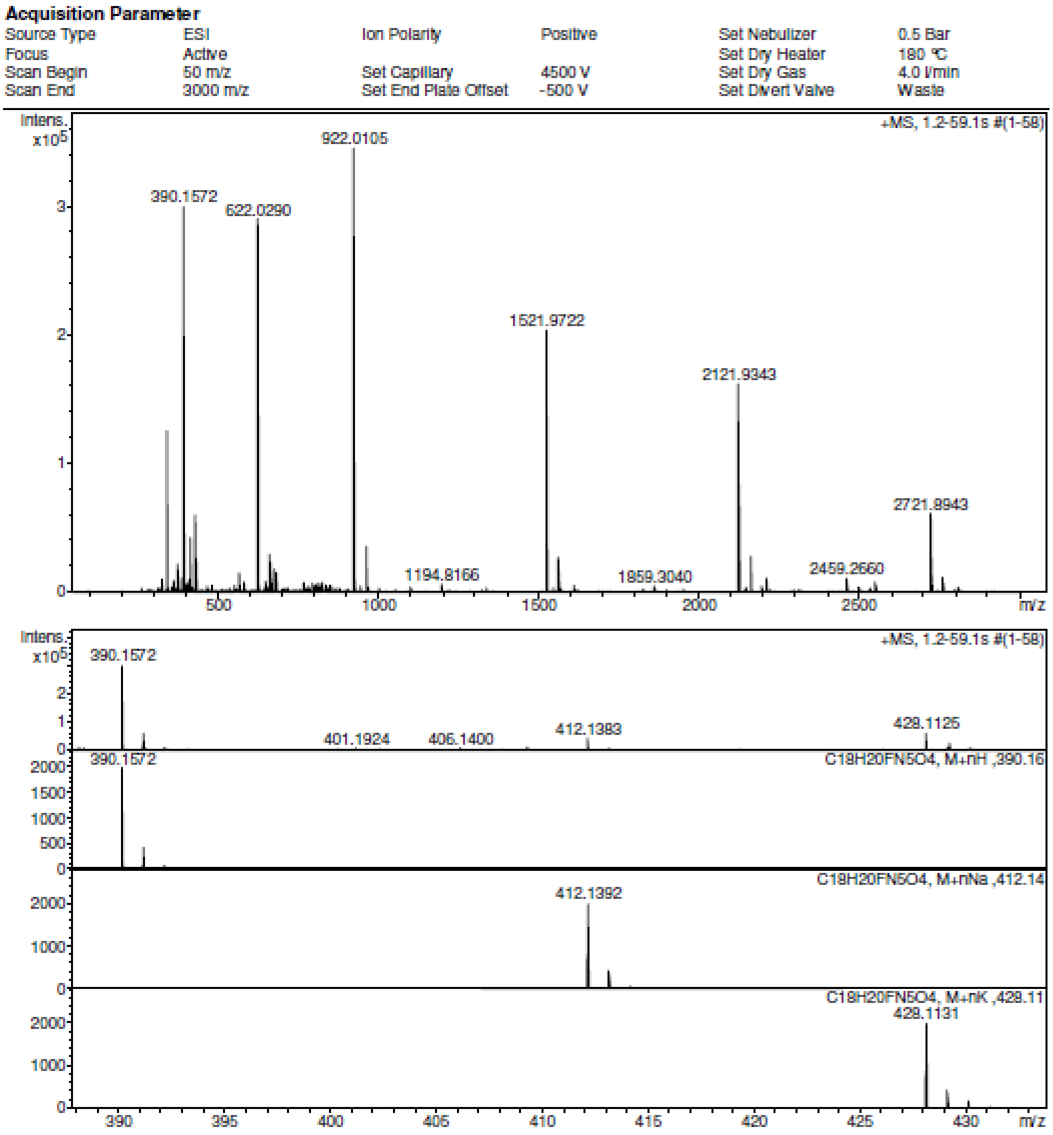

High-resolution mass spectrum (HRMS) of 2-fluoro, N^6-((R)-α-methylbenzyl)adenosine (**13a**)

Acquisition Parameter

Source Type	ESI	Ion Polarity	Positive	Set Nebulizer	0.4 Bar
Focus	Not active			Set Dry Heater	180 °C
Scan Begin	50 m/z	Set Capillary	4500 V	Set Dry Gas	4.0 l/min
Scan End	3000 m/z	Set End Plate Offset	-500 V	Set Divert Valve	Waste

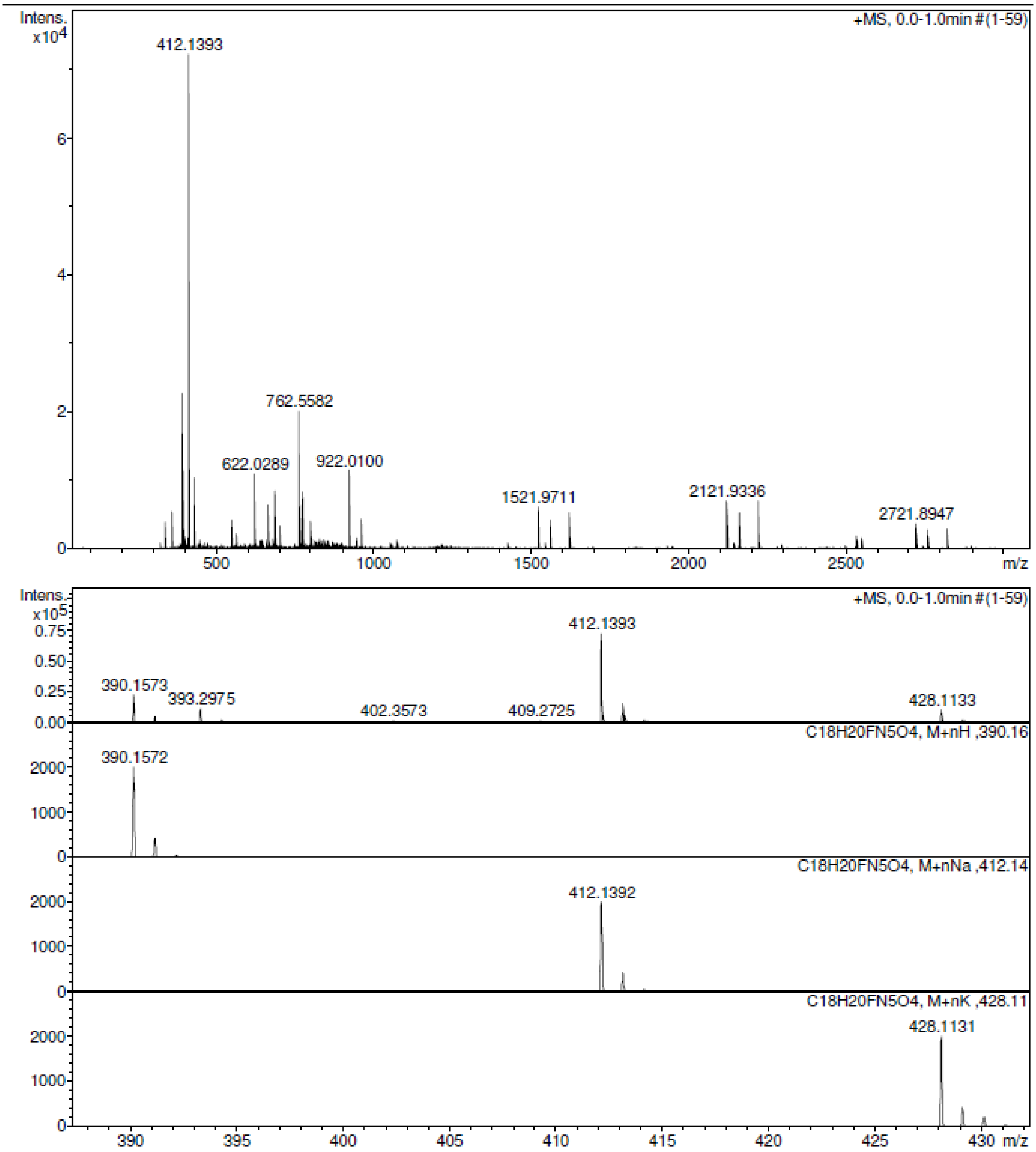

High-resolution mass spectrumof (HRMS) of 2-fluoro, N^6-((S)-α-methylbenzyl)adenosine (**13b**)

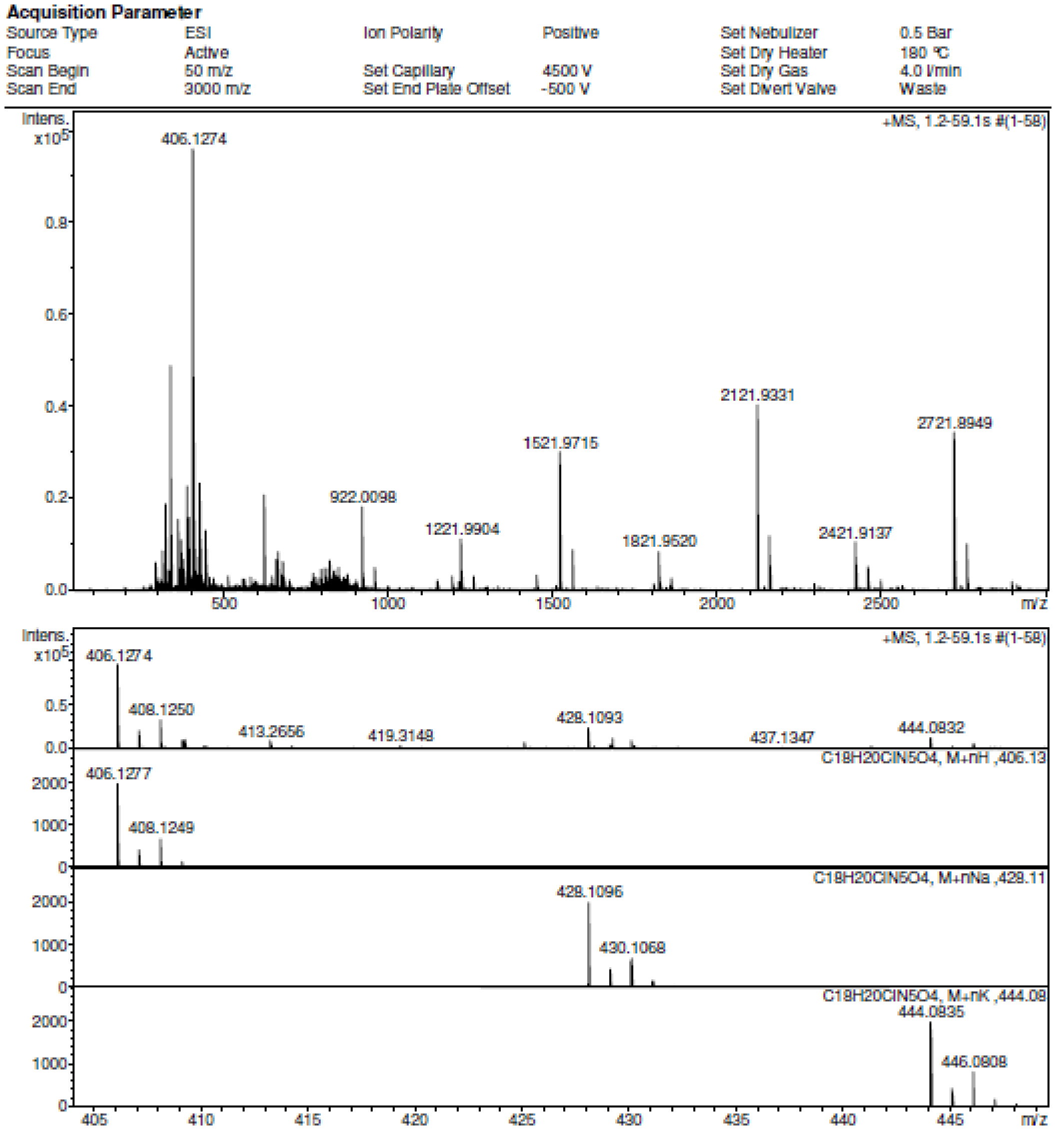

High-resolution mass spectrum (HRMS) of 2-chloro, N^6-((R)-α-methylbenzyl)adenosine (**14a**)

Acquisition Parameter

Source Type	ESI	Ion Polarity	Positive	Set Nebulizer	0.5 Bar
Focus	Active			Set Dry Heater	180 °C
Scan Begin	50 m/z	Set Capillary	4500 V	Set Dry Gas	4.0 l/min
Scan End	3000 m/z	Set End Plate Offset	-500 V	Set Divert Valve	Waste

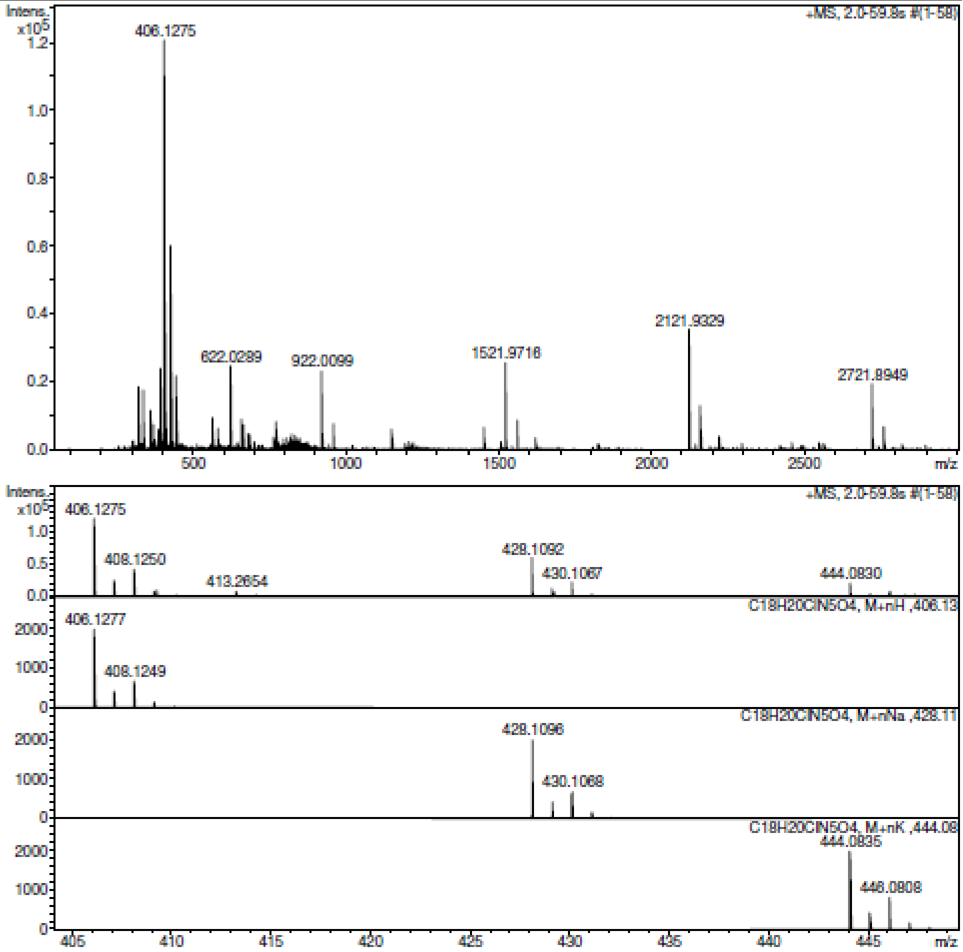

High-resolution mass spectrum (HRMS) of 2-chloro, N^6-((S)-α-methylbenzyl)adenosine (**14b**)

Acquisition Parameter

Source Type	ESI	Ion Polarity	Positive	Set Nebulizer	0.4 Bar
Focus	Not active			Set Dry Heater	180 °C
Scan Begin	50 m/z	Set Capillary	4500 V	Set Dry Gas	4.0 l/min
Scan End	3000 m/z	Set End Plate Offset	-500 V	Set Divert Valve	Waste

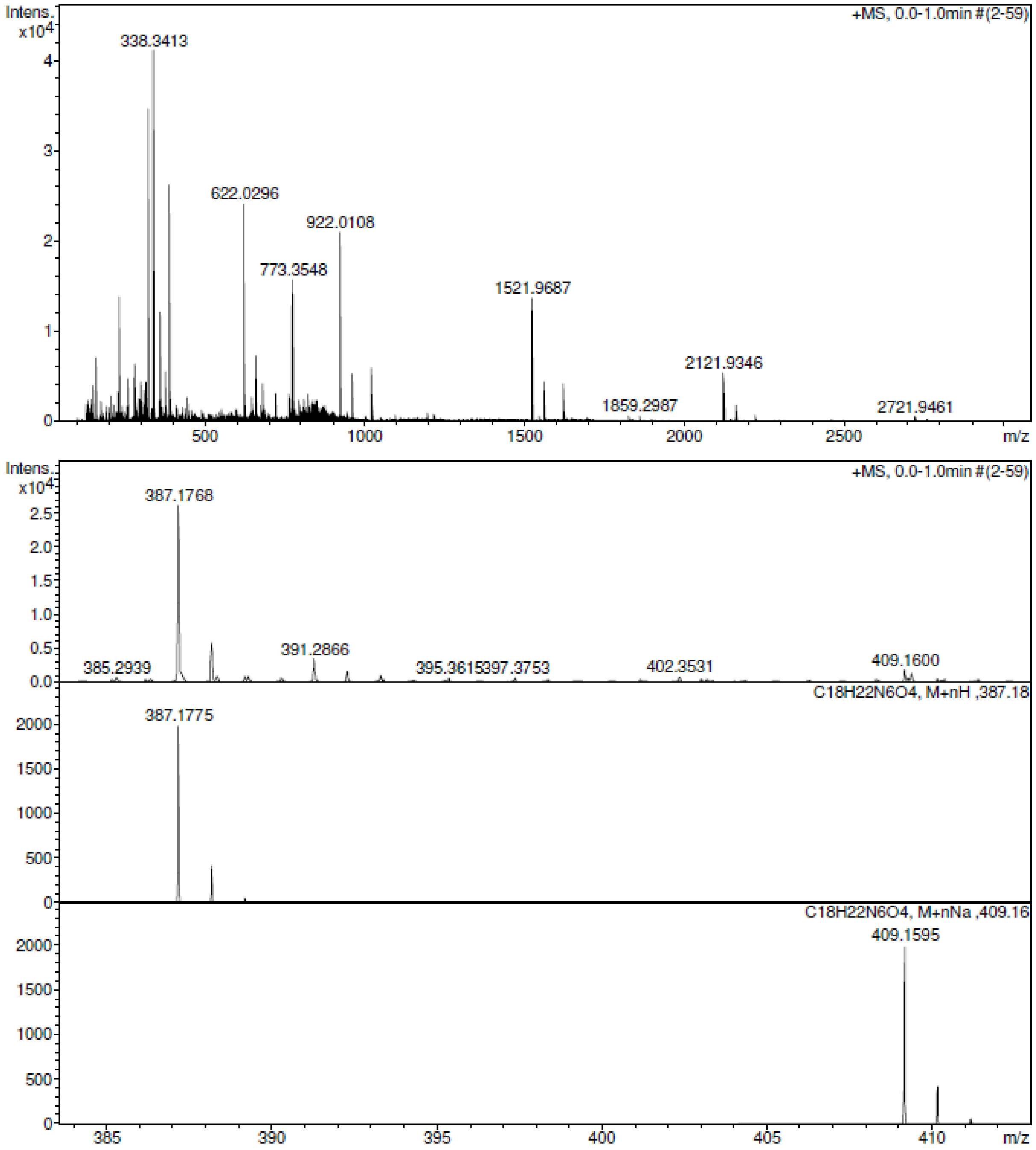

HRMS-spectrum of 2-amino, *N*[6]-((R)-α-methylbenzyl)adenosine (**16a**)

Acquisition Parameter

Source Type	ESI	Ion Polarity	Positive	Set Nebulizer	0.4 Bar
Focus	Not active			Set Dry Heater	180 °C
Scan Begin	50 m/z	Set Capillary	4500 V	Set Dry Gas	4.0 l/min
Scan End	3000 m/z	Set End Plate Offset	-500 V	Set Divert Valve	Waste

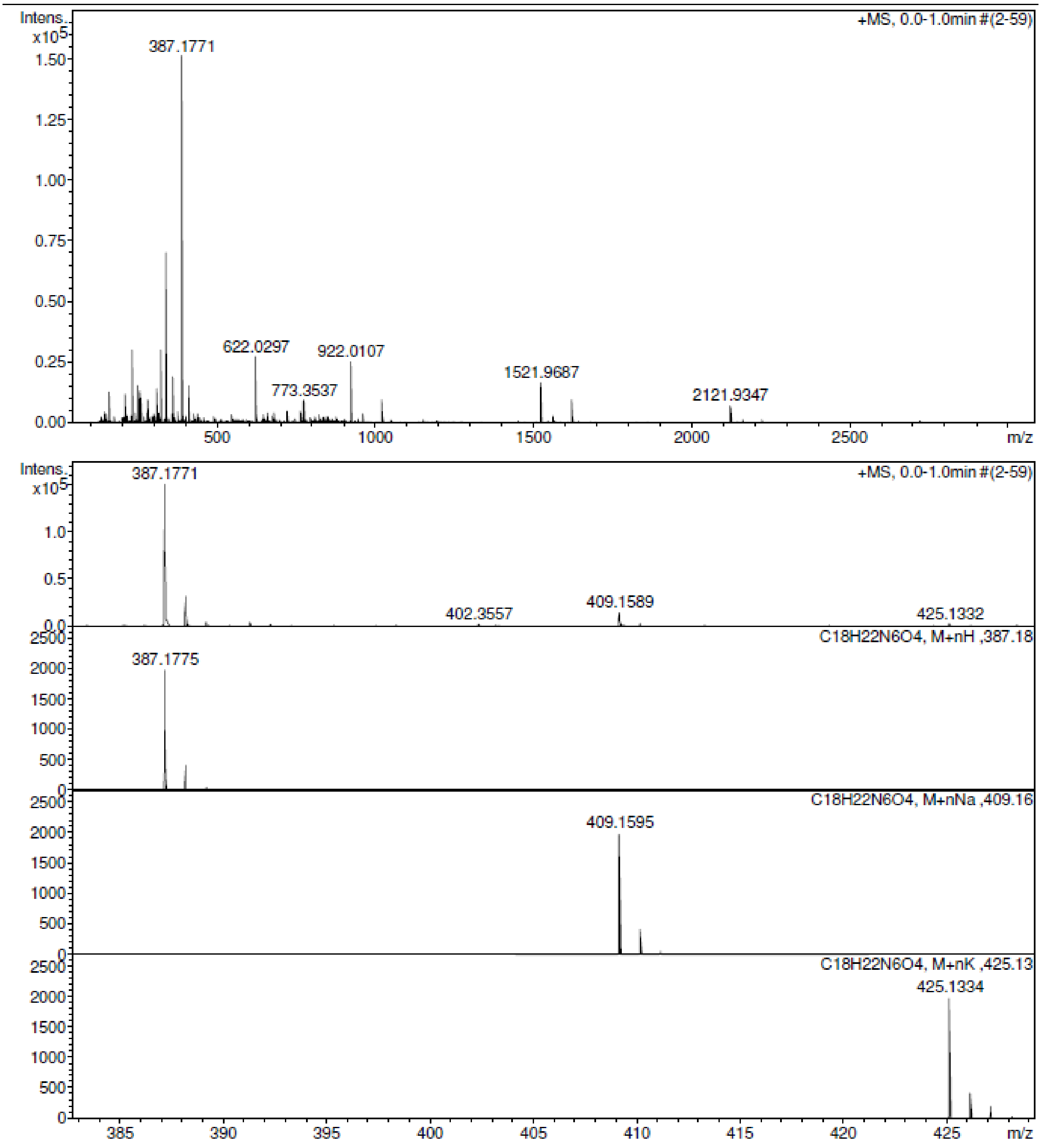

HRMS-spectrum of 2-amino, N^6-((S)-α-methylbenzyl)adenosine (**16b**)

Acquisition Parameter

Source Type	ESI	Ion Polarity	Positive	Set Nebulizer	0.4 Bar
Focus	Not active			Set Dry Heater	180 °C
Scan Begin	50 m/z	Set Capillary	4500 V	Set Dry Gas	4.0 l/min
Scan End	3000 m/z	Set End Plate Offset	-500 V	Set Divert Valve	Waste

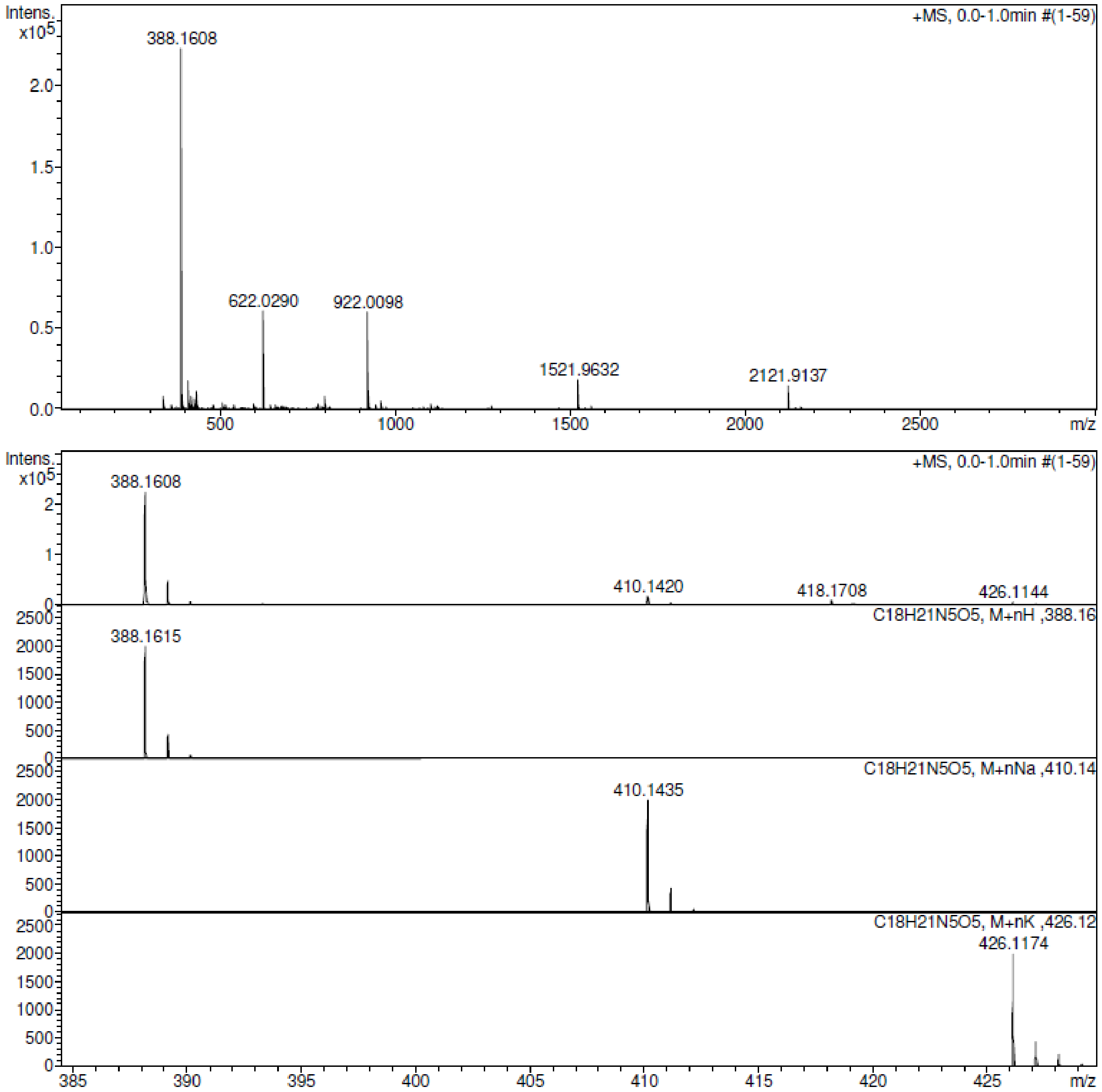

HRMS-spectrum of N^6-(benzyloxymethyl)adenosine (**Ado$^{\mathbf{BOM}}$**)

Acquisition Parameter

Source Type	ESI	Ion Polarity	Positive	Set Nebulizer	0.4 Bar
Focus	Not active			Set Dry Heater	180 °C
Scan Begin	50 m/z	Set Capillary	4500 V	Set Dry Gas	4.0 l/min
Scan End	3000 m/z	Set End Plate Offset	-500 V	Set Divert Valve	Waste

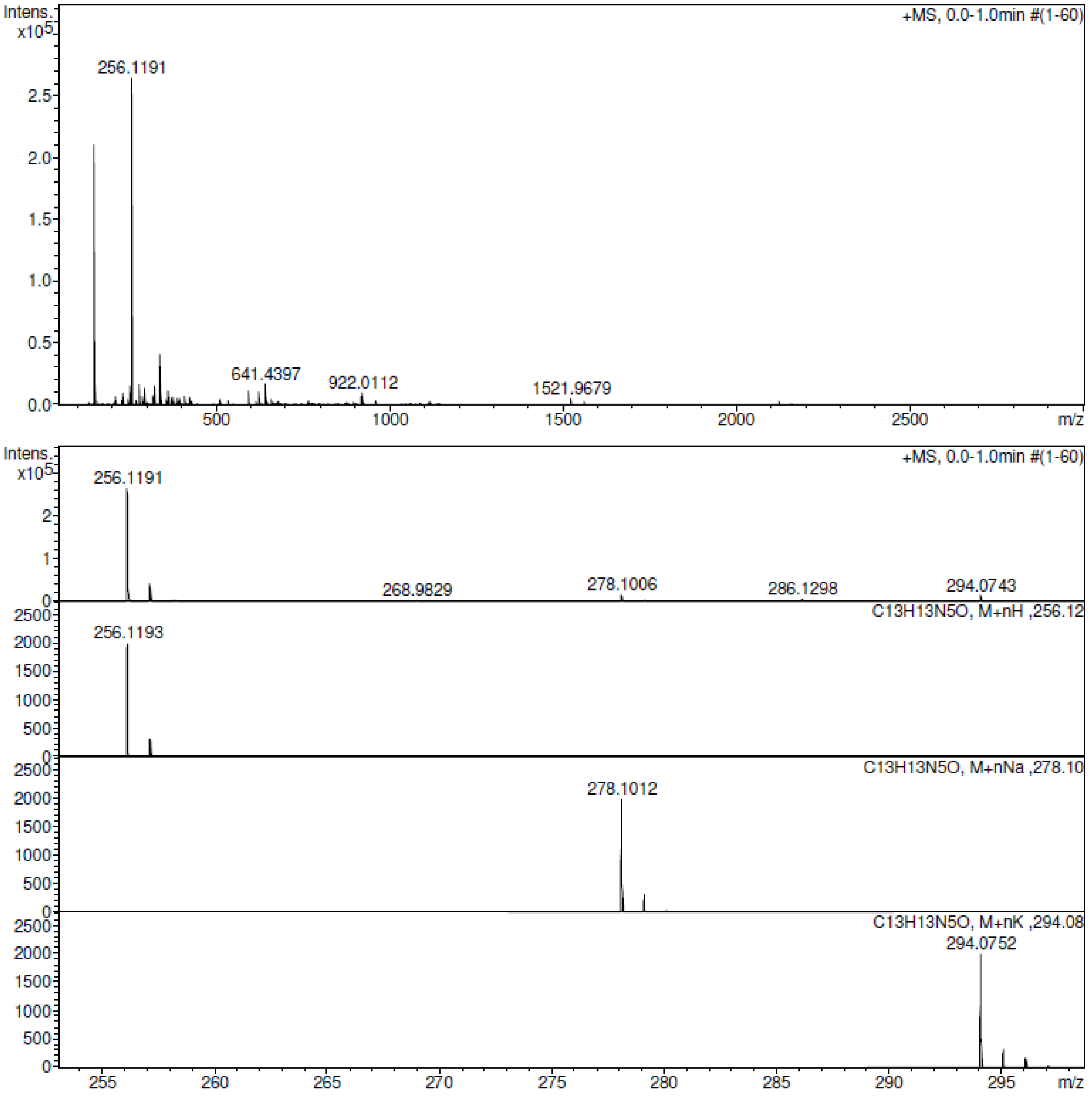

HRMS-spectrum of N^6-(benzyloxymethyl)adenine (**Ade$^{\mathbf{BOM}}$**)

5. Plant-based data

Supplementary tables

Table S1. GUS activity of BA derivatives (1 μM) in the biotest with double receptor mutants of *PARR5:GUS* Arabidopsis (% of 1 μM BA activity). Background values for each receptor as % of 1 μM BA-induced GUS activity (11.8±2.1, 10.4±2.6 and 16.5±2.5 % for AHK2, AHK3 and CRE1/AHK4, respectively) were subtracted.

		AHK2	AHK3	CRE1/AHK4
R-nucleobases	5a	72.7 ± 7.4	67.8 ± 10.9	73.3 ± 3.6
	6a	86.7 ± 5.2	99.2 ± 10.4	115.8 ± 4.4
	7a	24.5 ± 6.7	65.4 ± 4.5	78.7 ±5.1
	8a	32.4 ± 2.4	19.9 ± 3.1	33.7 ± 6.0
S-nucleobases	5b	0.0 ± 4.4	33.0 ± 4.7	0.0 ± 3.0
	6b	0.0 ± 2.2	86.1 ± 3.8	8.3 ± 2.5
	7b	0.0 ± 2.6	81.3 ± 4.0	0.0 ± 2.5
	8b	0.0 ± 2.5	0.0 ± 2.6	0.0 ± 3.9
R-ribonucleosides	12a	14.9 ± 5.0	23.9 ± 3.5	5.5 ± 1.6
	13a	0.0 ± 2.1	15.8 ± 3.5	9.0 ± 5.1
	14a	0.0 ± 2.1	7.8 ± 3.0	0.0 ± 5.1
	16a	0.0 ± 2.4	17.9 ± 5.9	7.4 ± 6.6
S-ribonucleosides	12b	0.0 ± 2.2	12.7 ± 2.6	0.0 ± 2.6
	13b	2.2 ± 3.3	12.2 ± 4.1	0.0 ± 5.3
	14b	2.7 ± 3.2	1.1 ± 2.6	0.0 ± 5.7
	16b	0.0 ± 2.6	5.8 ± 2.6	0.0 ± 2.5

0 **150**

Table S2. Correlation coefficients (Pearson algorithm) between activities of ribosides and nucleobases.

	R-nucleobases 1 µM	*S*-nucleobases 1 µM	*S*-nucleobases 50 µM
R- ribonucleosides 1 µM	0.10	0.37	0.23
S- ribonucleosides 1 µM	0.13	**0.58**	0.42
R-ribonucleosides 50 µM	**0.59**	0.05	0.27
S-ribonucleosides 50 µM	-0.10	**0.71**	0.39

Table S3. GUS activity of $\mathbf{Ado^{BOM}}$ and $\mathbf{Ade^{BOM}}$ (50 µM) in a mixture with BA (0.1 µM) in biotest with double receptor mutants of P_{ARR5}:*GUS* Arabidopsis, % from activity of BA 0.1 µM.

	**AHK2**	**AHK3**	**CRE1/AHK4**
Ado^{BOM}	60.1 ± 4.6	> 100%	52.2 ± 6.9
Ade^{BOM}	> 100%	> 100%	> 100%

Supplementary figures

Figure S1. See, please, p. 19 of the Supplementary Materials file.

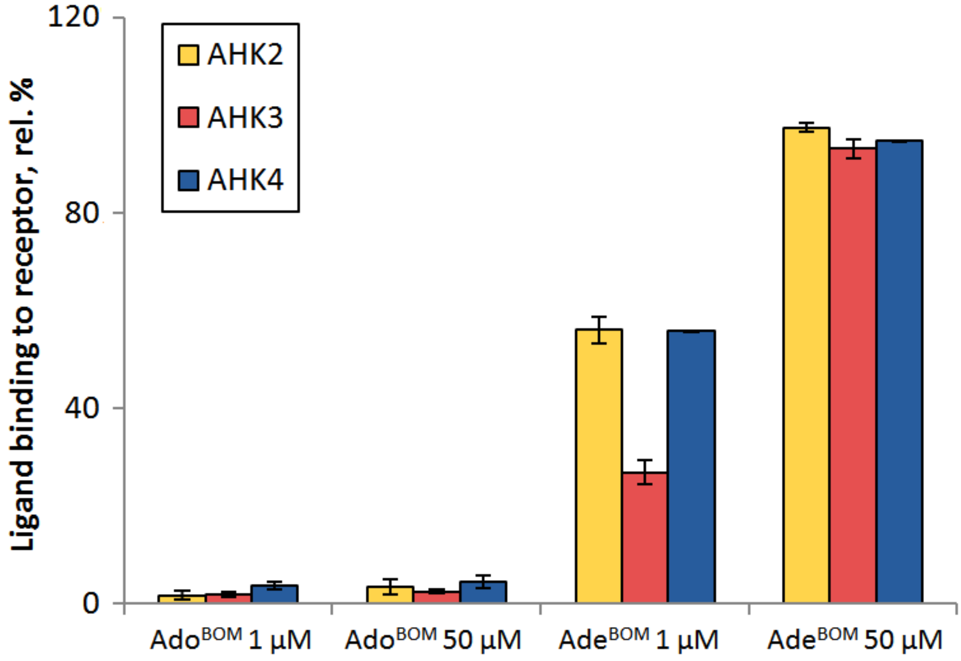

Figure S2. Specific binding activity of **Ado^{BOM}** and **Ade^{BOM}** to individual cytokinin receptors of Arabidopsis, % of total binding.

6. Molecular modeling and docking

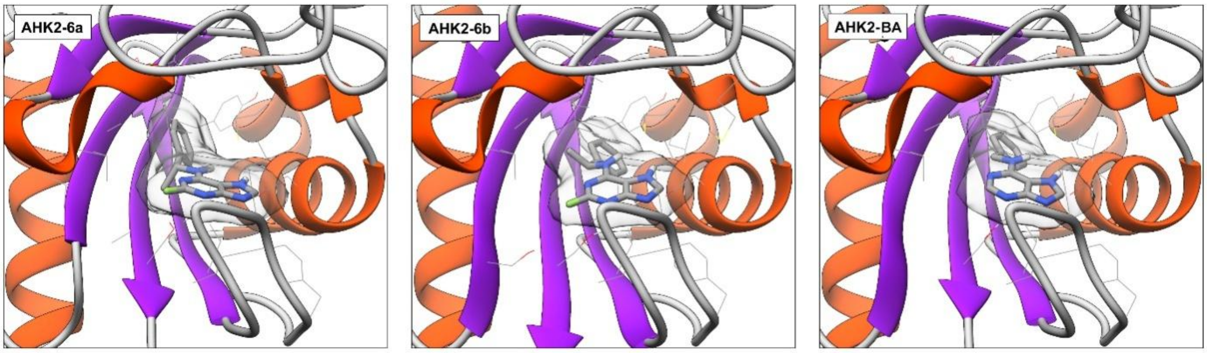

Figure S3. Molecular docking of compounds **6a** and **6b** to AHK2 receptor, compared with the control (BA).

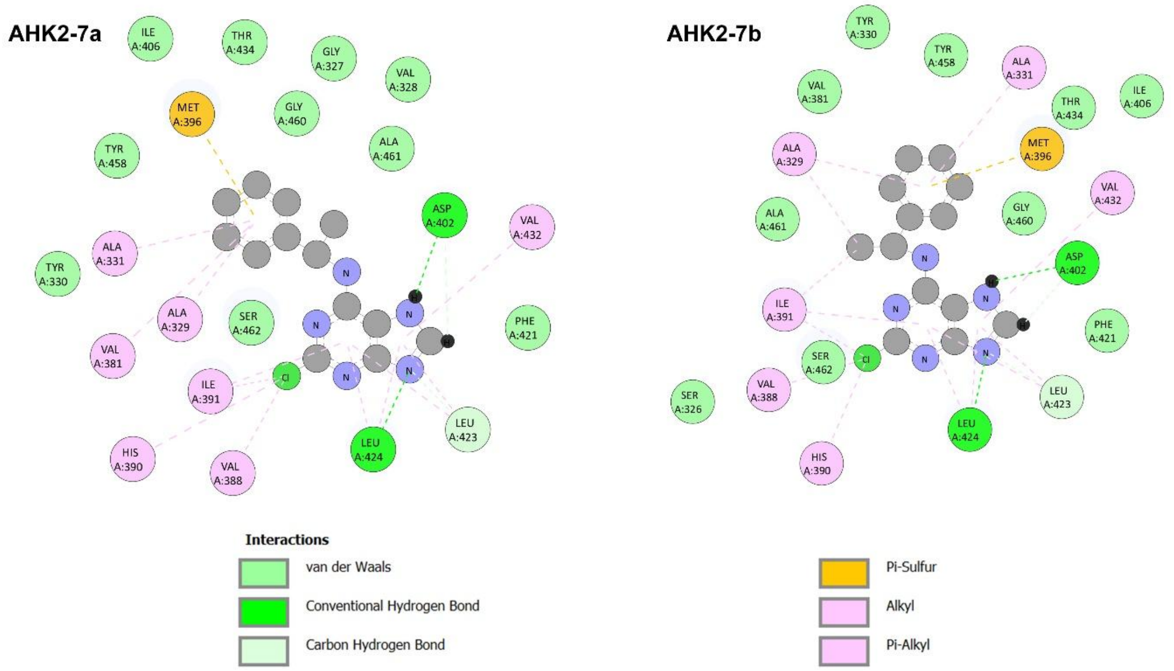

Figure S4. Interactions of compounds **7a** and **7b** with AHK2 receptor visualized with the Discovery Studio Visualizer.

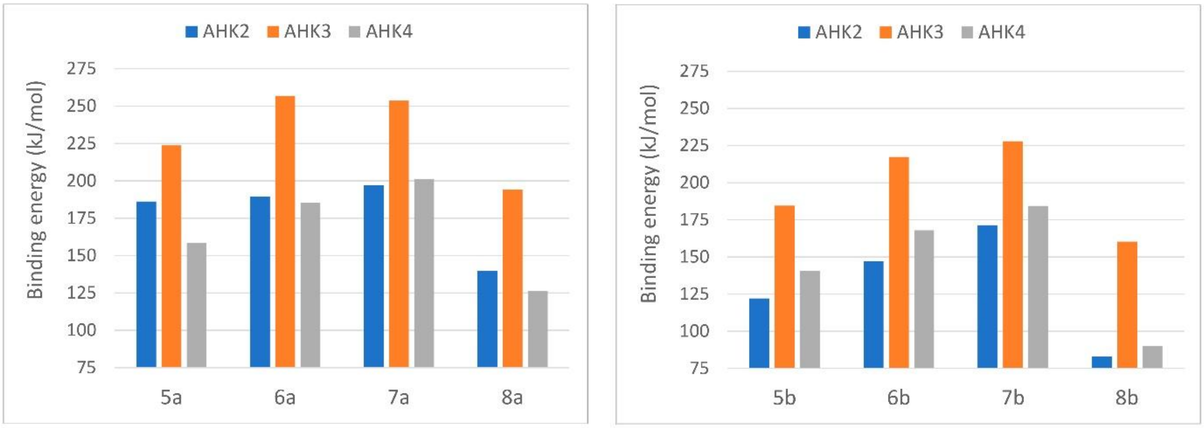

Figure S5. Energy of protein-ligand interactions of AHK2-4 receptors with chiral isomers of BA derivatives calculated by the PBS method in YASARA. According to the YASARA calculation algorithms, more positive energy values mean stronger binding.

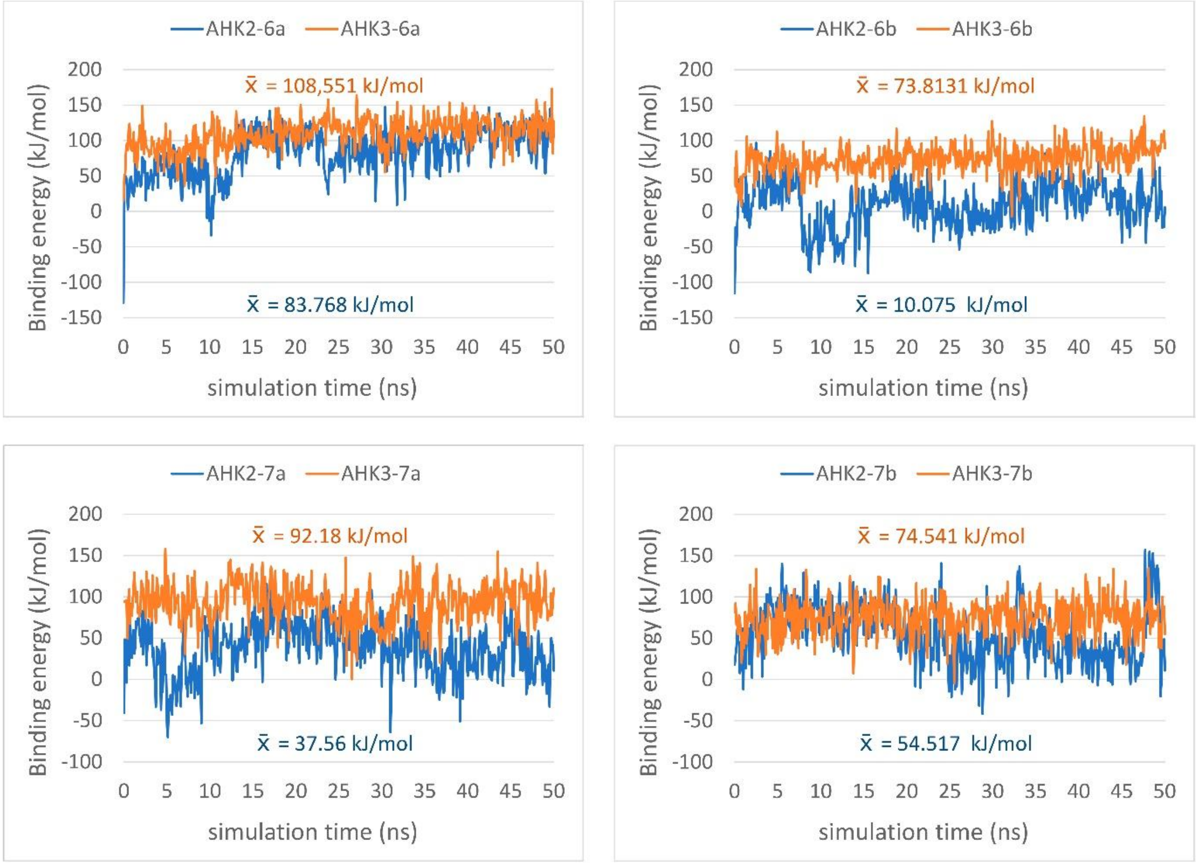

Figure S6. Comparison of BA-derivatives (**6a**/**b**, **7a**/**b**) binding energies between AHK2 and AHK3 receptors, calculated by analyzing the MD trajectory by the BoundaryFast method in YASARA. According to the YASARA calculation algorithms, more positive energy values mean stronger binding.

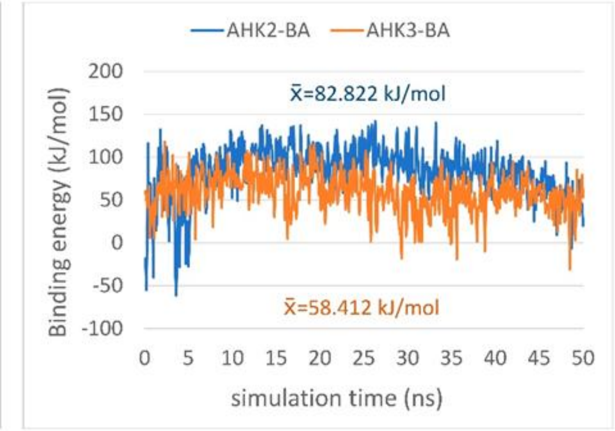

Figure S7. Comparison of BA binding energies between AHK2 and AHK3 receptors, calculated by analyzing the MD trajectory by the BoundaryFast method in YASARA. According to the YASARA calculation algorithms, more positive energy values mean stronger binding.

Table S4. Van der Waals volumes of compounds and their substituents at *C*2.

Compound/ radical	Van der Waals volume (Å^3)
5a	228,088
5b	228,096
6a	231,136
6b	231,32
7a	243,984
7b	243,8
8a	238,424
8b	238,424
BA	211,384
H	7,2
F	13,064
Cl	25,152
NH2	22,352

Van der Waals volumes were calculated using MoloVol 1.0.0 [76]. Small probe radius was set to 1.2 Å. Grid resolution was 0.2 Å and optimization depth was set to 4.
